# Identification of 31 loci for mammographic density phenotypes and their associations with breast cancer risk

**DOI:** 10.1038/s41467-020-18883-x

**Published:** 2020-10-09

**Authors:** Weiva Sieh, Joseph H. Rothstein, Robert J. Klein, Stacey E. Alexeeff, Lori C. Sakoda, Eric Jorgenson, Russell B. McBride, Rebecca E. Graff, Valerie McGuire, Ninah Achacoso, Luana Acton, Rhea Y. Liang, Jafi A. Lipson, Daniel L. Rubin, Martin J. Yaffe, Douglas F. Easton, Catherine Schaefer, Neil Risch, Alice S. Whittemore, Laurel A. Habel

**Affiliations:** 1 Department of Population Health Science and Policy, Icahn School of Medicine at Mount Sinai, New York, NY USA; 2 Department of Genetics and Genomic Sciences, Icahn School of Medicine at Mount Sinai, New York, NY USA; 3 Division of Research, Kaiser Permanente Northern California, Oakland, CA USA; 4 Department of Pathology, Icahn School of Medicine at Mount Sinai, New York, NY USA; 5 Department of Epidemiology and Biostatistics, University of California San Francisco, San Francisco, CA USA; 6 Department of Epidemiology and Population Health, Stanford University School of Medicine, Stanford, CA USA; 7 Department of Radiology, Stanford University School of Medicine, Stanford, CA USA; 8 Department of Biomedical Data Science, Stanford University School of Medicine, Stanford, CA USA; 9 Department of Medicine, Stanford University School of Medicine, Stanford, CA USA; 10 Departments of Medical Biophysics and Medical Imaging, University of Toronto, Toronto, ON Canada; 11 Centre for Cancer Genetic Epidemiology, Department of Public Health and Primary Care and Department of Oncology, University of Cambridge, Cambridge, UK; 12 Institute for Human Genetics, University of California San Francisco, San Francisco, CA USA

**Keywords:** Genome-wide association studies, Breast cancer, Cancer epidemiology

## Abstract

Mammographic density (MD) phenotypes are strongly associated with breast cancer risk and highly heritable. In this GWAS meta-analysis of 24,192 women, we identify 31 MD loci at *P* < 5 × 10^−8^, tripling the number known to 46. Seventeen identified MD loci also are associated with breast cancer risk in an independent meta-analysis (*P* < 0.05). Mendelian randomization analyses show that genetic estimates of dense area (DA), nondense area (NDA), and percent density (PD) are all significantly associated with breast cancer risk (*P* < 0.05). Pathway analyses reveal distinct biological processes involving DA, NDA and PD loci. These findings provide additional insights into the genetic basis of MD phenotypes and their associations with breast cancer risk.

## Abstract (web summary)

Mammographic density represents one the strongest predictors of breast cancer risk. Here the authors perform genome-wide association study meta-analysis of women screened with full-field digital mammography and identify 31 previously unreported loci associated with mammographic density phenotypes.

## Introduction

Percent density (PD), the percentage of the breast area that appears radiodense or light on a mammogram, is one of the strongest risk factors for breast cancer, but the biological basis for this association is poorly understood^1,2^. Women with ≥75% density on a mammogram have a 4 to 5-fold increased risk of breast cancer compared to women with little or no dense tissue, independent of other known risk factors^2,3^. PD is a composite of two phenotypes: the dense area (DA) reflecting the amount of fibroglandular tissue in the breast, and the nondense area (NDA) consisting of predominantly fatty tissues that appear radiotranslucent or dark on a mammogram^4^. Recent studies have shown that NDA is associated with decreased breast cancer risk independently of DA, suggesting that breast adipose tissues play an important role in normal mammary gland growth and function^5,6^. PD, DA, and NDA each have heritability estimates of over 50% in twin studies^7–9^. However, only 15 independent genome-wide significant loci with *P* < 5 × 10^−8^ have been identified to date, together explaining less than 1-3% of the total variance of mammographic density (MD) phenotypes^10–14^.

In this genome-wide association study (GWAS) meta-analysis of 24,192 women screened with full-field digital mammography (FFDM), we identify 31 MD loci, of which 17 also are associated with breast cancer in an independent study of over 200,000 breast cancer cases and controls^15^. These findings triple the total number of independent genome-wide significant MD loci now mapped to 46, enabling the first genetic pathway analyses and Mendelian randomization analyses to evaluate the causal nature of the association of MD phenotypes with breast cancer risk. Pathway analyses reveal distinct biological processes involving DA, NDA, or PD loci. Mendelian randomization analyses show that genetic estimates of DA, NDA, and PD are all significantly associated with breast cancer risk. These findings provide additional insights into the genetic basis of MD phenotypes and their relationship with breast cancer risk.

## Results

### GWAS of MD phenotypes

This GWAS meta-analysis comprised a total of 24,192 non-Hispanic white women with MD phenotypes measured centrally using Cumulus software^16^. The first study included 20,311 women screened using Hologic FFDM machines, and the second study included an independent sample of 3881 women screened using General Electric (GE) FFDM machines. Women in the GE cohort were 2.7 years younger, had lower BMI, and were less likely to be postmenopausal compared with women in the Hologic cohort (Supplementary Table 1). On average, DA was 1.1 cm^2^ higher, NDA 31.2 cm^2^ lower, and PD 4.0 percentage points higher in the GE cohort compared with the Hologic cohort (Supplementary Fig. 1). PD, computed by DA divided by the total breast area (DA + NDA), was strongly correlated with DA (*R*, 0.8) and NDA (*R,* −0.8), and DA was moderately negatively correlated with NDA (*R*, −0.35) in both cohorts, as expected.

**Fig. 1:**
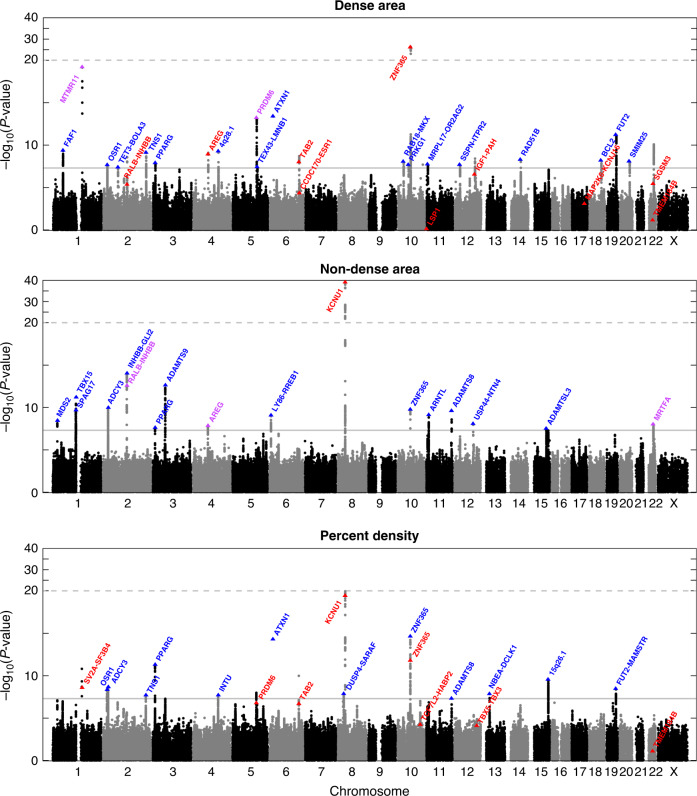
Manhattan plots for the GWAS meta-analysis of the standardized mammographic density phenotypes for 24,192 women with Hologic or GE full-field digital mammography exams. Blue denotes new loci, red denotes previously identified loci, and purple denotes new phenotypic associations at previously identified loci. A new DA SNP in *MTMR11* was in linkage disequilibrium (*r*^*2*^ = 0.85) with a previously identified PD SNP in between *SV2A* and *SF3B4*. A new NDA SNP in *MRTFA* was in linkage disequilibrium (*r*^*2*^ = 0.39) with a previously identified DA SNP in *SGSM3*. The *ZNF365* locus contains two independent subregions (*r*^*2*^ = 0.07) associated with DA or NDA, respectively, as well as with PD.

In the Hologic study, 37 SNPs were associated with MD phenotypes at *P* < 5 × 10^−8^. In the GE study, 3 of these SNPs could not be confirmed and were excluded, while 18 additional SNPs with *P* < 5 × 10^−5^ in the Hologic study reached genome-wide significance in the combined meta-analysis. In total, 52 SNPs at 40 independent chromosomal regions (loci) were associated with DA, NDA, and/or PD in the same directions in both studies, and met the conventional genome-wide significance threshold of *P* < 5 × 10^−8^ (Fig. 1). The genomic inflation factors for the GWAS meta-analyses of DA, NDA, and PD were 1.06, 1.08, and 1.07, respectively, indicating that there was little evidence of uncontrolled population substructure (Supplementary Fig. 2).

We identified 39 previously unreported MD SNPs at 31 independent loci, including 16 SNPs for DA, 13 for NDA, and 12 for PD with *P* < 5 × 10^−8^ in the GWAS meta-analysis, adjusting for ln(BMI), age at mammography, and principal components of European ancestry (Table 1, Supplementary Table 5). A single SNP showed the strongest association with both DA and PD at two loci (2q35, 6p22.3). Of the 16 DA loci, one (3p25.2) was also significantly associated with both NDA and PD, five (2p24.1, 2q35, 4q28.1, 6p22.3, 19q13.33) were also associated with PD but not NDA, and 10 were associated with DA only. Of the 13 NDA loci, one (3p25.2) was also significantly associated with both DA and PD, three (2p23.3, 10q21.2(1), 11q24.3) were also associated with PD but not DA, and nine were associated with NDA only. Sensitivity analyses of the NDA SNPs showed that all 13 remained significant after additional adjustment for BMI using three polynomial terms (BMI, BMI^2^, and BMI^3^) in addition to ln(BMI); and only 2 of the 13 NDA SNPs (rs6718628 and rs4132228) were associated with BMI at *P* < 0.05 in models unadjusted for BMI (Supplementary Table 13). Of the 12 PD loci, nine were also significantly associated with DA and/or NDA, and three (8p12, 13q13.3, 15q26.1) were associated with PD only. These three SNPs had opposite directions of association with DA and NDA that were not genome-wide significant when analyzed separately, but reached statistical significance for the PD composite measure of DA and NDA. Analyses stratified by menopausal status showed that MD SNP effects were similar in premenopausal and postmenopausal women (Supplementary Table 15).

**Table 1:** Genome-wide significant loci for mammographic density phenotypes identified in the meta-analysis of Hologic and GE studies.

Region^a^	Lead SNP	Position	Gene^b^	Ref/Alt	AAF	Hologic (*N* = 20,311)			GE (*N* = 3881)			Combined (*N* = 24,192)		
						Beta	SE	*P* value	Beta	SE	*P value*	Beta	SE	*P value*
*Dense area (*n* = 16)*														
1p32.3	rs6703250	51418472	*FAF1*	C/T	0.55	−0.052	0.009	9.8E–08	−0.068	0.022	2.0E–03	−0.054	0.009	4.8E–10
2p24.1	rs11684853	19310918	*OSR1*	G/T	0.55	0.049	0.009	2.0E–07	0.047	0.022	3.1E–02	0.049	0.009	2.5E–08
2p13.1	rs6715731	74346404	*TET3, BOLA3*	G/T	0.42	−0.055	0.009	7.7E–09	−0.006	0.022	8.0E–01	−0.048	0.009	4.7E–08
2q35	rs66470855*	218266438	*TNS1*	TG/T	0.26	0.061	0.011	1.3E–08	0.058	0.025	2.0E–02	0.060	0.010	8.5E–10
3p25.2	rs67901221	12416550	*PPARG*	A/G	0.15	0.079	0.013	1.5E–09	0.012	0.030	6.8E–01	0.069	0.012	1.4E–08
4q28.1	rs1503613	127772368	*—*	G/T	0.47	0.059	0.009	4.5E–10	0.029	0.022	1.9E–01	0.054	0.009	6.1E–10
5q23.2(2)	rs6885843	126068500	*TEX43, LMNB1*	C/T	0.40	0.051	0.009	1.2E–07	0.032	0.022	1.5E–01	0.048	0.009	4.3E–08
6p22.3	rs3819405*	16399557	*ATXN1*	C/T	0.33	−0.069	0.010	3.7E–12	−0.067	0.023	3.8E–03	−0.069	0.009	4.4E–14
10p12.1	rs2642278	27936562	*RAB18, MKX*	T/C	0.36	0.052	0.009	6.5E–08	0.037	0.023	1.0E–01	0.050	0.009	1.0E–08
10q21.1	rs1892368	53674795	*PRKG1*	G/A	0.67	−0.043	0.010	1.3E–05	−0.090	0.023	9.0E–05	−0.051	0.009	2.4E–08
11p15.4	rs11040963	6713214	*MRPL17, OR2AG2*	C/A	0.28	−0.052	0.010	7.8E–07	−0.059	0.024	1.5E–02	−0.053	0.010	2.1E–08
12p12.1	rs1818476	26445564	*SSPN, ITPR2*	C/T	0.75	−0.049	0.011	7.2E–06	−0.089	0.025	4.0E–04	−0.055	0.010	2.4E–08
14q24.1	rs75197674	68971452	*RAD51B*	T/C	0.21	−0.064	0.012	1.2E–07	−0.065	0.028	2.1E–02	−0.064	0.011	5.9E–09
18q21.33	rs11877925	60896694	*BCL2*	G/A	0.22	−0.053	0.011	2.6E–06	−0.093	0.026	3.4E–04	−0.060	0.010	7.4E–09
19q13.33	rs492602	49206417	*FUT2*	A/G	0.48	−0.055	0.009	4.7E–09	−0.084	0.022	1.5E–04	−0.060	0.009	6.9E–12
20q13.13	rs17196752	48887268	*SMIM25*	C/T	0.20	0.066	0.012	5.1E–08	0.050	0.028	7.0E–02	0.063	0.011	9.2E–09
*Non-dense area (*n* = 13)*														
1p36.11	rs61777307	23959319	*MDS2*	G/A	0.24	−0.040	0.008	2.8E–07	−0.055	0.018	2.1E–03	−0.042	0.007	3.5E–09
1p12(1)	rs10802015	118782659	*SPAG17*	C/T	0.34	−0.039	0.007	3.0E–08	−0.052	0.017	2.0E–03	−0.041	0.007	2.1E–10
1p12(2)	rs1779445	119508412	*TBX15*	T/C	0.80	0.056	0.008	1.1E–11	0.032	0.019	9.1E–02	0.052	0.008	6.2E–12
2p23.3	rs6718628	25131170	*ADCY3*	C/G	0.40	0.038	0.007	3.9E–08	0.056	0.016	6.5E–04	0.041	0.006	1.1E–10
2q14.2(2)	rs11123556	121245996	*INHBB, GLI2*	A/G	0.89	−0.071	0.011	2.2E–11	−0.098	0.025	7.1E–05	−0.075	0.010	9.2E–15
3p25.2	rs199689761	12441088	*PPARG*	T/TA	0.16	−0.048	0.009	1.2E–07	−0.037	0.021	7.9E–02	−0.047	0.008	2.4E–08
3p14.1	rs4132228	64708114	*ADAMTS9*	C/T	0.31	−0.045	0.007	2.8E–10	−0.062	0.016	1.5E–04	−0.048	0.007	2.3E–13
6p25.1	rs1294419	6742549	*LY86, RREB1*	C/G	0.62	0.039	0.007	8.0E–09	0.031	0.016	5.0E–02	0.038	0.006	8.3E–10
10q21.2(1)	rs1949355	64218423	*ZNF365*	G/A	0.56	−0.039	0.007	3.6E–09	−0.037	0.015	1.7E–02	−0.039	0.006	1.7E–10
11p15.2	rs60521023	13314102	*ARNTL*	A/AT	0.70	0.041	0.007	2.1E–08	0.042	0.017	1.3E–02	0.041	0.007	7.5E–10
11q24.3	rs2875238	130282078	*ADAMTS8*	T/C	0.66	0.046	0.007	1.0E–10	0.016	0.017	3.3E–01	0.041	0.007	2.5E–10
12q22	rs11836367	96027467	*USP44, NTN4*	C/T	0.33	0.045	0.008	2.0E–08	0.030	0.018	1.0E–01	0.042	0.007	8.4E–09
15q25.2	rs1812707	84547222	*ADAMTSL3*	C/T	0.50	0.032	0.007	1.8E–06	0.044	0.016	5.0E–03	0.034	0.006	3.1E–08
*Percent density (*n* = 12)*														
2p24.1	rs34331777	19441251	*OSR1*	CA/C	0.60	0.041	0.008	2.5E–07	0.052	0.019	5.4E–03	0.042	0.007	4.5E–09
2p23.3	rs11676272	25141538	*ADCY3*	A/G	0.47	−0.040	0.008	2.9E–07	−0.061	0.019	1.1E–03	−0.043	0.007	2.2E–09
2q35	rs66470855*	218266438	*TNS1*	TG/T	0.26	0.046	0.009	1.8E–07	0.043	0.021	4.1E–02	0.046	0.008	2.0E–08
3p25.2	rs76643909	12441367	*PPARG*	T/G	0.16	0.076	0.011	1.5E–12	0.029	0.025	2.6E–01	0.069	0.010	4.9E–12
4q28.1	rs35589286	128192680	*INTU*	G/C	0.29	0.045	0.009	2.1E–07	0.045	0.021	3.0E–02	0.045	0.008	2.0E–08
6p22.3	rs3819405*	16399557	*ATXN1*	C/T	0.33	−0.058	0.008	1.1E–12	−0.061	0.019	1.9E–03	−0.059	0.008	5.0E–15
8p12	rs6558136	29523123	*DUSP4, SARAF*	C/T	0.65	−0.038	0.008	3.8E–06	−0.070	0.019	3.2E–04	−0.042	0.008	1.4E–08
10q21.2(1)	rs2138555	64220494	*ZNF365*	A/G	0.41	−0.054	0.008	4.8E–12	−0.073	0.018	7.8E–05	−0.057	0.007	2.3E–15
11q24.3	rs6590455	130276427	*ADAMTS8*	T/C	0.65	−0.046	0.008	1.6E–08	−0.012	0.019	5.3E–01	−0.041	0.008	5.0E–08
13q13.3	rs10219975	36269125	*NBEA, DCLK1*	G/C	0.32	−0.047	0.009	5.8E–08	−0.032	0.020	1.1E–01	−0.045	0.008	1.4E–08
15q26.1	rs4777948	94298239	*—*	G/T	0.47	0.048	0.008	1.4E–09	0.032	0.019	8.4E–02	0.046	0.007	2.8E–10
19q13.33	rs1704773	49209566	*FUT2, MAMSTR*	A/G	0.49	−0.040	0.008	4.6E–07	−0.062	0.019	1.2E–03	−0.044	0.007	3.5E–09

^a^Independent regions within the same cytoband are denoted by (1) or (2) in order by position.

^b^Nearest flanking protein coding gene(s) within 500 kb of the lead SNP.

*Denotes lead SNPs associated with both DA and PD at the genome-wide significance level of *p*  <  5  ×  10^−8^.

We confirmed associations with SNPs at 13 of 15 previously identified MD loci^10–14^ at *P* < 0.05, nine of which reached genome-wide significance in this study (Supplementary Table 7). Of the two remaining prior loci, rs7289126 at the *TMEM184B* locus on 22q13.1 had suggestive associations with DA (*P* = 0.085) and PD (*P* = 0.083) in the same directions as previously reported^13^. However, rs3817198 at the *LSP1* locus on 11p15.5 showed no evidence of association, although the imputation accuracy was relatively low for this SNP (imputation *r*^*2*^ = 0.55). We also report new MD phenotype associations at *P* < 5 × 10^−8^ with SNPs at five prior loci. At the *MTMR11* locus on 1q21.1 previously associated with PD^12^, we found that rs11205303 was associated with both DA (*P* = 6.8 × 10^−20^) and PD (*P* = 1.5 × 10^−11^). At the *RALB/INHBB* locus on 2q14.2 recently associated with absolute dense volume^10^, we found that rs4849864 was associated with NDA (*P* = 2.6 × 10^−13^) and rs17625845 was associated with DA (*P* = 2.8 × 10^−9^). At the *AREG* locus on 4q13.3 previously associated with DA^13^, we found that rs71219402 was associated with NDA (*P* = 1.4 × 10^−8^) while rs149689338 was the lead SNP for DA (*P* = 8.2 × 10^−10^). At the *PRDM6* locus on 5q23.2 previously associated with PD^13^, we found that rs335160 was associated with DA (*P* = 6.7 × 10^−14^) while rs335143 was the lead SNP for PD (*P* = 1.0 × 10^−8^). At the *MRTFA* (or *MKL1*) locus on 22q13.1-22q13.2 previously associated with DA^13^, we found that rs73169057 was associated with NDA (*P* = 9.6 × 10^−9^) while rs6001984 was the lead SNP for DA (*P* = 8.3 × 10^−11^).

Conditional analyses adjusting for the lead MD SNP in each region identified two independent subregions within three different chromosomal locations. At 1p12, two conditionally independent NDA SNPs, rs10802015 and rs1779445 (*r*^*2*^ = 0.02), attained *P* values of 1.4 × 10^−10^ and 7.6 × 10^−9^, respectively, in a linear regression model that included both SNPs. rs10802015 near *SPAG17* (sperm associated antigen 17) is located 726 kb away from rs1779445, an intronic variant of *TBX15* (T-box 15) that was also a significant eQTL for *WARS2* (mitochondrial tryptophanyl tRNA synthetase 2) in mammary tissue. At 2q14.2, we found an NDA SNP rs11123556 at the *INHBB/GLI2* locus that was conditionally independent from the strongest NDA SNP rs4849864 (*r*^*2*^ = 0.002) at the *RALB/INHBB* locus previously associated only with absolute dense volume;^10^ the two NDA SNPs attained *p* values of 1.9 × 10^−14^ and 4.7 × 10^−13^, respectively, in a linear regression model that included both SNPs. Finally, at the *ZNF365* locus on 10q21.2, we found a NDA SNP rs1949355 that was uncorrelated with rs10995190 (*r*^*2*^ = 0.07) previously associated only with DA and PD (Supplementary Fig. 4I, Supplementary Table 7)^13,17^. rs1949355 remained significantly associated with NDA (*P* = 6.7 × 10^−13^) after adjusting for rs10995190, which was associated with DA (*P* = 9.4 × 10^−27^) and PD (*P* = 1.6 × 10^−12^) but not NDA (*P* = 0.12) in single-SNP models.

### Associations with breast cancer risk

We evaluated whether the newly identified MD loci were also associated with breast cancer risk using data from an independent sample of 122,977 cases and 105,974 controls of European ancestry from the Breast Cancer Association Consortium (BCAC) and Discovery, Biology and Risk of Inherited Variants in Breast Cancer Consortium (DRIVE)^15^. We found that 24 MD SNPs at 17 loci were associated with breast cancer risk at *P* < 0.05 (Table 2, Supplementary Table 6). Of these SNPs, 15 MD SNPs at 10 loci were in linkage disequilibrium with previously reported breast cancer susceptibility alleles^15^, and 9 SNPs at 7 loci (3p25.2, 5q23.2(2), 10p12.1, 10q21.1, 11p15.4, 19q13.33, 20q13.13) were associated with both MD and breast cancer risk.

**Table 2:** Associations of newly identified MD alleles with breast cancer (all, ER-positive, and ER-negative) in BCAC.

Phenotype, Region^a^	Lead SNP	Position	Ref/Alt	MD Phenotype			All Breast Cancer			ER + Breast Cancer			ER- Breast Cancer		
				*Beta*	*SE*	*P value*	*Beta*	*SE*	*P value*	*Beta*	*SE*	*P value*	*Beta*	*SE*	*P value*
*DA alleles*															
1p32.3	rs6703250	51418472	C/T	−0.054	0.009	4.8E–10	−0.033	0.006	**1.3E**–**07**	−0.037	0.008	**6.7E**–**07**	−0.016	0.012	1.7E–01
2p24.1	rs11684853	19310918	G/T	0.049	0.009	2.5E–08	−0.044	0.006	**1.1E**–**12**	−0.027	0.007	**2.8E**–**04**	−0.066	0.011	**6.5E**–**09**
2q35	rs66470855*	218266438	TG/T	0.060	0.010	8.5E–10	0.072	0.007	**3.2E**–**24**	0.071	0.008	**9.7E**–**18**	0.071	0.013	**4.8E**–**08**
*3p25.2*	*rs67901221*	*12416550*	*A/G*	*0.069*	*0.012*	*1.4E*–*08*	*0.019*	*0.009*	*3.1E*–*02*	*0.015*	*0.011*	*1.5E*–*01*	*0.028*	*0.016*	*7.9E*–*02*
*5q23.2(2)*	*rs6885843*	*126068500*	*C/T*	*0.048*	*0.009*	*4.3E*–*08*	*0.014*	*0.007*	*3.7E*–*02*	*0.023*	*0.008*	*5.3E*–*03*	*0.001*	*0.013*	*9.7E*–*01*
6p22.3	rs3819405*	16399557	C/T	−0.069	0.009	4.4E–14	−0.040	0.007	**1.7E**–**08**	−0.045	0.008	**6.5E**–**08**	−0.027	0.013	3.5E–02
*10p12.1*	*rs2642278*	*27936562*	*T/C*	*0.050*	*0.009*	*1.0E*–*08*	*0.017*	*0.007*	*7.1E-03*	*0.014*	*0.008*	*6.6E*–*02*	*0.010*	*0.012*	*3.8E*–*01*
*10q21.1*	*rs1892368*	*53674795*	*G/A*	−*0.051*	*0.009*	*2.4E*–*08*	−*0.034*	*0.007*	***4.8E***–***07***	−*0.033*	*0.008*	***2.6E***–***05***	−*0.033*	*0.012*	*6.1E*–*03*
*11p15.4*	*rs11040963*	*6713214*	*C/A*	−*0.053*	*0.010*	*2.1E*–*08*	−*0.024*	*0.007*	***4.5E***–***04***	−*0.018*	*0.008*	*2.9E*–*02*	−*0.030*	*0.013*	*1.6E*–*02*
14q24.1	rs75197674	68971452	T/C	−0.064	0.011	5.9E–09	−0.062	0.008	**8.3E**–**16**	−0.062	0.009	**2.2E**–**11**	−0.047	0.014	**9.5E**–**04**
*20q13.13*	*rs17196752*	*48887268*	*C/T*	*0.063*	*0.011*	*9.2E*–*09*	*0.019*	*0.008*	*1.5E*–*02*	*0.008*	*0.009*	*3.8E*–*01*	*0.044*	*0.014*	*1.7E*–*03*
*NDA alleles*															
2p23.3	rs6718628	25131170	C/G	0.041	0.006	1.1E–10	−0.042	0.007	**2.9E**–**10**	−0.036	0.008	**7.0E**–**06**	−0.059	0.012	**9.4E**–**07**
2q14.2(2)	rs11123556	121245996	A/G	−0.075	0.010	9.2E–15	0.095	0.010	**5.3E**–**20**	0.085	0.012	**1.0E**–**11**	0.115	0.019	**2.9E**–**09**
*3p25.2*	*rs199689761*	*12441088*	*T/TA*	−*0.047*	*0.008*	*2.4E*–*08*	*0.018*	*0.009*	*4.9E*–*02*	*0.015*	*0.011*	*1.7E*–*01*	*0.027*	*0.017*	*1.1E*–*01*
10q21.2(1)	rs1949355	64218423	G/A	−0.039	0.006	1.7E–10	0.043	0.006	**3.4E**–**12**	0.046	0.007	**5.5E**–**10**	0.022	0.011	4.9E–02
12q22	rs11836367	96027467	C/T	0.042	0.007	8.4E–09	−0.082	0.007	**3.6E**–**36**	−0.084	0.008	**3.8E**–**27**	−0.065	0.012	**5.4E**–**08**
*PD alleles*															
2p24.1	rs34331777	19441251	CA/C	0.042	0.007	4.5E–09	−0.033	0.006	**2.8E**–**07**	−0.026	0.008	**6.9E**–**04**	−0.035	0.012	3.2E–03
2p23.3	rs11676272	25141538	A/G	−0.043	0.007	2.2E–09	−0.040	0.007	**1.2E**–**09**	−0.033	0.008	**2.8E**–**05**	−0.054	0.012	**6.8E**–**06**
2q35	rs66470855*	218266438	TG/T	0.046	0.008	2.0E–08	0.072	0.007	**3.2E**–**24**	0.071	0.008	**9.7E**–**18**	0.071	0.013	**4.8E**–**08**
*3p25.2*	*rs76643909*	*12441367*	*T/G*	*0.069*	*0.010*	*4.9E*–*12*	*0.018*	*0.009*	*4.2E*–*02*	*0.013*	*0.011*	*2.2E*–*01*	*0.031*	*0.016*	*5.3E*–*02*
6p22.3	rs3819405*	16399557	C/T	−0.059	0.008	5.0E–15	−0.040	0.007	**1.7E**–**08**	−0.045	0.008	**6.5E–08**	−0.027	0.013	3.5E–02
8p12	rs6558136	29523123	C/T	−0.042	0.008	1.4E–08	−0.056	0.007	**5.9E**–**18**	−0.062	0.008	**9.3E–16**	−0.042	0.012	**3.6E**–**04**
10q21.2(1)	rs2138555	64220494	A/G	−0.057	0.007	2.3E–15	−0.044	0.006	**1.5E**–**12**	−0.047	0.008	**3.9E–10**	−0.030	0.011	8.7E–03
*19q13.33*	*rs1704773*	*49209566*	*A/G*	−*0.044*	*0.007*	*3.5E*–*09*	*0.012*	*0.006*	*4.8E*–*02*	*0.015*	*0.007*	*4.4E–02*	−*0.007*	*0.011*	*5.3E*–*01*

*Denotes SNPs associated with both DA and PD; Boldface denotes significant associations with breast cancer risk at the Bonferroni-corrected threshold of *p*  <  1.3  ×  10^−3^ accounting for the 39 MD alleles tested; Italics denote MD alleles not previously known to be associated with breast cancer. Source of breast cancer results: http://bcac.ccge.medschl.cam.ac.uk/bcacdata/

^a^Independent regions within the same cytoband are denoted by (1) or (2) in order by position.

To explore the extent to which DA, NDA, and PD may be associated with breast cancer through shared underlying genetic factors and biologic pathways^4^, we performed Mendelian randomization analyses using the weighted median method^18^ and summary statistics for all prior and new MD loci from this study and BCAC/DRIVE^15^. For each standard deviation (SD) increment in DA, NDA, and PD, the estimated odds ratios (95% confidence interval) for breast cancer were: 1.45 (1.30–1.61; *P* < 0.001), 0.84 (0.73–0.98; *P* = 0.029), and 1.68 (1.44–1.96; *P* < 0.001), respectively. Sensitivity analyses using mode-based estimates that are also robust to violations of the instrumental variable assumptions, but less powerful than the weighted median method showed similar results (Supplementary Table 14)^19^. These estimates were remarkably similar to a meta-analysis of 13 observational studies that reported adjusted odds ratios of breast cancer associated with each SD increment of DA, NDA, and PD of: 1.37 (1.29–1.47), 0.78 (0.71–0.86), and 1.52 (1.39–1.66) in premenopausal women; and 1.38 (1.31–1.44), 0.79 (0.73–0.85), and 1.53 (1.44–1.64) in post-menopausal women^6^. These findings support a biological basis for the positive association of DA and PD with breast cancer risk, and inverse association of NDA with breast cancer risk, in observational studies.

To complement the Mendelian randomization analyses, we estimated the genetic correlation between each MD phenotype and breast cancer based on all SNPs genome-wide. LD Score regression^20,21^ of 779,828 SNPs using summary statistics from this study and the BCAC/DRIVE breast cancer GWAS^15^ yielded estimates of 0.27 (*P* = 5.5 × 10^−6^), −0.14 (*P* = 0.014), and 0.27 (*P* = 7.7 × 10^−10^) for the genetic correlations of DA, NDA, and PD, respectively, with breast cancer. The significant positive genetic correlations of DA and PD with breast cancer, and significant inverse association of NDA with breast cancer were consistent with the Mendelian randomization results, as well as evidence from observational studies^6^, supporting the shared genetic bases of all three MD phenotypes and breast cancer.

### Functional analyses

To identify potentially functional variants at the 31 MD loci, we examined whether any of the lead SNPs were associated with: protein-coding variants (Supplementary Table 2); gene expression levels in mammary tissue, primary fibroblast cells, subcutaneous fat, visceral fat, or whole blood (Supplementary Table 3); or promoter and enhancer regions in mammary epithelial cells or mammary fibroblasts (Supplementary Table 4). Regional association plots showed the nearby genes and linkage disequilibrium patterns in Europeans for each of the newly identified loci for DA, NDA, and PD (Supplementary Figs. 3–5).

MD SNPs at three loci (2p23.3, 15q25.2, 19q13.33) were strongly correlated (*r*^*2*^ ≥ 0.80) with nonsynonymous mutations, and all of these SNPs were also significant (FDR < 0.05) expression quantitative trait loci (eQTLs) (Supplementary Tables 2 and 3). SNPs on 2p23.3 were associated with NDA and PD. The lead PD SNP rs11676272 encoded the S107P missense mutation in *ADCY3* (adenylate cyclase 3), which catalyzes the formation of the secondary messenger cyclic adenosine monophosphate involved in signal transduction and metabolic processes. The lead SNPs for NDA rs6718628 and PD rs11676272 were also significant eQTLs associated with increased *ADCY3* expression in subcutaneous and visceral fat, and whole blood. On 15q25.2, the lead NDA SNP rs1812707 was strongly correlated (*r*^*2*^ = 0.88) with the V661L missense mutation variant in *ADAMTSL3*, involved in protein glycosylation and catabolism, and was also a significant eQTL in fibroblasts and subcutaneous fat for the *GOLGA6L5P* pseudogene, about which little is known.

On 19q13.33, the lead SNPs for DA rs492602 (*r*^*2*^ = 0.99) and PD rs1704773 (*r*^*2*^ = 0.89) were tightly linked with the W154X nonsense mutation in *FUT2* (fucosyltransferase 2) resulting in a truncated protein. *FUT2* is involved in the production of histo-blood group antigens, and exhibits the non-secretor phenotype (lack of antigens in epithelial mucosa and exocrine secretions) when inactivating mutations are present. The lead DA and PD SNPs were also significantly associated with decreased expression of *FUT2* in mammary tissue and fibroblasts, and increased expression in fibroblasts of the nearby *MAMSTR* gene, encoding a transcriptional regulator. This regulatory activity may be mediated in part by strong correlation (*r*^*2*^ ≥ 0.80) with an intronic variant of *MAMSTR* within a promoter and enhancer-like region in mammary epithelial cells and mammary fibroblasts (Supplementary Table 4).

The lead MD SNPs at 13 loci were significantly associated (FDR < 0.05) with gene expression levels in normal human mammary tissue, primary fibroblast cells, subcutaneous fat, visceral fat, or whole blood, which were the tissues most closely related to cell types in the breast available in GTEx^22,23^ (Supplementary Table 3). Target genes regulated by new DA SNPs at 4 loci (2p13.1, 10p12.1, 19q13.33, 20q13.13) included *FNBP1P1, FUT2, MAMSTR, MKX, NTN5, RASIP1, SEC1P*, and *SMIM25*. Target genes regulated by new NDA SNPs at 7 loci (1p12(2), 2p23.3, 3p14.1, 11p15.2, 11q24.3, 12q22, 15q25.2) included *ADAMTS8, ADAMTS9-AS2, ADCY3, ARNTL, CENPO, DNAJC27, GOLGA6L5P, NCOA1, NTN4*, and *WARS2*. Target genes regulated by PD SNPs at 5 loci overlapped with those for DA (19q13.33) or NDA (2p23.3, 11q24.3), except for *DUSP4* (8p12) and *LINC00445* (13q13.3), which were regulated by PD SNPs only.

MD SNPs at 13 loci, or strongly correlated (*r*^*2*^ ≥ 0.80) variants nearby, were located within regions with promoter or enhancer activity in normal human mammary epithelial cells or mammary fibroblasts using data from ENCODE^24,25^ (Supplementary Table 4). On 10q21.2, the new NDA SNP in an intron of *ZNF365* was perfectly linked (*r*^*2*^ = 1.0) with an enhancer-region variant in mammary fibroblasts. *ZNF365* is involved in regulating neuronal growth and DNA repair. On 14q24.1 the lead DA SNP was an intronic variant in *RAD51B* located within an enhancer-like region in mammary fibroblasts. The RAD51 protein family is essential for DNA repair by homologous recombination, and interacts with the major breast and ovarian cancer susceptibility genes *BRCA1* and *BRCA2*. On 18q21.33 the lead DA SNP was an intronic variant in *BCL2* located within an enhancer-like region in mammary fibroblasts. BCL2 suppresses apoptosis and constitutive expression is thought to cause follicular lymphoma.

### Enrichment of MD loci in fibroblast regulatory regions

To identify cell types through which DA, NDA, and PD loci influence their respective phenotypes, we tested for the enrichment of all independent prior or new loci for each MD phenotype in the regulatory regions of 125 diverse human cell and tissue types using the Uncovering Enrichment through Simulation (UES) method^26^ (Supplementary Table 9). Regulatory regions were defined by DNase I hypersensitive sites sequencing (DNase-seq) experiments available from the ENCODE and Roadmap Epigenomic consortia^27^. We found that NDA loci (*n* = 17) were significantly enriched in the open chromatin regions of fibroblast cell lines derived from three different normal human tissues at the Bonferroni threshold of *P* < 0.0004 accounting for 125 tests. NDA loci were observed within regulatory regions more often than expected under the null distribution in fibroblasts isolated from the lung (3.6-fold; *P* < 0.0001), skin (3.8-fold; *P* = 0.0001), and heart (3.2-fold; *P* = 0.0003). Suggestive enrichment of NDA loci at *P* < 0.01 was also found in the regulatory regions of 13 additional fibroblast cell lines from different tissues, including mammary fibroblasts (2.7-fold; *P* = 0.0066). The regulatory activity of NDA loci in mammary fibroblasts was further supported by our functional analyses showing that 6 of 13 new NDA loci were associated with promoter or enhancer regions in mammary fibroblasts (Supplementary Table 4). DA (*n* = 28) and PD (*n* = 20) loci were not significantly enriched in the regulatory regions of any normal human cell types. However, suggestive evidence of enrichment (*P* < 0.01) was found specifically in 6 fibroblast cell lines for DA loci, and 2 fibroblast cell lines for PD loci, and not in any other normal human cell type (Supplementary Table 9).

### Biological pathways

We identified distinct biological pathways significantly enriched (FDR < 0.05) for DA, NDA, and PD loci using DAVID^28,29^ to perform gene set enrichment analyses of all prior and new genome-wide significant loci. We found that NDA loci (*n* = 17) were significantly enriched by over ninefold for genes involved in mammary gland development (Supplementary Table 11). Other NDA pathways were related to metabolism, cell differentiation, and reproduction. DA loci (*n* = 28) were significantly enriched for genes involved in reproduction, apoptosis, metabolism, and signaling (Supplementary Table 10). PD loci (*n* = 20) were significantly enriched for genes involved in anatomical structure development and metabolism (Supplementary Table 12). Additional pathways implicated by combining all MD loci (*n* = 46) were involved in regulation of gene expression, nucleic acid binding and metabolism, and cell proliferation (Supplementary Data 1).

### Heritability

The proportion of phenotypic variance explained by all genotyped SNPs estimated using GCTA^30,31^ was 0.30 (SE = 0.02), 0.34 (SE = 0.02), and 0.31 (SE = 0.02) for DA, NDA, and PD, respectively. These results were comparable to a previous GWAS that reported estimates of 0.31 (SE = 0.07), 0.25 (SE = 0.07), and 0.29 (SE = 0.07) for absolute dense volume, nondense volume, and percent dense volume, respectively^10^. SNP-based heritability estimates represent the upper bound on the total proportion of phenotypic variance that could be explained by GWAS of common variants, and the lower bound for narrow-sense heritability estimated from twin studies because they do not account for rare variants that are not in linkage disequilibrium with the genotyped SNPs^32^. Heritability estimates from twin studies can also be influenced by nonadditive genetic effects and shared environmental effects^32^. Altogether, the newly identified and previously known MD loci explained 12.3%, 9.1%, and 8.7% of the SNP-based heritability for DA, NDA, and PD, respectively, compared with 3.3%, 2.1%, and 3.2% explained by previously known MD loci.

## Discussion

High MD is one of the strongest and most common risk factors for breast cancer, and has been estimated to account for up to one-third of all breast cancers^4^. PD, DA, and NDA are all highly heritable and significantly associated with breast cancer risk in observational studies^6^. However, the biological bases for how these breast tissue phenotypes are related to breast cancer development are poorly understood. In this GWAS meta-analysis, we identified 31 loci for MD phenotypes, tripling the total number of genome-wide significant loci from 15 previously to 46 presently. Seventeen of the MD loci also were associated with breast cancer risk in an independent large meta-analysis, identifying potential new breast cancer susceptibility alleles at seven loci. Mendelian randomization and genetic correlation analyses provided further evidence of the shared genetic etiology of all three MD phenotypes with breast cancer.

Among the seven loci newly associated with both MD and breast cancer risk, one locus on 3p25.2 was significantly (*P* < 5 × 10^−8^) associated with all three MD phenotypes. Intronic variants in *PPARG* (peroxisome proliferator activated receptor gamma) were associated with DA, PD, and breast cancer risk in the same direction, and with NDA and breast cancer risk in the opposite direction, as expected. *PPARG* is a member of the nuclear receptor family of ligand-activated transcription factors, and a regulator of adipocyte differentiation^33^. *PPARG* has been shown to inhibit transcription of aromatase, the rate-limiting enzyme in estrogen biosynthesis, in primary breast adipocytes^34^. Higher aromatase expression has been observed in dense breast tissue^35^, and treatment with aromatase inhibitors lowers breast cancer risk although associations with MD changes have been less consistent^36^. Estrogen may influence breast cancer risk as well as breast tissue composition through its proliferative effects on mammary cells^37^. *PPARG* is therefore plausibly involved in the development of DA, NDA, and PD, as well as breast cancer.

Five additional loci were newly associated with both DA and breast cancer risk in the same direction, identifying *LMNB1, MKX, PRKG1, MRPL17*, and *SMIM25* as candidate genes for both DA and breast cancer risk. On 5q23.2, rs6885843 was positively associated with DA and breast cancer risk, and located 44 kb upstream of *LMNB1* (lamin B1) involved in autosomal dominant adult-onset leukodystrophy^33^. On 10p12.1, rs2642278 was positively associated with DA and breast cancer risk, and an eQTL for *MKX* (mohawk homeobox), which plays a role in cell adhesion^33^. On 10q21.1, rs1892368 is an intronic variant in *PRKG1* (protein kinase cGMP-dependent 1) that was inversely associated with both DA and breast cancer risk (*P* = 4.8 × 10^−7^). *PRKG1* is a key mediator of the nitric oxide/cGMP signaling pathway important in many signal transduction processes^33^. On 11p15.4, rs11040963 was inversely associated with both DA and breast cancer risk (*P* = 4.5 × 10^−4^), and located 8 kb upstream of *MRPL17* (mitochondrial ribosomal protein L17) involved in protein synthesis in mitochondria^33^. On 20q13.13, rs17196752 was positively associated with DA and breast cancer risk, and uncorrelated (*r*^*2*^ = 0.0001) with the closest known breast cancer risk allele rs6122906^15^. rs17196752 is an intronic eQTL within an enhancer-like region in mammary fibroblasts that down-regulated the mammary tissue expression of *SMIM25* (Small Integral Membrane Protein 25). While little is known about *SMIM25* function, rs17196752 has been associated with white blood cell traits that have been linked to cancer and other systemic diseases^38^.

Finally, on 19q13.33, SNPs for DA (rs492602) and PD (rs1704773) were tightly linked with a *FUT2* protein-truncating mutation associated with the nonsecretor phenotype for histo-blood group antigens. Both SNPs also were associated with increased expression of the *MAMSTR* transcriptional regulator in fibroblasts, and a *MAMSTR* regulatory region in mammary epithelial cells and mammary fibroblasts. rs492602 has been associated with serum lipid levels^39^ but not breast cancer, and rs1704773 was associated with PD and breast cancer (*P* < 0.05) in opposite directions, indicating that *FUT2* and *MAMSTR* are candidate genes for DA and PD but may not be directly associated with breast cancer risk.

Among the ten new MD loci associated with known breast cancer susceptibility alleles, all but one were associated with MD phenotypes and breast cancer risk in consistent directions. SNPs on 2p24.1 were associated with both DA and PD in directions opposite to their known associations with breast cancer risk^15^. The DA SNP rs11684853 was tightly linked (*r*^*2*^ = 0.99) with variants in an enhancer element with strong activity in mammary epithelial cells and mammary fibroblasts. The PD SNP rs34331777 was about 100 kb away from the tumor suppressor gene *OSR1* (Odd-skipped related 1) encoding a zinc-finger transcription factor that acts on the p53 and Wnt/β-catenin signaling pathways^40^, and the *MIR4757* microRNA that could influence the translation of multiple target mRNAs with different effects on MD and breast cancer risk.

There are several potential biological mechanisms through which higher DA and PD may increase breast cancer risk. Dense areas of the breast contain more epithelial cells, fibroblasts, and collagen than nondense areas that contain more adipocytes^41^. Increased collagen alignment and stiffness of the extracellular matrix (ECM), associated with dense breast tissue, have been shown to induce malignant phenotypes in normal mammary epithelial cells^42^. Fibroblasts produce collagen and other ECM components, as well as proteases involved in ECM remodeling. In addition, fibroblast signaling is a critical determinant of normal mammary epithelial and adipocyte cell development and differentiation^43^, and cancer-associated fibroblasts can stimulate breast tumor progression^44^. Our finding that loci for all three MD phenotypes were enriched in the regulatory regions of fibroblasts more than in any other cell type is consistent with a key role of fibroblasts in regulating the stromal environment and normal breast tissue composition, as well as aberrant growth in breast tumors.

Mammary epithelial cells from high density tissues also have been shown to have greater DNA damage response signaling and shorter telomeres compared with mammary epithelial cells from low density tissues^45^. DNA damage may increase DA and PD, and decrease NDA, by repressing CD36 expression in mammary fibroblasts, which induces increased ECM deposition and decreased lipid storage in nonmalignant breast tissue^44^. CD36 is a widely expressed glycoprotein receptor that binds to a broad range of ligands, including ECM proteins and lipids, and modulates adipocyte differentiation, lipid metabolism, angiogenesis, apoptosis, cell-ECM interactions, and immune signaling^44,45^. Importantly, CD36 expression is primarily controlled by the PPARG transcription factor^45^, which we found to be associated with all three MD phenotypes and breast cancer risk. The etiologic role of DNA repair and apoptosis genes in dense breast tissue is further supported by our findings that variants in the *RAD51B* (14q24.1) DNA repair gene, and *FAF1* (1p32.3) and *BCL2* (18q21.33) apoptosis genes are significantly associated with DA, and that DA loci are enriched for genes in the apoptosis pathway.

NDA has been inversely associated with breast cancer risk independently of DA in observational studies^5,6^, but the etiologic nature of this association and underlying mechanisms are uncertain. Our findings demonstrating a significant inverse association of genetically estimated NDA and breast cancer risk in both Mendelian randomization and genetic correlation analyses provide strong evidence that this association is caused by shared underlying genetic and biological pathways. A limitation of this study is that relatively few women were diagnosed with breast cancer following the mammogram, precluding mediation analyses of the extent to which SNP associations with breast cancer risk are explained by MD phenotypes. A limitation of Mendelian randomization studies generally is the potential for bias due to horizontal pleiotropy, although the weighted median method is a relatively robust and statistically powerful approach^18,19^. While NDA and BMI effects are difficult to disentangle, the adipose tissues within the breast may play a more direct role in breast cancer etiology than distant adipose tissues. Mammary adipocytes secrete adipokines that modulate the stromal environment, and constitute a local source of lipids and metabolites that influence mammary epithelial cell growth and function^46^. In vivo models have shown that the mammary adipose environment is critical for mammary gland growth and development^46^.

The finding that NDA loci, but not DA or PD loci, are significantly enriched for genes involved in mammary gland development supports a key role of the nondense fatty tissues in breast health. Three of the four new NDA loci enriched for genes involved in mammary gland development were associated with NDA and breast cancer risk^15^ in opposite directions, implicating *GLI2*, *NCOA1,* and *NTN4* as candidate genes for both NDA and breast cancer. *GLI2* at 2q14.2 encodes a zinc finger transcription factor that mediates hedgehog signaling^33^. *NCOA1* expression was upregulated in mammary tissue by the NDA SNP at 2p23.3, and encodes a transcriptional coactivator for steroid and nuclear hormone receptors that stimulates transcriptional activity in a hormone-dependent fashion^33^. *NTN4* expression was upregulated in mammary tissue by the NDA SNP at 12q22, and encodes a member of the netrin protein family involved in neuronal growth, angiogenesis, and tumorigenesis^33^. Netrin 4 has been implicated in controlling epithelial cell branching morphogenesis in the breast^47^. These findings provide insights into the genetic basis for the inverse association of NDA with breast cancer risk.

In summary, this GWAS of 24,192 women from two independent population-based cohorts screened using Hologic or GE digital mammography, and MD phenotypes measured centrally using Cumulus, identified 31 MD loci, and new candidate genes for MD and breast cancer risk. The study findings support the etiologic role of NDA as well as DA and PD as modifiable risk factors for breast cancer that provide potential for intervention. With this study, all 46 genome-wide significant loci identified to date explain 12.3%, 9.1%, and 8.7% of the SNP-based heritability for DA, NDA, and PD. Future studies are needed to discover additional MD loci and to elucidate the different roles that the fatty and dense breast tissue components play in breast health and cancer risk.

## Methods

### Study design

We conducted a GWAS meta-analysis within the Research Program on Genes, Environment and Health (RPGEH) administered by Kaiser Permanente Northern California (KPNC) Division of Research^48,49^. RPGEH is population-based and participants were not selected based on any disease phenotype. All participants completed a health survey, and over 100,000 individuals provided a DNA sample that was genotyped genome-wide; this sample constitutes the Genetic Epidemiology Research on Adult Health and Aging (GERA) study^49^. Written informed consent was obtained from all participants. Institutional Review Board approvals for this study were obtained from KPNC, Stanford University, and the Icahn School of Medicine at Mount Sinai.

Hologic study: The first GWAS included 20,311 non-Hispanic white women who underwent bilateral screening mammography at age 39–80 years during 2004–2013 at one of 36 KPNC clinics using Hologic FFDM machines. Processed (for presentation) images were retrieved from the KPNC imaging archive. Hologic images were downsampled from a pixel size of 70 microns to 200 microns. The resulting image resolution exceeded that of the computer monitors used to view the images, and was therefore unlikely to influence the density measurements. A median filter with a 3-pixel radius was applied to the downsampled Hologic images to reduce digital noise and make the images appear more like screen film mammograms, which we found to improve the reproducibility of density measurements^50^. Hologic images were randomly assembled into 23 batches of up to 1100 images each, including randomly selected replicates for quality control.

GE study: The second GWAS included an independent sample of 3881 non-Hispanic white women who underwent bilateral screening mammography at age 38–77 years during 2004–2013 at one of 11 KPNC clinics using GE FFDM machines. GE images processed using Tissue Equalization software were retrieved from the KPNC imaging archive, and downsampled from a pixel size of 94 microns to 200 microns. We found that denoising of the GE images did not improve the reproducibility of the density measurements and therefore did not denoise the downsampled images^51^. GE images were randomly assembled into 6 batches of up to 700 images each, including randomly selected replicates for quality control.

### Density assessments

Women were excluded if their mammograms contained breast implants (3.6%), did not contain the entire breast (1%), or were unreadable/unavailable (2.6%)^51^. Women with a history of bilateral breast cancer (0.06%) were also excluded. For women with a history of unilateral breast cancer, we measured MD phenotypes using the contralateral (unaffected) breast image from the closest prediagnostic exam following the RPGEH survey when available, or prior to the survey otherwise^50^. For women with no history of breast cancer, we used the left breast image from the closest post-survey exam, except for a random 10% subset of women for whom the right breast image was used to blind the reader to cancer history. All density measurements were performed using a single craniocaudal view.

MD phenotypes were measured centrally using Cumulus6^16^ (provided by M.J.Y.), a computer-assisted method that requires the reader to select the pixel intensity threshold for distinguishing the dense and nondense portions of the breast image. The reader must also define the pectoral muscle boundary, whereas Cumulus6 detects the outer edge of the breast automatically for most FFDM images. Cumulus computes the PD as the DA divided by the total breast area. The NDA is equal to the total area minus the DA. All Cumulus measurements were performed by a single radiological technologist (R.Y.L.) trained by the software developer (M.J.Y.) and a breast imaging specialist (J.A.L.), who was certified in the Cumulus method and provided the gold standard measurements used for training and longitudinal evaluation.

In the first study, 23 batches of up to 1100 Hologic images were read consecutively over a period of eight months. Each batch contained 10% quality control images, including random replicates used to assess reader reproducibility and images with gold standard measurements by JAL used for calibration and periodic retraining. The batch-adjusted Pearson R for PD, DA, and NDA were: 0.952, 0.925, and 0.996 in the Hologic cohort. Following the completion of all Hologic density assessments, the reader underwent a training period to attain high reproducibility on GE images. Then, in the second study, 6 batches of up to 700 GE images were read consecutively over a period of 3 months. The batch-adjusted Pearson R for PD, DA, and NDA were: 0.961, 0.941, and 0.995 in the GE cohort. When multiple measurements were obtained per image, the mean values were used in subsequent analyses.

### Genotyping, quality control, and imputation

Over 650,000 SNPs were genotyped at the UCSF Institute for Human Genetics, Genomics Core Facility using a custom Affymetrix array optimized for individuals of European ancestry^48,52^. This array is estimated to report on 93% of common variants with minor allele frequency (MAF) > 0.05, and 73% of less common variants (MAF between 0.01 and 0.05) at *r*^*2*^ > 0.80, based on the 1000 Genomes Project (http://1000genomes.org) European population. Arrays were processed using the Affymetrix Axiom reagent kit 1.0 (96.7%) or 2.0 (3.3%). Genotype quality control procedures have been described previously^49^. Samples were excluded if the genotyping call rate was <0.97, or if there was evidence of trisomy, monosomy, male or ambiguous sex (PLINK v1.07^53^ X chromosome F-statistic >0.2), or excess heterozygosity (PLINK^53^ F-statistic <−0.03). Among first-degree female relatives (457 pairs and 14 trios), only the youngest woman was retained for analysis. Principal components of ancestry were computed for the genotyped SNPs using EIGENSOFT4.2^54^, and women were excluded if their principal components were not consistent with European ancestry^48^. Over 30 million variants were imputed from the 1000 Genomes Project reference panel using IMPUTE2.2.2^55–57^, after pre-phasing the genotyped SNPs using SHAPEIT v2.r644^58^. After excluding variants with MAF < 0.01 or imputation *r*^*2*^ ≤ 0.3^59^, there remained 9,906,178 variants available for analysis.

### GWAS meta-analysis

The MD phenotypes for each study cohort were transformed separately to attain standard normal distributions with mean 0 and variance 1, to facilitate estimation of the combined meta-analytic effects and enable interpretation of effect sizes in SD units. The distributions of MD phenotypes differed between women in the two cohorts and required different transformations (Supplementary Fig. 1). The optimal power transformations determined using the R boxcox package for DA, NDA, and PD, respectively, were: fifth-root, cube-root, and cube-root for the Hologic cohort; and cube-root, cube-root, and square-root for the GE cohort. For computational efficiency, each phenotype was pre-adjusted for image batch using linear regression, and the residuals were used in GWAS analyses.

Separate GWAS analyses of each standardized MD phenotype in the Hologic cohort (*n* = 20,311) and GE cohort (*n* = 3881) were performed with PLINK^53^ using linear regression models of each SNP as an additive dosage effect^60^, adjusted for ln(BMI), age at mammography, genotyping reagent kit, and the first ten principal components of European ancestry^48,49^. BMI was determined from electronic health records for the patient visit closest to the date of mammography. There was a linear relationship of age (Supplementary Fig. 6) and ln(BMI) with the normalized density phenotypes, except in the extreme tails of the BMI distribution where the data were sparse (Supplementary Fig. 7). GWAS meta-analyses were conducted using an inverse-variance weighted fixed-effects model implemented in METAL^61^. To be considered statistically significant, we required SNPs to: meet the conventional genome-wide significance threshold of *P* < 5 × 10^−8^ in the meta-analysis of the Hologic and GE studies combined; and have the same direction of association in both studies. A single genotyped SNP rs3819405 on 6p22.3 that was significantly associated with DA and PD had low levels of LD with all nearby SNPs (Supplementary Figs. 3H and 5F); rs3819405 had a high call rate of 99.89%, and the MAF of 0.33 was similar to the MAF of 0.34 among individuals of European ancestry in the 1000 Genomes Project.

Conditionally independent SNPs within the same chromosomal region were identified by conditional analyses adjusting for the lead SNP. To be considered statistically significant, both SNPs were required to have: meta-analytic *P* < 5 × 10^−8^ in the conditional analysis; the same direction of associations in both studies; and low linkage disequilibrium (LD; *r*^*2*^ < 0.10). Novel loci were identified by conditional analyses adjusting for the nearest known SNP for any MD phenotype. To be considered novel, SNPs were required to meet the genome-wide significance threshold of *P* < 5 × 10^−8^ in conditional analyses adjusting for the nearest known MD SNP, and to have low LD (*r*^*2*^ < 0.10) with previously reported genome-wide significant SNPs for any MD phenotype on the same chromosome.

Quantile-quantile plots and genomic inflation factors^62^ were used to assess the presence of inflated significance levels due to uncontrolled population substructure. LocusZoom v1.3^63^ plots of the 400 kb region centered around each novel lead SNP were used to visualize the GWAS meta-analysis significance levels, linkage disequilibrium with the lead SNP, local recombination rates from HapMap, and nearby genes.

### Associations with breast cancer

We evaluated associations of newly identified MD SNPs with breast cancer risk in 122,977 cases and 105,974 controls of European ancestry from the BCAC and Discovery, Biology and Risk of Inherited Variants in Breast Cancer Consortium (DRIVE)^15^. Associations of MD SNPs with risk of estrogen receptor (ER)-negative breast cancer were evaluated in a subset of 21,468 cases and 100,594 controls^15,64^. Summary statistics were obtained from: http://bcac.ccge.medschl.cam.ac.uk/bcacdata/. MD SNPs were considered to be potentially novel breast cancer loci if they were associated with breast cancer with *P* < 0.05 and *P* > 5 × 10^−8^ in the BCAC/DRIVE data, and were uncorrelated (*r*^*2*^ ≤ 0.01) with previously reported genome-wide significant breast cancer SNP in the NHGRI-EBI GWAS catalog^65^ (https://www.ebi.ac.uk/gwas/).

### Mendelian randomization and genetic correlation of MD and breast cancer

Mendelian randomization analyses were performed to evaluate the potential causal associations of MD phenotypes with breast cancer risk. We used the weighted median method to estimate the causal effect because it is more efficient and robust to violations of instrumental variable assumptions than other Mendelian randomization methods for summary statistics, and provides consistent estimates even when up to half of the information comes from invalid instrumental variables^18^. We considered the first reported SNP at all independent prior and new genome-wide significant loci (Table 1 and Supplementary Table 7) for DA (*n* = 28), NDA (*n* = 17), and PD (*n* = 20), and estimated their associations with the relevant MD phenotype in the GWAS meta-analysis. Summary statistics for SNP associations with breast cancer in 122,977 cases and 105,974 controls of European ancestry were obtained from BCAC/DRIVE^15^.

LD Score regression was performed to estimate the genetic correlation between MD phenotypes and breast cancer from GWAS summary statistics using the LDSC v1.0.1 software^20,21^. We used the LD scores for the European ancestry population from the 1000 Genomes Project provided by the software developers^20,21^. A total of 779,828 SNPs were included that had available LD scores and summary statistics from this MD GWAS and the BCAC/DRIVE breast cancer GWAS^15^.

### Regulatory function of MD SNPs

We evaluated whether MD SNPs were associated with gene expression levels in: human mammary tissue (*n* = 251), primary fibroblast cells (*n* = 300), subcutaneous fat (*n* = 385), visceral fat (*n* = 313), and whole blood cells (*n* = 369) using data from the Genome-Tissue Expression (GTEx) project version 7 (https://gtexportal.org/)^22,23^. Significant *cis*-eQTLs within 1 Mb of the gene transcription start site were identified by computing q-values for SNP-gene pairs involving one of the lead MD SNPs using the R qvalue package, and controlling for a false discovery rate (FDR) of 0.05. We also assessed whether lead MD SNPs or nearby proxies (*r*^*2*^ ≥ 0.80 in Europeans from the 1000 Genomes Project) were located within promoter or enhancer regions in human primary mammary epithelial cells and human primary mammary fibroblasts using data from the ENCODE and Roadmap Epigenomic consortia (https://www.encodeproject.org/)^24,25^. Promoter-like regions were identified by combining DNase hypersensitivity and histone modification H3K4me3 signals in the same cell type^24,25^. Enhancer-like regions were identified based on DNase hypersensitivity and histone modification H3K27ac signals in mammary epithelial cells, and DNAse hypersensitivity only in mammary fibroblasts^24,25^.

### Tissue enrichment of MD SNPs in regulatory regions

We tested whether previously identified and new loci for each MD phenotype were enriched in regulatory regions in 125 diverse human cell and tissue types using the UES method (https://github.com/robertkleinlab/uesEnrichment)^26^. Open chromatin regions were defined using DNase I hypersensitive sites sequencing data from the ENCODE and Roadmap Epigenomic consortia^27^. The empirical *P* value for the observed enrichment of independent MD SNPs in the regulatory regions of each cell line was computed by generating 10,000 sets of randomly selected SNPs that were matched to MD SNPs by the distance to the nearest transcription start site and number of correlated SNPs. We applied a Bonferroni correction for the 125 cell lines tested to determine the significance threshold of *P* < 0.0004.

### Pathway analysis

We performed gene set enrichment analyses to identify biological pathways implicated by prior and new MD loci using DAVID v6.8^28,29^. For each MD locus, we included the nearest flanking protein-coding genes within 500 kb of the lead SNP, as well as target genes whose expression levels were associated with the lead MD SNP (*cis*-eQTL) in mammary tissue, primary fibroblast cells, subcutaneous fat, visceral fat, or whole blood (Table 1 and Supplementary Tables 3, 7, and 8). We estimated the fold enrichment of MD genes among gene sets or pathways with FDR < 0.05 from the Gene Ontology, KEGG, Reactome, and Biocarta databases, and identified the responsible genes using DAVID^28,29^.

### Heritability

We estimated the proportion of phenotypic variance explained by the additive genetic effects of all genotyped SNPs using GCTA v1.02^31^. Array-based heritability was estimated separately for the Hologic and GE study cohorts, and the resulting estimates were then combined using inverse-variance weighting. We also estimated the proportion of phenotypic variance explained by all independent prior and new genome-wide significant MD loci. For each MD phenotype, the residual variance of linear regression models were estimated using 100-fold cross-validation, where model 1 included all independent genome-wide significant loci in addition to the adjustment variables in the GWAS model, and model 2 included only the non-SNP covariates. In both models, the SNP effects and covariates were nested within the Hologic or GE studies to account for differences across studies. The proportion of variance explained by the genome-wide significant loci for each MD phenotype was computed by 1 − *V1/V2*, where *V1* and *V2* represent the estimated residual variances of models 1 and 2 respectively.

### Reporting summary

Further information on research design is available in the Nature Research Reporting Summary linked to this article.

## Supplementary information

Supplementary Information

Description of Additional Supplementary Files

Supplementary Data 1

Reporting Summary

## Data Availability

Genotype data of RPGEH GERA participants are available from the database of Genotypes and Phenotypes (dbGaP) under accession phs000674.v3.p3. This includes individuals who consented to having their data shared with dbGaP. The complete GERA data are available upon application to the KP Research Bank (https://researchbank.kaiserpermanente.org/our-research/for-researchers). Breast cancer summary statistics are available at http://bcac.ccge.medschl.cam.ac.uk/bcacdata/. All remaining relevant data are available in the article, Supplementary Information, or from the corresponding author upon reasonable request.
