# Supplementary material for: Identification of 31 loci for mammographic density phenotypes and their associations with breast cancer risk
Source: Nat Commun. 2020 Oct 9;11:5116. doi: 10.1038/s41467-020-18883-x (PMC7547012; doi:10.1038/s41467-020-18883-x)
Supplement: Supplementary file 1 — Supplementary Information [file 41467_2020_18883_MOESM1_ESM.pdf]

Identification of 31 Loci for Mammographic Density Phenotypes and their Associations with Breast Cancer Risk

Sieh, W. *et al*.

SUPPLEMENTARY TABLES

Supplementary Table 1. Characteristics of women included in Stage 1 and Stage 2.

Supplementary Table 2. Nonsynonymous variants in high linkage disequilibrium ($r^2 \geq 0.8$) with lead density SNPs.

Supplementary Table 3. Genes regulated by lead density SNPs that were significant eQTLs in five biologically relevant human tissues.

Supplementary Table 4. Promoter and enhancer-like regions in normal human mammary epithelial cells or mammary fibroblasts associated with lead density SNPs or nearby variants ($r^2 \geq 0.8$).

Supplementary Table 5. Association of 39 novel MD alleles in 31 independent regions with DA, NDA, and PD.

Supplementary Table 6. Association of 39 novel MD alleles in 31 independent regions with breast cancer (all, ER-positive, and ER-negative).

Supplementary Table 7. Association of previously reported MD loci with standardized MD phenotypes.

Supplementary Table 8. Genes regulated by lead SNPs at previously reported MD loci that were significant eQTLs in mammary tissue, fibroblasts, fatty tissues, or blood.

Supplementary Table 9. Cell-specific enrichment of MD loci within gene regulatory regions.

Supplementary Table 10. Gene set enrichment analysis of candidate genes for DA.

Supplementary Table 11. Gene set enrichment analysis of candidate genes for NDA.

Supplementary Table 12. Gene set enrichment analysis of candidate genes for PD.

Supplementary Table 13. Sensitivity of NDA loci to further adjustment for BMI.

Supplementary Table 14. Sensitivity analyses of Mendelian randomization estimates for the association of MD phenotypes with breast cancer risk.

Supplementary Table 15. Association of novel alleles with MD phenotypes, stratified by menopausal status.

SUPPLEMENTARY FIGURES

Supplementary Figure 1. Distribution of dense area, nondense area and percent density measured from Hologic and GE mammograms before (A) and after (B) transformation to standard normal distributions.

Supplementary Figure 2. Quantile-quantile plots of the GWAS meta-analysis results for dense

area, non-dense area, and percent density.

Supplementary Figure 3. Regional association plots for 16 loci (A-P) associated with dense area.

Supplementary Figure 4. Regional association plots for 13 loci (A-M) associated with nondense area.

Supplementary Figure 5. Regional association plots for 12 loci (A-L) associated with percent density.

Supplementary Figure 6. Distribution of age at mammography (A), and its relationship with standardized measures of dense area, nondense area and percent density from Hologic and GE mammograms (B).

Supplementary Figure 7. Distribution of the natural logarithm (ln) of BMI (A), and its relationship with standardized measures of dense area, nondense area and percent density from Hologic and GE mammograms (B).

Supplementary Table 1. Characteristics of women included in Stage 1 and Stage 2.

Characteristic	Stage 1 (N = 20,311)	Stage 2 (N = 3,881)
Mammography machine manufacturer	Hologic	GE
Year of mammography	2004-2013	2004-2013
Age at mammography, mean ± SD	61.9 ± 8.6	59.2 ± 8.9
Body mass index (kg/m^2), mean ± SD	27.7 ± 6.2	26.9 ± 5.8
Post-menopausal, %	81	77.2
Unilateral breast cancer[a], n (%)	1624 (8.0%)	346 (9.1%)
Pre-diagnostic mammogram available	468	138
Mammographic density phenotypes, mean ± SD		
Dense area (cm^2)	27.9 ± 17.9	29.0 ± 20.7
Non-dense area (cm^2)	140.2 ± 77.7	109.0 ± 60.8
Percent density (%)	20.4 ± 14.9	24.4 ± 17.1
Correlation of standardized MD phenotypes, Pearson *R*		
Dense area and non-dense area	-0.35	-0.35
Dense area and percent density	0.82	0.84
Non-dense area and percent density	-0.81	-0.78

a. MD phenotypes were measured from the contralateral unaffected breast for women with unilateral breast cancer, and pre-diagnostic mammograms were used when available.

Supplementary Table 2. Nonsynonymous variants in high linkage disequilibrium ($r^2 \geq 0.8$) with lead density SNPs.

Region	Lead density SNP (phenotype)	Coding variant	Position (hg19)	Ref/Alt	Gene	Mutation[a]	NCBI reference sequence: exon: coding DNA: protein sequence
2p23.3	rs6718628 (NDA), rs11676272 (PD)	Lead SNP (PD)	25141538	A/G	ADCY3	NS	NM_004036: exon 1: c.T319C: p.S107P
15q25.2	rs1812707 (NDA)	rs4842838	84582124	G/T	ADAMTSL3	NS	NM_001301110: exon 16: c.G1981T: p.V661L, NM_207517: exon 16: c.G1981T: p.V661L
19q13.33	rs492602 (DA), rs1704773 (PD)	rs601338	49206674	G/A	FUT2	Stop	NM_000511: exon 2: c.G461A: p.W154X, NM_001097638: exon 2: c.G461A: p.W154X
		rs602662	49206985	G/A	FUT2	NS	NM_000511: exon 2: c.G772A: p.G258S, NM_001097638: exon 2: c.G772A: p.G258S

a. Nonsynonymous (NS) or stop-gain (stop) mutations.

Supplementary Table 3. Genes regulated by lead density SNPs that were significant eQTLs[a] in five biologically relevant human tissues.

Region[b]	Lead density SNP (phenotype)	Position (hg19)	Ref/Alt	Mammary tissue	Fibroblast cells	Subcutaneous fat	Visceral fat	Whole blood
1p12(2)	rs1779445 (NDA)	119508412	T/C	WARS2 (−)	WARS2* (−)	RP4-712E4.1* (−), WARS2* (−), WARS2-AS1* (−)	WARS2* (−), WARS2-AS1 (−)	—
2p23.3	rs6718628 (NDA)	25131170	C/G	NCOA1 (+)	—	CENPO* (+), ADCY3* (+)	CENPO* (+), ADCY3* (+)	CENPO* (+), ADCY3* (+) DNAJC27* (+)
	rs11676272 (PD)	25141538	A/G	—	—	AC008073.2 (+), CENPO* (+), ADCY3* (+)	CENPO* (+), ADCY3* (+)	CENPO* (+), ADCY3* (+) DNAJC27* (+)
2p13.1	rs6715731 (DA)	74346404	G/T	FNBP1P1* (+), MGC10955* (+)	FNBP1P1* (+)	FNBP1P1* (+), MGC10955* (+)	FNBP1P1* (+), MGC10955* (+)	FNBP1P1 (+)
3p14.1	rs4132228 (NDA)	64708114	C/T	—	—	—	ADAMTS9-AS2 (+)	
8p12	rs6558136 (PD)	29523123	C/T	—	DUSP4* (−)	—	—	DUSP4 (+), RP11-486M23.2 (−)
10p12.1	rs2642278 (DA)	27936562	T/C	—	MKX* (+)	—	—	
11p15.2[c]	rs60521023 (NDA)	13314102	A/AT	—	—	—	—	ARNTL* (−)
11q24.3	rs6590455 (PD)	130276427	T/C	—	—	—	ADAMTS8* (+)	—
	rs2875238 (NDA)	130282078	T/C	—	—	—	ADAMTS8 (+)	—
12q22	rs11836367 (NDA)	96027467	C/T	NTN4* (+)	—	—	—	—
13q13.3	rs10219975 (PD)	36269125	G/C	LINC00445 (+)	—	—	—	—
15q25.2	rs1812707 (NDA)	84547222	C/T	GOLGA2P7 (−)	GOLGA6L5P* (−)	DNM1P51 (−), GOLGA6L5P* (−)	GOLGA2P7 (−)	—
19q13.33	rs492602 (DA)	49206417	A/G	SEC1P (+), FUT2 (−)	NTN5* (+), FUT2* (−), MAMSTR (+), RASIP1* (+)	SEC1P (+)	—	NTN5 (+)
	rs1704773 (PD)	49209566	A/G	FUT2 (−)	NTN5* (+), FUT2* (−), MAMSTR (+), RASIP1* (+)	—	—	—
20q13.13	rs17196752 (DA)	48887268	C/T	SMIM25* (−)	SMIM25* (−)	SMIM25* (−)	SMIM25* (−)	LINC01270* (−)

a. Significant (FDR < 0.05) eQTLs identified by computing q-values for all SNP-gene pairs involving the lead density SNPs that were tested in mammary tissue, primary fibroblast cell culture, subcutaneous fat, visceral fat, or whole blood samples in GTEx V7; up- or down-regulation of gene expression associated with the alternate allele is indicated by (+) or (-), respectively, after the target gene name. Source: https://gtexportal.org/

b. Independent regions within the same cytoband are denoted by (1) or (2) in order by position.

c. rs12290622 was used as a proxy for rs60521023 (r^2 = 1).

* Genes with a significant eQTL identified by GTEx V7 analyses of all possible SNP-gene pairs genome-wide.

Supplementary Table 4. Promoter and enhancer-like regionsa in normal human mammary epithelial cells or mammary fibroblasts associated with lead density SNPs or nearby variants ($r^2 \geq 0.8$).

Region[b]	Lead density SNP (phenotype)	Functional variant	Position (hg19)	Gene[c]	Ref/Alt	Mammary epithelial cells	Mammary fibroblasts
1p12(2)	rs1779445 (NDA)	Lead SNP	119508412	TBX15	T/C	—	Enhancer
		rs4659137	119520794	TBX15	C/T	—	Enhancer
2p24.1[d]	rs11684853 (DA)	rs12710697	19320968	OSR1	C/T	Enhancer	Enhancer
		rs10206829	19321500	OSR1	G/A	Enhancer	Enhancer
4q28.1[e]	rs1503613 (DA)	Lead SNP	127772368	—	G/T	—	Enhancer
6p25.1	rs1294419 (NDA)	rs1294438	6752059	LY86, RREB1	C/T	—	Enhancer
8p12	rs6558136 (PD)	Lead SNP	29523123	DUSP4, SARAF	C/T	Enhancer	Enhancer
10q21.2(1)[f]	rs1949355 (NDA), rs2138555 (PD)	rs12220488	64219000	ZNF365	G/A	—	Enhancer
11p15.2	rs60521023 (NDA)	rs11022743	13297800	RASSF10, ARNTL	G/A	—	Enhancer
		rs2279284	13298750	RASSF10, ARNTL	C/T	Promoter, Enhancer	Promoter, Enhancer
12q22	rs11836367 (NDA)	Lead SNP	96027467	USP44, NTN4	C/T	Enhancer	Enhancer
		rs17356907	96027759	USP44, NTN4	A/G	Enhancer	Enhancer
14q24.1	rs75197674 (DA)	Lead SNP	68971452	RAD51B	T/C	—	Enhancer
15q25.2	rs1812707 (NDA)	rs7162542	84514290	ADAMTSL3	C/G	Enhancer	Enhancer
		rs7183263	84573041	ADAMTSL3	T/G	—	Enhancer
18q21.33	rs11877925 (DA)	Lead SNP	60896694	BCL2	G/A	—	Enhancer
19q13.33	rs1704773 (PD), rs492602 (DA)	rs35866622	49218060	MAMSTR	C/T	Promoter, Enhancer	Promoter, Enhancer
20q13.13	rs17196752 (DA)	Lead SNP	48887268	SMIM25	C/T	—	Enhancer

a. Source: https://www.encodeproject.org/data/annotations/v3/

b. Independent regions within the same cytoband are denoted by (1) or (2) in order by position.

c. Nearest flanking protein coding gene(s) within 500 kb of the functional variant.

d. LD was moderate between the lead SNPs for DA and PD (r2 = 0.53) at 2p24.1, and rs34331777 (PD) was not associated with an enhancer.

e. LD was low between the lead SNPs for DA and PD (r^2 = 0.19) at 4q28.1, and rs35589286 (PD) was not associated with an enhancer.

f. LD was low between the lead SNPs for DA and PD (r^2 = 0.07) or NDA (r^2 = 0.07) at 10q21.2, and rs10995181 (DA) not associated with an enhancer.

Supplementary Table 5. Association of 39 novel MD alleles in 31 independent regions with DA, NDA, and PD.

#	Region[a]	Lead SNP	Position	Imputation r^2	Gene[b]	Gene Region (ncRNA)	Ref/Alt	AAF	Associated MD Phenotype	Dense Area			Non-Dense Area			Percent Density		
										Beta	*SE*	*Combined P*	*Beta*	*SE*	*Combined P*	*Beta*	*SE*	*Combined P*
1	1p36.11	rs61777307	23959319	1.00	MDS2	intronic	G/A	0.24	NDA	0.028	0.010	6.0E-03	-0.042	0.007	3.5E-09	0.042	0.008	4.2E-07
2	1p32.3	rs6703250	51418472	0.95	FAF1	intronic	C/T	0.55	DA	-0.054	0.009	4.8E-10	0.009	0.006	1.3E-01	-0.039	0.007	1.1E-07
3	1p12(1)	rs10802015	118782659	0.95	SPAG17	intergenic	C/T	0.34	NDA	-0.002	0.009	8.7E-01	-0.041	0.007	2.1E-10	0.023	0.008	3.4E-03
4	1p12(2)	rs1779445	119508412	0.99	TBX15	intronic	T/C	0.80	NDA	-0.026	0.011	1.8E-02	0.052	0.008	6.2E-12	-0.046	0.009	2.9E-07
5	2p24.1	rs11684853	19310918	1.00	OSR1	intergenic	G/T	0.55	DA	0.049	0.009	2.5E-08	-0.016	0.006	8.8E-03	0.040	0.007	1.4E-08
		rs34331777	19441251	0.99		intergenic	CA/C	0.60	PD	0.046	0.009	1.1E-07	-0.023	0.006	2.5E-04	0.042	0.007	4.5E-09
6	2p23.3	rs6718628	25131170	0.94	ADCY3	intronic	C/G	0.40	NDA	-0.026	0.009	5.1E-03	0.041	0.006	1.1E-10	-0.042	0.008	2.3E-08
		rs11676272	25141538	0.98		exonic	A/G	0.47	PD	-0.032	0.009	2.5E-04	0.035	0.006	9.3E-09	-0.043	0.007	2.2E-09
7	2p13.1	rs6715731	74346404	0.99	TET3, BOLA3	intergenic	G/T	0.42	DA	-0.048	0.009	4.7E-08	0.012	0.006	4.6E-02	-0.037	0.007	6.0E-07
8	2q14.2(2)	rs11123556	121245996	1.00	INHBB, GLI2	intergenic	A/G	0.89	NDA	-0.021	0.014	1.2E-01	-0.075	0.010	9.2E-15	0.026	0.011	2.1E-02
9	2q35	rs66470855	218266438	0.99	TNS1	intronic (DIRC3)	TG/T	0.26	DA, PD	0.060	0.010	8.5E-10	-0.014	0.007	5.4E-02	0.046	0.008	2.0E-08
10	3p25.2	rs67901221	12416550	0.98	PPARG	intronic	A/G	0.15	DA	0.069	0.012	1.4E-08	-0.046	0.008	5.1E-08	0.068	0.010	7.4E-12
		rs199689761	12441088	0.94		intronic	T/TA	0.16	NDA	0.064	0.012	9.9E-08	-0.047	0.008	2.4E-08	0.066	0.010	3.4E-11
		rs76643909	12441367	0.96		intronic	T/G	0.16	PD	0.068	0.012	1.8E-08	-0.046	0.008	3.0E-08	0.069	0.010	4.9E-12
11	3p14.1	rs4132228	64708114	1.00	ADAMTS9	intronic (ADAMTS9-AS2)	C/T	0.31	NDA	0.016	0.009	8.2E-02	-0.048	0.007	2.3E-13	0.041	0.008	1.1E-07
12	4q28.1	rs1503613	127772368	1.00	—	intergenic	G/T	0.47	DA	0.054	0.009	6.1E-10	-0.006	0.006	3.0E-01	0.038	0.007	6.7E-08
		rs35589286	128192680	0.95	INTU	intergenic	G/C	0.29	PD	0.050	0.010	3.6E-07	-0.025	0.007	3.0E-04	0.045	0.008	2.0E-08
13	5q23.2(2)	rs6885843	126068500	1.00	TEX43, LMNB1	intergenic	C/T	0.40	DA	0.048	0.009	4.3E-08	-0.008	0.006	1.8E-01	0.036	0.007	1.1E-06
14	6p25.1	rs1294419	6742549	0.99	LY86, RREB1	intergenic	C/G	0.62	NDA	0.013	0.009	1.5E-01	0.038	0.006	8.3E-10	-0.013	0.007	6.8E-02
15	6p22.3	rs3819405	16399557	1.00	ATXN1	intronic	C/T	0.33	DA, PD	-0.069	0.009	4.4E-14	0.024	0.006	1.2E-04	-0.059	0.008	5.0E-15
16	8p12	rs6558136	29523123	0.98	DUSP4, SARAF	intergenic	C/T	0.65	PD	-0.046	0.009	3.6E-07	0.021	0.006	8.5E-04	-0.042	0.008	1.4E-08
17	10p12.1	rs2642278	27936562	1.00	RAB18, MKX	intergenic	T/C	0.36	DA	0.050	0.009	1.0E-08	0.002	0.006	7.7E-01	0.032	0.007	1.5E-05
18	10q21.1	rs1892368	53674795	1.00	PRKG1	intronic	G/A	0.67	DA	-0.051	0.009	2.4E-08	0.005	0.006	4.6E-01	-0.035	0.008	2.6E-06
19	10q21.2(1)	rs1949355	64218423	0.99	ZNF365	intronic	G/A	0.56	NDA	0.054	0.009	7.2E-10	-0.039	0.006	1.7E-10	0.056	0.007	6.1E-15
		rs2138555	64220494	1.00		intronic	A/G	0.41	PD	-0.058	0.009	2.8E-11	0.036	0.006	2.7E-09	-0.057	0.007	2.3E-15
20	11p15.4	rs11040963	6713214	0.99	MRPL17, OR2AG2	intergenic	C/A	0.28	DA	-0.053	0.010	2.1E-08	0.010	0.007	1.2E-01	-0.040	0.008	6.8E-07
21	11p15.2	rs60521023	13314102	0.96	ARNTL	intronic	A/AT	0.70	NDA	-0.023	0.010	1.5E-02	0.041	0.007	7.5E-10	-0.041	0.008	1.4E-07
22	11q24.3	rs6590455	130276427	0.99	ADAMTS8	intronic	T/C	0.65	PD	-0.030	0.009	1.0E-03	0.038	0.006	2.7E-09	-0.041	0.008	5.0E-08
		rs2875238	130282078	0.94		intronic	T/C	0.66	NDA	-0.026	0.009	6.7E-03	0.041	0.007	2.5E-10	-0.040	0.008	2.6E-07
23	12p12.1	rs1818476	26445564	0.99	SSPN, ITPR2	intergenic	C/T	0.75	DA	-0.055	0.010	2.4E-08	-0.004	0.007	5.9E-01	-0.035	0.008	2.8E-05
24	12q22	rs11836367	96027467	0.78	USP44, NTN4	intergenic	C/T	0.33	NDA	0.010	0.010	3.2E-01	0.042	0.007	8.4E-09	-0.018	0.009	4.1E-02
25	13q13.3	rs10219975	36269125	0.93	NBEA, DCLK1	intronic (LINC00445)	G/C	0.32	PD	-0.043	0.010	5.9E-06	0.030	0.007	6.3E-06	-0.045	0.008	1.4E-08
26	14q24.1	rs75197674	68971452	0.91	RAD51B	intronic	T/C	0.21	DA	-0.064	0.011	5.9E-09	0.008	0.008	3.2E-01	-0.046	0.009	6.1E-07
27	15q25.2	rs1812707	84547222	0.98	ADAMTSL3	intronic	C/T	0.50	NDA	-0.018	0.009	3.7E-02	0.034	0.006	3.1E-08	-0.031	0.007	3.2E-05
28	15q26.1	rs4777948	94298239	0.96	—	intergenic	G/T	0.47	PD	0.041	0.009	3.1E-06	-0.034	0.006	5.4E-08	0.046	0.007	2.8E-10
29	18q21.33	rs11877925	60896694	0.98	BCL2	intronic	G/A	0.22	DA	-0.060	0.010	7.4E-09	-0.001	0.007	8.7E-01	-0.038	0.009	9.8E-06
30	19q13.33	rs492602	49206417	1.00	FUT2	exonic	A/G	0.48	DA	-0.060	0.009	6.9E-12	0.006	0.006	3.3E-01	-0.041	0.007	1.7E-08
		rs1704773	49209566	0.95	FUT2, MAMSTR	downstream	A/G	0.49	PD	-0.062	0.009	1.0E-11	0.010	0.006	1.3E-01	-0.044	0.007	3.5E-09
31	20q13.13	rs17196752	48887268	0.95	SMIM25	intronic	C/T	0.20	DA	0.063	0.011	9.2E-09	0.012	0.008	1.3E-01	0.032	0.009	4.3E-04

a. Independent regions within the same cytoband are denoted by (1) or (2) in order by position.

b. Nearest flanking protein coding gene(s) within 500 kb of the lead SNP.

Supplementary Table 6. Association of 39 novel MD alleles in 31 independent regions with breast cancer (all, ER-positive, and ER-negative).

#	Region[a]	Lead SNP	Position	Gene[b]	Ref/Alt	AAF	Associated MD Phenotype	Breast Cancer			ER-Positive			ER-Negative		
								Beta	*SE*	*P-value*	*Beta*	*SE*	*P-value*	*Beta*	*SE*	*P-value*
1	1p36.11	rs61777307	23959319	MDS2	G/A	0.24	NDA	0.001	0.008	9.0E-01	0.004	0.009	7.0E-01	0.011	0.014	4.5E-01
2	1p32.3	rs6703250	51418472	FAF1	C/T	0.55	DA	-0.033	0.006	1.3E-07	-0.037	0.008	6.7E-07	-0.016	0.012	1.7E-01
3	1p12(1)	rs10802015	118782659	SPAG17	C/T	0.34	NDA	0.004	0.007	5.3E-01	0.009	0.008	2.7E-01	-0.006	0.012	6.3E-01
4	1p12(2)	rs1779445	119508412	TBX15	T/C	0.80	NDA	-0.013	0.008	1.0E-01	-0.018	0.010	7.0E-02	-0.008	0.015	6.0E-01
5	2p24.1	rs11684853	19310918	OSR1	G/T	0.55	DA	-0.044	0.006	1.1E-12	-0.027	0.007	2.8E-04	-0.066	0.011	6.5E-09
		rs34331777	19441251		CA/C	0.60	PD	-0.033	0.006	2.8E-07	-0.026	0.008	6.9E-04	-0.035	0.012	3.2E-03
6	2p23.3	rs6718628	25131170	ADCY3	C/G	0.40	NDA	-0.042	0.007	2.9E-10	-0.036	0.008	7.0E-06	-0.059	0.012	9.4E-07
		rs11676272	25141538		A/G	0.47	PD	-0.040	0.007	1.2E-09	-0.033	0.008	2.8E-05	-0.054	0.012	6.8E-06
7	2p13.1	rs6715731	74346404	TET3, BOLA3	G/T	0.42	DA	-0.011	0.007	1.1E-01	-0.012	0.008	1.4E-01	-0.002	0.013	8.8E-01
8	2q14.2(2)	rs11123556	121245996	INHBB, GLI2	A/G	0.89	NDA	0.095	0.010	5.3E-20	0.085	0.012	1.0E-11	0.115	0.019	2.9E-09
9	2q35	rs66470855	218266438	TNS1	TG/T	0.26	DA, PD	0.072	0.007	3.2E-24	0.071	0.008	9.7E-18	0.071	0.013	4.8E-08
10	3p25.2	rs67901221	12416550	PPARG	A/G	0.15	DA	0.019	0.009	3.1E-02	0.015	0.011	1.5E-01	0.028	0.016	7.9E-02
		rs199689761	12441088		T/TA	0.16	NDA	0.018	0.009	4.9E-02	0.015	0.011	1.7E-01	0.027	0.017	1.1E-01
		rs76643909	12441367		T/G	0.16	PD	0.018	0.009	4.2E-02	0.013	0.011	2.2E-01	0.031	0.016	5.3E-02
11	3p14.1	rs4132228	64708114	ADAMTS9	C/T	0.31	NDA	0.007	0.007	3.4E-01	0.004	0.008	6.1E-01	-0.003	0.012	7.8E-01
12	4q28.1	rs1503613	127772368	—	G/T	0.47	DA	0.008	0.006	2.2E-01	0.006	0.007	4.5E-01	0.004	0.011	7.0E-01
		rs35589286	128192680	INTU	G/C	0.29	PD	0.012	0.007	7.9E-02	0.013	0.008	1.2E-01	-0.003	0.012	8.2E-01
13	5q23.2(2)	rs6885843	126068500	TEX43, LMNB1	C/T	0.40	DA	0.014	0.007	3.7E-02	0.023	0.008	5.3E-03	0.001	0.013	9.7E-01
14	6p25.1	rs1294419	6742549	LY86, RREB1	C/G	0.62	NDA	0.007	0.006	2.8E-01	0.002	0.008	8.1E-01	0.011	0.012	3.4E-01
15	6p22.3	rs3819405	16399557	ATXN1	C/T	0.33	DA, PD	-0.040	0.007	1.7E-08	-0.045	0.008	6.5E-08	-0.027	0.013	3.5E-02
16	8p12	rs6558136	29523123	DUSP4, SARAF	C/T	0.65	PD	-0.056	0.007	5.9E-18	-0.062	0.008	9.3E-16	-0.042	0.012	3.6E-04
17	10p12.1	rs2642278	27936562	RAB18, MKX	T/C	0.36	DA	0.017	0.007	7.1E-03	0.014	0.008	6.6E-02	0.010	0.012	3.8E-01
18	10q21.1	rs1892368	*53674795*	PRKG1	*G/A*	*0.67*	DA	-0.034	0.007	4.8E-07	-0.033	0.008	2.6E-05	-0.033	0.012	6.1E-03
19	10q21.2(1)	rs1949355	64218423	ZNF365	G/A	0.56	NDA	0.043	0.006	3.4E-12	0.046	0.007	5.5E-10	0.022	0.011	4.9E-02
		rs2138555	64220494		A/G	0.41	PD	-0.044	0.006	1.5E-12	-0.047	0.008	3.9E-10	-0.030	0.011	8.7E-03
20	11p15.4	rs11040963	*6713214*	MRPL17, OR2AG2	*C/A*	*0.28*	DA	-0.024	0.007	4.5E-04	-0.018	0.008	2.9E-02	-0.030	0.013	1.6E-02
21	11p15.2	rs60521023	13314102	ARNTL	A/AT	0.70	NDA	0.003	0.007	6.3E-01	NA	0.008	6.3E-01	NA	0.013	2.6E-01
22	11q24.3	rs6590455	130276427	ADAMTS8	T/C	0.65	PD	0.000	0.007	9.7E-01	0.009	0.009	3.1E-01	-0.020	0.013	1.1E-01
		rs2875238	130282078		T/C	0.66	NDA	-0.007	0.007	3.1E-01	0.000	0.009	9.6E-01	-0.022	0.013	9.1E-02
23	12p12.1	rs1818476	26445564	SSPN, ITPR2	C/T	0.75	DA	0.013	0.007	7.5E-02	0.003	0.009	7.0E-01	0.024	0.013	7.0E-02
24	12q22	rs11836367	96027467	USP44, NTN4	C/T	0.33	NDA	-0.082	0.007	3.6E-36	-0.084	0.008	3.8E-27	-0.065	0.012	5.4E-08
25	13q13.3	rs10219975	36269125	NBEA, DCLK1	G/C	0.32	PD	-0.005	0.007	4.1E-01	-0.012	0.008	1.4E-01	-0.007	0.012	5.4E-01
26	14q24.1	rs75197674	68971452	RAD51B	T/C	0.21	DA	-0.062	0.008	8.3E-16	-0.062	0.009	2.2E-11	-0.047	0.014	9.5E-04
27	15q25.2	rs1812707	84547222	ADAMTSL3	C/T	0.50	NDA	-0.007	0.006	2.9E-01	-0.010	0.007	1.8E-01	-0.001	0.011	9.5E-01
28	15q26.1	rs4777948	94298239	—	G/T	0.47	PD	0.008	0.007	2.3E-01	0.006	0.008	4.7E-01	-0.001	0.013	9.4E-01
29	18q21.33	rs11877925	60896694	BCL2	G/A	0.22	DA	-0.014	0.007	6.3E-02	-0.012	0.009	1.9E-01	-0.004	0.014	7.8E-01
30	19q13.33	rs492602	49206417	FUT2	A/G	0.48	DA	0.012	0.006	6.5E-02	0.013	0.008	8.8E-02	-0.005	0.011	6.4E-01
		rs1704773	49209566	FUT2, MAMSTR	A/G	0.49	PD	0.012	0.006	4.8E-02	0.015	0.007	4.4E-02	-0.007	0.011	5.3E-01
31	20q13.13	rs17196752	48887268	SMIM25	C/T	0.20	DA	0.019	0.008	1.5E-02	0.008	0.009	3.8E-01	0.044	0.014	1.7E-03

a. Independent regions within the same cytoband are denoted by (1) or (2) in order by position.

b. Nearest flanking protein coding gene(s) within 500 kb of the lead SNP.

NA = not available.

Source of breast cancer results: http://bcac.ccge.medschl.cam.ac.uk/bcacdata/oncoarray/gwas-icogs-and-oncoarray-summary-results/

Supplementary Table 7. Association of previously reported MD loci with standardized MD phenotypes.

#	References	Region[a]	SNP	Position	Imputation r^2	Gene[b]	Ref/ Effect	Associated MD Phenotype	Reported Association			Dense Area			Non-Dense Area			Percent Density		
									Beta	***SE***	***P-value***	***Beta***	***SE***	***P-value***	***Beta***	***SE***	***P-value***	***Beta***	***SE***	***P-value***
1	Fernandez-Navarro et al (2015)	1q12-q21	rs11205277	149892872	1.00	SV2A, SF3B4	A/G	PD	OR 0.74	0.67-0.81	1.3E-10	-0.070	0.009	**9.9E-16**	-0.003	0.006	6.0E-01	-0.043	0.007	**2.6E-09**
	Lead SNP	1q21.2	rs11205303	149906413	1.00	MTMR11	T/C	***DA***, PD	—	—	—	-0.080	0.009	***6.8E-20***	-0.004	0.006	5.7E-01	-0.049	0.007	**1.5E-11**
2	Brand et al (2018)	2q14.2(1)	rs12468790	121092388	0.88	RALB, INHBB	A/G	ADV	0.08	0.01	2.1E-08	0.043	0.009	5.2E-06	0.047	0.007	**8.1E-13**	-0.001	0.008	9.4E-01
	Lead SNP		rs4849864	121089570	0.88		T/A	***NDA***	—	—	—	0.044	0.009	3.6E-06	0.049	0.007	***2.6E-13***	-0.001	0.008	9.1E-01
	Lead SNP		rs17625845	121089731	0.80		T/C	DA	—	—	—	0.072	0.012	**2.8E-09**	0.049	0.009	1.1E-08	0.016	0.010	1.2E-01
3	Lindstrom et al (2014)	4q13.3	rs10034692	75419787	1.00	AREG	A/G	DA	-0.16	0.03	2.1E-10	-0.057	0.010	**1.3E-09**	-0.030	0.007	7.1E-06	-0.019	0.008	1.7E-02
	Lead SNP		rs149689338	75424084	1.00		G/A	DA	—	—	—	-0.058	0.010	**8.2E-10**	-0.030	0.007	6.7E-06	-0.019	0.008	1.6E-02
	Lead SNP		rs71219402	75521109	0.98		G/†	***NDA***	—	—	—	0.029	0.009	1.4E-03	0.037	0.006	***1.4E-08***	-0.004	0.008	6.0E-01
4	Lindstrom et al (2014)	5q23.2(1)	rs186749	122454305	1.00	PRDM6	G/A	PD	0.10	0.02	2.5E-09	0.058	0.009	**1.3E-10**	-0.004	0.006	5.1E-01	0.039	0.007	1.7E-07
	Lead SNP		rs335143	122442322	1.00		A/C	PD	—	—	—	0.067	0.011	**1.1E-09**	-0.020	0.008	1.1E-02	0.053	0.009	**1.0E-08**
	Lead SNP		rs335160	122478676	1.00		C/A	***DA***	—	—	—	-0.074	0.010	***6.7E-14***	0.000	0.007	9.8E-01	-0.046	0.008	**1.4E-08**
5	Brand et al (2015)	6q25.1	rs9485370	149606801	1.00	TAB2	G/T	DA	-0.04	0.01	2.5E-08	-0.063	0.011	**1.1E-08**	0.016	0.008	4.1E-02	-0.047	0.009	2.1E-07
					1.00			PD	-0.05	0.01	4.8E-09									
	Lead SNP		rs4897108	149601768	1.00		G/A	DA, PD	—	—	—	-0.062	0.010	**2.0E-09**	0.028	0.007	8.8E-05	-0.054	0.008	**1.0E-10**
6	Lindstrom et al (2014)	6q25.1	rs12665607	151946629	1.00	CCDC170, ESR1	T/A	DA	0.17	0.04	1.7E-08	0.063	0.016	5.4E-05	0.046	0.011	2.9E-05	0.015	0.013	2.4E-01
	Brand et al (2015)[c]		rs60705924	151955985	0.99		A/G	DA	0.04	0.01	2.2E-08	0.043	0.009	3.1E-06	0.023	0.006	3.6E-04	0.014	0.008	7.3E-02
7	Lindstrom et al (2014)	8p11.23	rs7816345	36846109	1.00	KCNU1	C/T	NDA	-0.24	0.03	2.4E-23	0.044	0.011	7.4E-05	-0.103	0.008	**6.0E-40**	0.085	0.009	**3.6E-20**
					1.00			PD	0.08	0.02	4.7E-08									
	Lead SNP		rs10086016	36847709	1.00		T/C	NDA, PD	—	—	—	0.045	0.011	5.3E-05	-0.103	0.008	**5.8E-40**	0.085	0.009	**1.1E-20**
8	Lindstrom et al (2014, 2011)	10q21.2(2)	rs10995190	64278682	1.00	ZNF365	G/A	DA	-0.24	0.03	1.5E-16	-0.126	0.012	**9.4E-27**	-0.013	0.008	1.2E-01	-0.069	0.010	**1.6E-12**
					1.00			PD	-0.18	0.03	9.6E-10									
	Lead SNP		rs10995181	64268659	1.00		T/A	DA	—	—	—	-0.130	0.012	**4.2E-27**	-0.013	0.009	1.3E-01	-0.072	0.010	**4.2E-13**
9	Brand et al (2018)	10q25.3	rs2089176	115248851	1.00	TCF7L2, HABP2	A/G	PDV	-0.07	0.01	4.1E-09	-0.026	0.009	3.2E-03	0.020	0.006	1.1E-03	-0.029	0.007	6.1E-05
10	Lindstrom et al (2014)	11p15.5	rs3817198	1909006	0.55	LSP1	T/C	DA	0.14	0.03	9.7E-11	0.001	0.014	9.2E-01	0.009	0.009	3.7E-01	-0.003	0.011	8.1E-01
11	Lindstrom et al (2014)	12q23.2	rs703556	103011894	0.99	IGF1, PAH	A/G	DA	-0.41	0.08	3.7E-10	-0.144	0.028	3.3E-07	-0.027	0.020	1.7E-01	-0.069	0.023	3.1E-03
	Lead SNP		rs5800526	103083408	0.88		T/TG	DA	—	—	—	0.200	0.034	**3.9E-09**	0.007	0.024	7.6E-01	0.118	0.028	2.5E-05
12	Stevens et al (2012)	12q24	rs1265507	114868138	0.99	TBX5, TBX3	C/T	PD	-0.25	0.06	1.0E-08	-0.022	0.009	1.2E-02	0.025	0.006	3.5E-05	-0.028	0.007	8.3E-05
13	Brand et al (2018)	17q24.3	rs9302903	67836371	0.97	MAP2K6, KCNJ16	T/C	ADV	-0.14	0.02	5.9E-09	-0.053	0.016	9.6E-04	-0.031	0.011	6.6E-03	-0.017	0.013	1.9E-01
14	Lindstrom et al (2014)	22q13.1	rs7289126	38628306	1.00	TMEM184B	C/A	DA	-0.11	0.02	2.8E-08	-0.015	0.009	8.5E-02	0.007	0.006	2.2E-01	-0.012	0.007	8.3E-02
					1.00			PD	-0.10	0.02	4.7E-09									
15	Lindstrom et al (2014)	22q13.1	rs17001868	40778231	0.99	SGSM3	A/C	DA	-0.18	0.03	2.3E-13	-0.062	0.013	4.3E-06	-0.042	0.009	8.0E-06	-0.015	0.011	1.9E-01
	Lead SNP	22q13.1-q13.2	rs73169057	40990950	1.00	MRTFA	T/C	***NDA***	—	—	—	-0.091	0.015	**6.6E-10**	-0.059	0.010	***9.6E-09***	-0.023	0.012	6.2E-02
	Lead SNP		rs6001984	41025380	0.98		A/G	DA	—	—	—	-0.091	0.014	**8.3E-11**	-0.049	0.010	8.9E-07	-0.029	0.012	1.5E-02

a. Independent regions within the same cytoband are denoted by (1) or (2) in order by position.

b. Nearest protein coding flanking gene(s) within 500 kb of the lead SNP.

c. rs60705924 and rs12665607 (r^2 = 0.15 in Europeans) were not conditionally independently associated with DA at the genome-wide significance level of 5×10^{-8}.

Italics denote novel phenotypic association at $P < 5\times10^{-8}$; **Boldface** denotes $P < 5\times10^{-8}$.

ADV = absolute dense volume; PDV = percent dense volume; OR = odds ratio and 95% confidence interval; † Alternate allele = GTAAATGGACAA.

Supplementary Table 8. Genes regulated by lead SNPs at previously reported MD loci that were significant eQTLs[a] in mammary tissue, fibroblasts, fatty tissues, or blood.

#	References	Region[b]	SNP	Position	Gene[c]	Ref/ Effect	Associated MD phenotype	Mammary tissue	Fibroblast cells	Subcutaneous fat	Visceral fat	Whole blood
1	Fernandez-Navarro et al (2015)	1q12-q21	rs11205277	149892872	SV2A, SF3B4	A/G	PD	MTMR11 (−)	RP11-196G18.22* (+), SV2A (+)	RP11-196G18.22 (+)	—	—
	Lead SNP	1q21.2	rs11205303	149906413	MTMR11	T/C	***DA***, PD	—	RP11-196G18.22* (+)	RP11-196G18.22 (+)	—	—
3	Lindstrom et al (2014)	4q13.3	rs10034692	75419787	AREG	A/G	DA	—	AREG (+)	—	AREG (+)	—
	Lead SNP		rs149689338	75424084		G/A	DA	—	AREG (+)	—	AREG (+)	—
	Lead SNP		rs71219402	75521109		G/†	***NDA***	—	—	—	—	—
4	Lindstrom et al (2014)	5q23.2(1)	rs186749	122454305	PRDM6	G/A	PD	—	—	—	—	—
	Lead SNP		rs335143	122442322		A/C	PD	SNX2 (+)	—	—	—	—
	Lead SNP		rs335160	122478676		C/A	***DA***	—	—	—	—	—
6	Lindstrom et al (2014)	6q25.1	rs12665607	151946629	CCDC170, ESR1	T/A	DA	CCDC170 (+)	—	—	—	—
	Brand et al (2015)[d]		rs60705924	151955985		A/G	DA	—	—	CCDC170 (+)	—	—
7	Lindstrom et al (2014)	8p11.23	rs7816345	36846109	KCNU1	C/T	NDA, PD	ZNF703* (−)	ERLIN2 (−)	RP11-419C23.1* (−), ZNF703* (−), RP11-863K10.7 (−)	—	—
	Lead SNP		rs10086016	36847709		T/C	NDA, PD	ZNF703* (−)	ERLIN2 (−)	RP11-419C23.1* (−), ZNF703* (−), RP11-863K10.7 (−)	—	—
10	Lindstrom et al (2014)	11p15.5	rs3817198	1909006	LSP1	T/C	DA	PRR33 (−)	—	—	—	—
12	Stevens et al (2012)	12q24	rs1265507	114868138	TBX5, TBX3	C/T	PD	TBX5 (+)	—	—	—	—
14	Lindstrom et al (2014)	22q13.1	rs7289126	38628306	TMEM184B	C/A	DA, PD	—	MAFF (−), PLA2G6* (+), TMEM184B* (−)	MAFF (−), TMEM184B* (−)	CARD10 (+), TMEM184B (-)	—

a. Significant (FDR < 0.05) eQTLs identified by computing q-values for all SNP-gene pairs involving the top density SNPs that were tested in mammary tissue, primary fibroblast cell culture, subcutaneous fat, visceral fat, or whole blood samples in GTEx V7; up- or down-regulation of gene expression associated with the alternate allele is indicated by (+) or (-), respectively, after the target gene name. Source: https://gtexportal.org/
b. Independent regions within the same cytoband are denoted by (1) or (2) in order by position.
c. Nearest flanking protein coding gene(s) within 500 kb of the lead SNP.
d. rs60705924 and rs12665607 (r^2 = 0.15 in Europeans) were not conditionally independently associated with DA at the genome-wide significance level of 5×10-8. rs6930633 was used as a proxy for rs60705924 (r^2 = 1).
Italics denote novel phenotypic association at $P < 5\times10^{-8}$
* Genes with a significant eQTL identified by GTEx V7 analyses of all possible SNP-gene pairs genome-wide.

Supplementary Table 9. Cell-specific enrichment of MD loci within gene regulatory regions.[a]

Loci	Cell line	Description[b]	Observed[c]	Expected[d]	Empirical P[d]
**DA**	NHDF-Ad*	Adult normal human dermal fibroblasts	12	5.1	0.0008
	HPF*	Human pulmonary fibroblasts	9	3.8	0.0045
	WI-38*	Embryonic lung fibroblast cells, hTERT immortalized, includes Raf1 construct	9	4.0	0.0073
	HCF*	Human cardiac fibroblasts	9	4.1	0.0083
	HFF_Myc*	Human foreskin fibroblast	10	4.8	0.0088
	HPdLF	Normal human periodontal ligament fibroblast cells	9	4.1	0.0096
**NDA**	WI-38_TAM	Embryonic lung fibroblasts immortilized hTERT - Tamoxifen treated	10	2.8	**<0.0001**
	AG09319*	Adult human gum tissue dermal fibroblasts	8	2.1	**0.0001**
	HCF*	Human cardiac fibroblasts	8	2.5	**0.0003**
	HFF_Myc*	Human foreskin fibroblast	9	3.0	0.0006
	AoAF	Normal human aortic adventitial fibroblast cells	8	2.5	0.0010
	FibroP	Normal fibroblasts taken from individuals with Parkinson's disease, AG20443, AG08395 and AG08396 were pooled for this sample	9	3.4	0.0014
	HPAF	Human pulmonary artery fibroblasts	8	2.7	0.0014
	NHDF-neo*	Neonatal human dermal fibroblasts	8	2.7	0.0014
	HCM	Human cardiac myocytes	8	2.8	0.0016
	HAEpiC	Human amniotic epithelial cells	8	2.9	0.0022
	AG04450	Fetal lung fibroblast	7	2.2	0.0023
	WI-38*	Embryonic lung fibroblast cells, hTERT immortalized, includes Raf1 construct	7	2.4	0.0035
	HConF	Human conjunctival fibroblast	7	2.3	0.0036
	HPF*	Human pulmonary fibroblasts	7	2.3	0.0037
	BJ	Skin fibroblast	7	2.4	0.0038
	HVMF	Human villous mesenchymal fibroblast cells	7	2.4	0.0043
	NHDF-Ad*	Adult normal human dermal fibroblasts	8	3.2	0.0050
	HSMM_emb	Embryonic myoblast	6	1.9	0.0056
	HMF	Human mammary fibroblast	7	2.6	0.0066
**PD**	HFF_Myc*	Human foreskin fibroblast	8	3.3	0.0053
	NHDF-Ad*	Adult normal human dermal fibroblasts	8	3.5	0.0073
**MD**	HFF_Myc*	Human foreskin fibroblast	19	7.5	**<0.0001**
	NHDF-Ad*	Adult normal human dermal fibroblasts	18	8.1	**0.0001**
	AG09319*	Adult human gum tissue fibroblasts	13	5.4	0.0005
	NHDF-neo*	Neonatal human dermal fibroblasts	15	6.9	0.0011
	Th0	Unstimulated Th0 cells isolated from adults' blood	14	6.7	0.0018
	HCF*	Human cardiac fibroblasts	14	6.5	0.0021
	HFF	Human foreskin fibroblast	14	7.0	0.0033
	WI-38*	Embryonic lung fibroblast cells, hTERT immortalized, includes Raf1 construct	13	6.2	0.0050
	Monocytes-CD14+	Monocytes-CD14+ are CD14-positive cells from human leukapheresis product	11	4.9	0.0061
	HMVECdBl-Ad	Normal adult human blood microvascular endothelial cells, dermal-derived	12	6.0	0.0081
	HPF*	Human pulmonary fibroblasts	12	5.9	0.0081

a. Independent prior and new loci for MD phenotypes were analyzed using UES (Hayes et al. 2015; https://github.com/robertkleinlab/uesEnrichment). Results are shown for ENCODE cell lines derived from normal human cells that had suggestive evidence of enrichment for MD loci in regulatory regions at $P < 0.01$.

b. Cell line descriptions from Thurman et al (2012).

c. Observed number of loci overlapping regulatory regions.

d. Expected number of loci overlapping regulatory regions and empirical P-values computed using 10,000 simulations under the null hypothesis.

Boldface denotes significant enrichment at the Bonferroni-corrected threshold of $P < 0.0004$ for the 125 cell lines tested.

* Denotes cell lines with evidence of regulatory region enrichment for more than one set of MD loci.

Supplementary Table 10. Gene set enrichment analysis[a] of candidate genes for DA.

Pathway	Genes Contributing to Enrichment	Database: Accession	Terms	Fold Enrichment[b]	FDR
**Reproductive process**	BCL2, ESR1, INHBB, MAP2K6	GO:0022602	ovulation cycle process	23.0	0.011
		GO:0042698	ovulation cycle	17.8	0.023
	AREG, BCL2, ESR1, IGF1, INHBB, OSR1, PPARG	GO:0003006	developmental process involved in reproduction	5.1	0.031
	AREG, BCL2, ESR1, IGF1, INHBB, MAP2K6, OSR1, PLA2G6, PPARG, RAD51B	GO:0044702	single organism reproductive process	3.8	0.013
	AREG, BCL2, ESR1, IGF1, INHBB, MAFF, MAP2K6, OSR1, PLA2G6, PPARG, RAD51B	GO:0022414	reproductive process	3.7	0.006
		GO:0000003	reproduction	3.7	0.006
**Inflammatory response**	IGF1, ITPR2, MAP2K6, PLA2G6	hsa04750	Inflammatory mediator regulation of TRP channels	14.8	0.021
**Apoptosis**	BCL2, FAF1, IGF1, INHBB, MRTFA, PLA2G6	GO:2001233	regulation of apoptotic signaling pathway	7.4	0.018
	BCL2, CARD10, ESR1, FAF1, IGF1, INHBB, MAP2K6, MRTFA, OSR1, PLA2G6, PPARG	GO:0042981	regulation of apoptotic process	3.7	0.007
		GO:0043067	regulation of programmed cell death	3.6	0.008
		GO:0010941	regulation of cell death	3.4	0.013
	BCL2, CARD10, ESR1, FAF1, IGF1, INHBB, MAP2K6, MRTFA, OSR1, PLA2G6, PPARG, RALB	GO:0012501	programmed cell death	3.0	0.017
		GO:0008219	cell death	2.9	0.026
**Anatomical structure development**	BCL2, IGF1, MAFF, MAMSTR, MKX, MRTFA, PRDM6	GO:0061061	muscle structure development	5.5	0.021
**Regulation of metabolic process**	AREG, BCL2, CARD10, IGF1, INHBB, MAP2K6, PLA2G6, RALB, TAB2	GO:0042327	positive regulation of phosphorylation	4.5	0.009
		GO:0010562	positive regulation of phosphorus metabolic process	4.0	0.020
		GO:0045937	positive regulation of phosphate metabolic process	4.0	0.020
	BCL2, CARD10, IGF1, INHBB, MAP2K6, PLA2G6, RALB, TAB2	GO:0001934	positive regulation of protein phosphorylation	4.2	0.036
	AREG, BCL2, CARD10, FAF1, IGF1, INHBB, MAP2K6, PLA2G6, RALB, TAB2	GO:0042325	regulation of phosphorylation	3.3	0.030
	BCL2, CARD10, ESR1, IGF1, INHBB, MAP2K6, PLA2G6, PPARG, RALB, TAB2	GO:0032270	positive regulation of cellular protein metabolic process	3.3	0.034
	AREG, ESR1, IGF1, MAFF, MAMSTR, MAP2K6, MRTFA, OSR1, PLA2G6, PPARG, TET3	GO:0009891	positive regulation of biosynthetic process	2.9	0.042
	AREG, BCL2, CARD10, ESR1, IGF1, INHBB, MAFF, MAMSTR, MAP2K6, MRTFA, OSR1, PLA2G6, PPARG, RALB, TAB2, TET3	GO:0031325	positive regulation of cellular metabolic process	2.6	0.005
		GO:0010604	positive regulation of macromolecule metabolic process	2.6	0.005
		GO:0009893	positive regulation of metabolic process	2.4	0.011
**Regulation of biological process**	BCL2, FAF1, IGF1, INHBB, ITPR2, KCNJ16, MAP2K6, OSR1, PLA2G6, PPARG, RALB, SGSM3	GO:0051049	regulation of transport	3.1	0.013
	AREG, ATXN1, BCL2, ESR1, FAF1, IGF1, INHBB, ITPR2, MAP2K6, MRTFA, OSR1, PLA2G6, PPARG, PRDM6, PRKG1, RASIP1, SGSM3, TAB2	GO:0048523	negative regulation of cellular process	2.0	0.048
**Regulation of molecular function**	BCL2, CARD10, ESR1, FAF1, IGF1, MAP2K6, MRTFA, PPARG, PRKG1, RALB, RASIP1, SGSM3, TAB2	GO:0050790	regulation of catalytic activity	2.6	0.035
	BCL2, CARD10, ESR1, FAF1, IGF1, MAP2K6, MRTFA, OSR1, PLA2G6, PPARG, PRKG1, RALB, RASIP1, SGSM3, TAB2	GO:0065009	regulation of molecular function	2.4	0.019
**Signaling**	BCL2, CARD10, ESR1, FAF1, IGF1, INHBB, ITPR2, MAP2K6, PLA2G6, PRKG1, RAB18, RALB, SGSM3, TAB2, TNS1	GO:0035556	intracellular signal transduction	2.6	0.009
	AREG, BCL2, CARD10, ESR1, FAF1, IGF1, INHBB, ITPR2, LSP1, MAP2K6, MRTFA, OR2AG2, OSR1, PLA2G6, PPARG, PRKG1, RAB18, RALB, RASIP1, SGSM3, TAB2, TNS1	GO:0007165	signal transduction	1.8	0.029

a. Gene sets from the Gene Ontology (GO), KEGG (hsa), Reactome (R-HSA) and Biocarta databases were analyzed using DAVID 6.8. Results are shown for pathways with FDR <0.05. Tested genes included the nearest flanking genes within 500 kb of novel or previously reported DA loci, and the target genes for significant eQTLs in mammary tissue, primary fibroblast cells, subcutaneous fat, visceral fat, or whole blood.

b. Fold enrichment denotes the observed proportion of tested genes in the pathway, divided by the expected proportion of all genes in the pathway.

Supplementary Table 11. Gene set enrichment analysis[a] of candidate genes for NDA.

Pathway	Genes Contributing to Enrichment	Database: Accession	Terms	Fold Enrichment[b]	FDR
**Mammary gland development**	AREG, RREB1, ZNF703	GO:0033598	mammary gland epithelial cell proliferation	84.2	0.0087
	AREG, GLI2, RREB1, ZNF703	GO:0061180	mammary gland epithelium development	41.0	0.0018
	AREG, GLI2, NCOA1, RREB1, ZNF703	GO:0030879	mammary gland development	24.5	0.0007
	AREG, GLI2, NCOA1, NTN4, RREB1, ZNF703	GO:0048732	gland development	9.2	0.0056
**Glycoprotein metabolic process**	ADAMTS8, ADAMTS9, ADAMTSL3	R-HSA-5173214	O-glycosylation of TSR domain-containing proteins	34.9	0.0293
**Response to organic compounds**	INHBB, NCOA1, PPARG, RALB	GO:0031669	cellular response to nutrient levels	16.2	0.0270
		GO:0031668	cellular response to extracellular stimulus	13.7	0.0431
	ADCY3, ARNTL, GLI2, NCOA1, PPARG, RALB, ZNF703	GO:0071407	cellular response to organic cyclic compound	8.7	0.0015
	ADCY3, ARNTL, LY86, NCOA1, PPARG, ZNF703	GO:0071396	cellular response to lipid	7.6	0.0133
	ADCY3, AREG, ARNTL, GLI2, NCOA1, PPARG, RALB, ZNF703	GO:0014070	response to organic cyclic compound	5.7	0.0044
	ADCY3, AREG, ARNTL, LY86, NCOA1, PPARG, ZNF703	GO:0033993	response to lipid	5.3	0.0223
**Cell differentiation**	ADAMTS9, ARNTL, BTC, GLI2, NCOA1, PPARG, RREB1, ZNF703	GO:0045597	positive regulation of cell differentiation	6.1	0.0028
	ADAMTS9, AREG, ARNTL, BTC, GLI2, NCOA1, PPARG, RREB1, ZNF703	GO:0045595	regulation of cell differentiation	3.8	0.0214
**Cell development**	AREG, ARNTL, GLI2, INHBB, MRTFA, NCOA1, NTN4, PPARG, RREB1, ZNF703	GO:0048468	cell development	3.3	0.0237
	ADAMTS9, AREG, ARNTL, BTC, GLI2, INHBB, MRTFA, NCOA1, NTN4, PPARG, RREB1, SPAG17, WARS2, ZNF703	GO:0048869	cellular developmental process	2.3	0.0338
**Reproductive process**	ADCY3, AREG, ARNTL, GLI2, INHBB, KCNU1, NCOA1, PPARG	GO:0044702	single organism reproductive process	4.2	0.0272
**Regulation of metabolic process**	ADCY3, AREG, ARNTL, ERLIN2, GLI2, MRTFA, NCOA1, PPARG, RREB1	GO:0045935	positive regulation of nucleobase-containing compound metabolic process	3.5	0.0338
		GO:0031328	positive regulation of cellular biosynthetic process	3.4	0.0439
		GO:0009891	positive regulation of biosynthetic process	3.3	0.0490
	ADCY3, AREG, ARNTL, DNAJC27, ERLIN2, GLI2, INHBB, MRTFA, NCOA1, PPARG, RALB, RREB1	GO:0031325	positive regulation of cellular metabolic process	2.7	0.0227
		GO:0010604	positive regulation of macromolecule metabolic process	2.7	0.0239
		GO:0009893	positive regulation of metabolic process	2.5	0.0413
**Regulation of biological process**	ADAMTS9, ADCY3, AREG, ARNTL, BTC, DNAJC27, ERLIN2, GLI2, INHBB, LY86, MRTFA, NCOA1, PPARG, RALB, RREB1, ZNF703	GO:0048522	positive regulation of cellular process	2.2	0.0147
		GO:0048518	positive regulation of biological process	2.0	0.0487

a. Gene sets from the Gene Ontology (GO), KEGG (hsa), Reactome (R-HSA) and Biocarta databases were analyzed using DAVID 6.8. Results are shown for pathways with FDR <0.05. Tested genes included the nearest flanking genes within 500 kb of novel or previously reported NDA loci, and the target genes for significant eQTLs in mammary tissue, primary fibroblast cell lines, subcutaneous fat, visceral fat, or whole blood.

b. Fold enrichment denotes the observed proportion of tested genes in the pathway, divided by the expected proportion of all genes in the pathway.

Supplementary Table 12. Gene set enrichment analysis[a] of candidate genes for PD.

Pathway	Genes Contributing to Enrichment	Database: Accession	Terms	Fold Enrichment[b]	FDR
**Anatomical structure development**	OSR1, TBX3, TBX5	GO:0035115	embryonic forelimb morphogenesis	49.2	0.026
		GO:0035136	forelimb morphogenesis	39.4	0.040
	INTU, OSR1, TBX3, TBX5	GO:0035113	embryonic appendage morphogenesis	16.4	0.028
		GO:0030326	embryonic limb morphogenesis	16.4	0.028
		GO:0035107	appendage morphogenesis	14.2	0.041
		GO:0035108	limb morphogenesis	14.2	0.041
**Epithelium development**	INTU, MAFF, OSR1, PPARG, RASIP1, TBX3, TBX5, ZNF703	GO:0060429	epithelium development	4.0	0.044
**Regulation of metabolic process**	ADCY3, ERLIN2, MAFF, MAMSTR, OSR1, PLA2G6, PPARG, SF3B4, TBX3, TBX5, TCF7L2	GO:0051173	positive regulation of nitrogen compound metabolic process	3.3	0.015
	ADCY3, ERLIN2, MAFF, MAMSTR, OSR1, PPARG, SF3B4, TBX3, TBX5, TCF7L2	GO:0045935	positive regulation of nucleobase-containing compound metabolic process	3.2	0.039
	ADCY3, CARD10, DNAJC27, ERLIN2, MAFF, MAMSTR, OSR1, PLA2G6, PPARG, SF3B4, TAB2, TBX3, TBX5, TCF7L2	GO:0031325	positive regulation of cellular metabolic process	2.6	0.015
		GO:0010604	positive regulation of macromolecule metabolic process	2.6	0.016
		GO:0009893	positive regulation of metabolic process	2.4	0.030

a. Gene sets from the Gene Ontology (GO), KEGG (hsa), Reactome (R-HSA) and Biocarta databases were analyzed using DAVID 6.8. Results are shown for pathways with FDR <0.05. Tested genes included the nearest flanking genes within 500 kb of novel or previously reported DA loci, and the target genes for significant eQTLs in mammary tissue, primary fibroblast cell lines, subcutaneous fat, visceral fat, or whole blood.

b. Fold enrichment denotes the observed proportion of tested genes in the pathway, divided by the expected proportion of all genes in the pathway.

Supplementary Table 13. Sensitivity of NDA loci to further adjustment for BMI.

Region[a]	SNP	Position	Gene[b]	Ref/ Alt	AAF	Association with standardized NDA[c]			Association with BMI (kg/m^2)[d]		
						Beta	*SE*	*P-value*	*Beta*	*SE*	*P-value*
1p36.11	rs61777307	23959319	MDS2	G/A	0.24	-0.040	0.007	7.6E-09	0.038	0.066	5.6E-01
1p12(1)	rs10802015	118782659	SPAG17	C/T	0.34	-0.043	0.006	1.5E-11	0.089	0.060	1.4E-01
1p12(2)	rs1779445	119508412	TBX15	T/C	0.80	0.051	0.007	4.3E-12	-0.008	0.070	9.1E-01
2p23.3	rs6718628	25131170	ADCY3	C/G	0.40	0.040	0.006	1.9E-10	0.210	0.059	3.4E-04
2q14.2(2)	rs11123556	121245996	INHBB, GLI2	A/G	0.89	-0.074	0.010	1.1E-14	-0.121	0.090	1.8E-01
3p25.2	rs199689761	12441088	PPARG	T/TA	0.16	-0.047	0.008	1.7E-08	-0.133	0.078	8.7E-02
3p14.1	rs4132228	64708114	ADAMTS9	C/T	0.31	-0.044	0.006	4.8E-12	0.165	0.061	6.4E-03
6p25.1	rs1294419	6742549	LY86, RREB1	C/G	0.62	0.039	0.006	2.3E-10	-0.004	0.058	9.4E-01
10q21.2(1)	rs1949355	64218423	ZNF365	G/A	0.56	-0.039	0.006	1.0E-10	0.044	0.056	4.4E-01
11p15.2	rs60521023	13314102	ARNTL	A/AT	0.70	0.040	0.007	2.0E-09	0.022	0.062	7.3E-01
11q24.3	rs2875238	130282078	ADAMTS8	T/C	0.66	0.040	0.006	4.4E-10	-0.099	0.060	1.0E-01
12q22	rs11836367	96027467	USP44, NTN4	C/T	0.33	0.042	0.007	6.0E-09	0.033	0.067	6.3E-01
15q25.2	rs1812707	84547222	ADAMTSL3	C/T	0.50	0.034	0.006	3.5E-08	0.010	0.057	8.6E-01

a. Independent regions within the same cytoband are denoted by (1) or (2) in order by position.

b. Nearest flanking protein coding gene(s) within 500 kb of the lead SNP.

c. Meta-analysis of Hologic and GE studies, adjusted for ln(BMI), BMI, BMI^2, BMI^3, image batch, age at mammography, genotyping reagent kit, and 10 principal components of ancestry.

d. Meta-analysis of Hologic and GE studies, adjusted for age at mammography, genotyping reagent kit, and 10 principal components of ancestry.

Supplementary Table 14. Sensitivity analyses of Mendelian randomization estimates for the association of MD phenotypes with breast cancer risk.

Method[a]	DA		NDA		PD	
	OR[b] (95% CI)	*P*-value	OR[b] (95% CI)	*P*-value	OR[b] (95% CI)	*P*-value
Weighted median	1.45 (1.30−1.61)	0.000	0.84 (0.73−0.98)	0.029	1.68 (1.44−1.96)	0.000
Simple median	1.49 (1.33−1.67)	0.000	0.82 (0.70−0.98)	0.025	1.62 (1.39−1.89)	0.000
Weighted mode-based estimate	1.51 (1.08−2.13)	0.017	0.90 (0.76−1.06)	0.196	1.55 (1.19−2.02)	0.001
Simple mode-based estimate	1.39 (0.98−1.96)	0.062	0.90 (0.75−1.07)	0.224	1.42 (1.14−1.77)	0.002

a. Analyses were performed using median (Bowden 2016) and mode-based (Hartwig 2017) methods and the Mendelian Randomization R package.

b. Odds ratio (95% confidence interval) for breast cancer associated with each standard deviation increment of the MD phenotype.

Supplementary Table 15. Association[a] of novel alleles with MD phenotypes, stratified by menopausal status.

Region[b]	SNP	Position	Imputation r^2	Gene[c]	Ref/Alt	AAF	Premenopause (N = 4,748) *Beta*	*SE*	*P-value*	Postmenopause (N = 19,444) *Beta*	*SE*	*P-value*	Interaction *P-value*
Dense Area (n=16)													
1p32.3	rs6703250	51418472	0.95	FAF1	C/T	0.55	-0.041	0.019	3.2E-02	-0.057	0.010	1.1E-08	0.4606
2p24.1	rs11684853	19310918	1.00	OSR1	G/T	0.55	0.046	0.019	1.3E-02	0.047	0.010	9.9E-07	0.8667
2p13.1	rs6715731	74346404	0.99	TET3, BOLA3	G/T	0.42	-0.013	0.019	5.0E-01	-0.056	0.010	1.4E-08	0.0679
2q35	rs66470855*	218266438	0.99	TNS1	TG/T	0.26	0.064	0.021	2.8E-03	0.059	0.011	8.9E-08	0.9570
3p25.2	rs67901221	12416550	0.98	PPARG	A/G	0.15	0.054	0.026	3.8E-02	0.074	0.013	4.0E-08	0.4722
4q28.1	rs1503613	127772368	1.00	—	G/T	0.47	0.052	0.019	5.3E-03	0.052	0.010	6.8E-08	0.8155
5q23.2(2)	rs6885843	126068500	1.00	TEX43, LMNB1	C/T	0.40	0.042	0.019	2.9E-02	0.049	0.010	7.8E-07	0.9656
6p22.3	rs3819405*	16399557	1.00	ATXN1	C/T	0.33	-0.058	0.020	3.1E-03	-0.071	0.010	5.7E-12	0.6187
10p12.1	rs2642278	27936562	1.00	RAB18, MKX	T/C	0.36	0.047	0.019	1.5E-02	0.049	0.010	9.2E-07	0.9717
10q21.1	rs1892368	53674795	1.00	PRKG1	G/A	0.67	-0.068	0.020	6.1E-04	-0.047	0.010	3.8E-06	0.4750
11p15.4	rs11040963	6713214	0.99	MRPL17, OR2AG2	C/A	0.28	-0.035	0.021	9.6E-02	-0.057	0.011	1.6E-07	0.2882
12p12.1	rs1818476	26445564	0.99	SSPN, ITPR2	C/T	0.75	-0.049	0.022	2.5E-02	-0.059	0.011	1.7E-07	0.5065
14q24.1	rs75197674	68971452	0.91	RAD51B	T/C	0.21	-0.033	0.024	1.7E-01	-0.071	0.012	1.1E-08	0.1195
18q21.33	rs11877925	60896694	0.98	BCL2	G/A	0.22	-0.053	0.023	2.0E-02	-0.061	0.012	1.5E-07	0.6964
19q13.33	rs492602	49206417	1.00	FUT2	A/G	0.48	-0.080	0.019	2.0E-05	-0.054	0.010	2.9E-08	0.2547
20q13.13	rs17196752	48887268	0.95	SMIM25	C/T	0.20	0.046	0.024	5.7E-02	0.070	0.012	1.9E-08	0.3205
Nondense Area (n=13)													
1p36.11	rs61777307	23959319	1.00	MDS2	G/A	0.24	-0.034	0.016	3.7E-02	-0.044	0.008	2.1E-08	0.4302
1p12(1)	rs10802015	118782659	0.95	SPAG17	C/T	0.34	-0.049	0.015	8.5E-04	-0.038	0.007	1.6E-07	0.5267
1p12(2)	rs1779445	119508412	0.99	TBX15	T/C	0.80	0.047	0.017	5.7E-03	0.053	0.008	3.0E-10	0.7452
2p23.3	rs6718628	25131170	0.94	ADCY3	C/G	0.40	0.053	0.014	2.0E-04	0.038	0.007	6.8E-08	0.3487
2q14.2(2)	rs11123556	121245996	1.00	INHBB, GLI2	A/G	0.89	-0.067	0.022	2.4E-03	-0.077	0.011	8.7E-13	0.7321
3p25.2	rs199689761	12441088	0.94	PPARG	T/TA	0.16	-0.030	0.019	1.2E-01	-0.049	0.009	1.1E-07	0.5472
3p14.1	rs4132228	64708114	1.00	ADAMTS9	C/T	0.31	-0.056	0.015	1.7E-04	-0.045	0.007	4.9E-10	0.5028
6p25.1	rs1294419	6742549	0.99	LY86, RREB1	C/G	0.62	0.044	0.014	1.8E-03	0.037	0.007	6.3E-08	0.7504
10q21.2(1)	rs1949355	64218423	0.99	ZNF365	G/A	0.56	-0.021	0.014	1.3E-01	-0.043	0.007	2.3E-10	0.1344
11p15.2	rs60521023	13314102	0.96	ARNTL	A/AT	0.70	0.052	0.015	5.8E-04	0.038	0.007	2.6E-07	0.3826
11q24.3	rs2875238	130282078	0.94	ADAMTS8	T/C	0.66	0.044	0.015	3.2E-03	0.042	0.007	1.0E-08	0.9037
12q22	rs11836367	96027467	0.78	USP44, NTN4	C/T	0.33	0.078	0.016	1.9E-06	0.035	0.008	2.1E-05	0.0250
15q25.2	rs1812707	84547222	0.98	ADAMTSL3	C/T	0.50	0.024	0.014	8.4E-02	0.036	0.007	1.4E-07	0.3691
Percent Density (n=12)													
2p24.1	rs34331777	19441251	0.99	OSR1	CA/C	0.60	0.044	0.015	4.2E-03	0.042	0.008	2.2E-07	0.9646
2p23.3	rs11676272	25141538	0.98	ADCY3	A/G	0.47	-0.052	0.015	5.5E-04	-0.041	0.008	4.9E-07	0.5365
2q35	rs66470855*	218266438	0.99	TNS1	TG/T	0.26	0.050	0.017	4.2E-03	0.043	0.009	2.5E-06	0.8119
3p25.2	rs76643909	12441367	0.96	PPARG	T/G	0.16	0.043	0.021	3.8E-02	0.075	0.011	1.8E-11	0.2810
4q28.1	rs35589286	128192680	0.95	INTU	G/C	0.29	0.036	0.017	3.4E-02	0.047	0.009	2.6E-07	0.5869
6p22.3	rs3819405*	16399557	1.00	ATXN1	C/T	0.33	-0.044	0.016	5.2E-03	-0.063	0.009	2.0E-13	0.2918
8p12	rs6558136	29523123	0.98	DUSP4, SARAF	C/T	0.65	-0.067	0.016	2.4E-05	-0.035	0.008	4.2E-05	0.0646
10q21.2(1)	rs2138555	64220494	1.00	ZNF365	A/G	0.41	-0.037	0.015	1.7E-02	-0.062	0.008	1.8E-14	0.1645
11q24.3	rs6590455	130276427	0.99	ADAMTS8	T/C	0.65	-0.027	0.016	9.1E-02	-0.044	0.008	2.3E-07	0.4458
13q13.3	rs10219975	36269125	0.93	NBEA, DCLK1	G/C	0.32	-0.026	0.017	1.2E-01	-0.050	0.009	1.9E-08	0.1881
15q26.1	rs4777948	94298239	0.96	—	G/T	0.47	0.049	0.015	1.7E-03	0.045	0.008	3.4E-08	0.8491
19q13.33	rs1704773	49209566	0.95	FUT2, MAMSTR	A/G	0.49	-0.037	0.016	1.7E-02	-0.045	0.008	6.4E-08	0.5009

a. Meta-analysis of standardized MD phenotypes using R, adjusted for ln(BMI), age at mammography, image batch, genotyping reagent kit, and 10 principal components of ancestry.
b. Independent regions within the same cytoband are denoted by (1) or (2) in order by position.
c. Nearest flanking protein coding gene(s) within 500 kb of the lead SNP.
* Denotes lead SNPs associated with both DA and PD at $p < 5\times10^{-8}$.

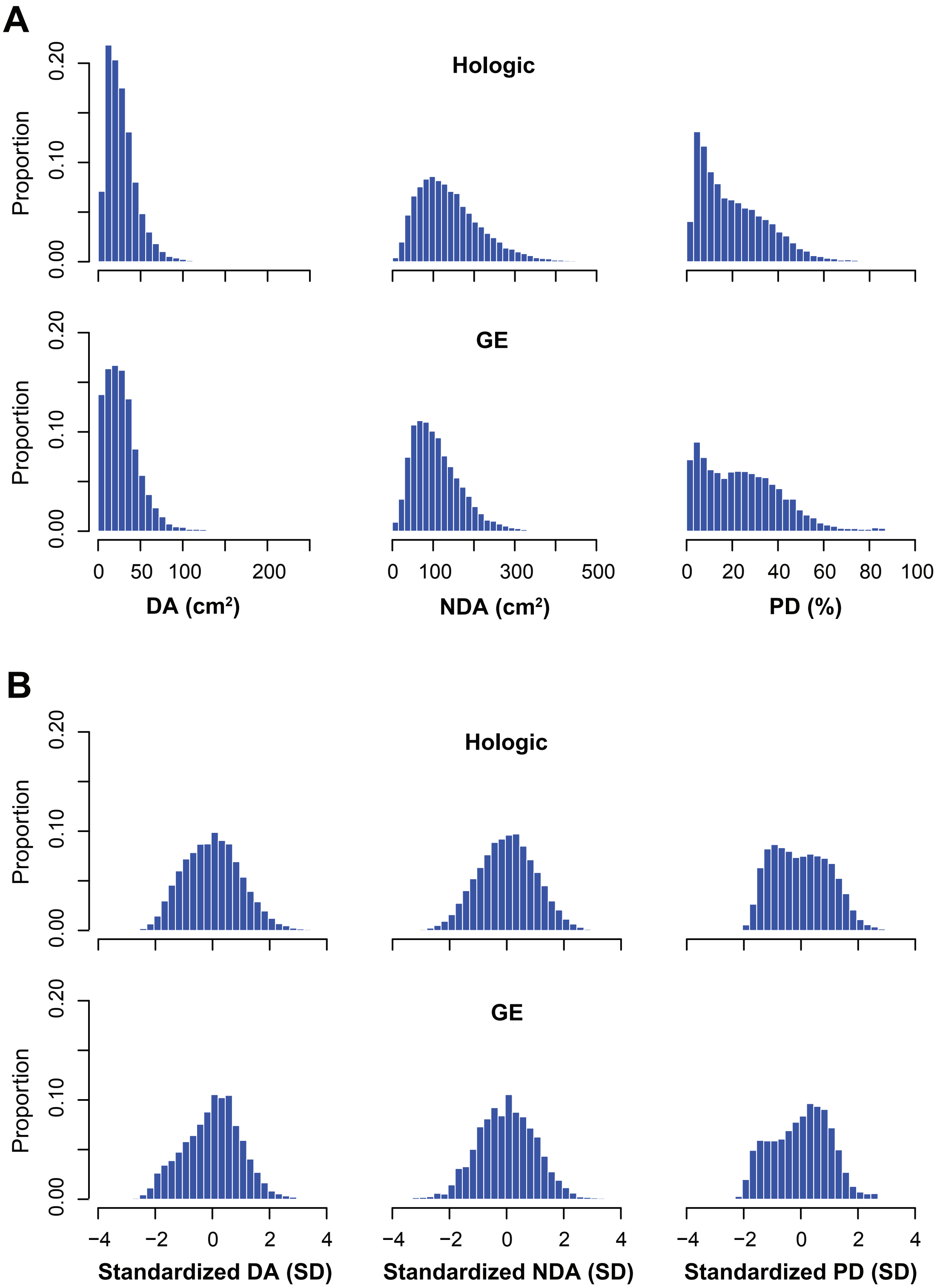

Supplementary Figure 1. Distribution of dense area (DA), nondense area (NDA) and percent density (PD) measured from Hologic and GE mammograms before (**A**) and after (**B**) transformation to standard normal distributions. DA, NDA and PD distributions were normalized using fifth-, cube- and cube-root transformations for Hologic images, and cube-, cube- and square-root transformations for GE images, respectively, and standardized to attain a mean of zero and variance of one.

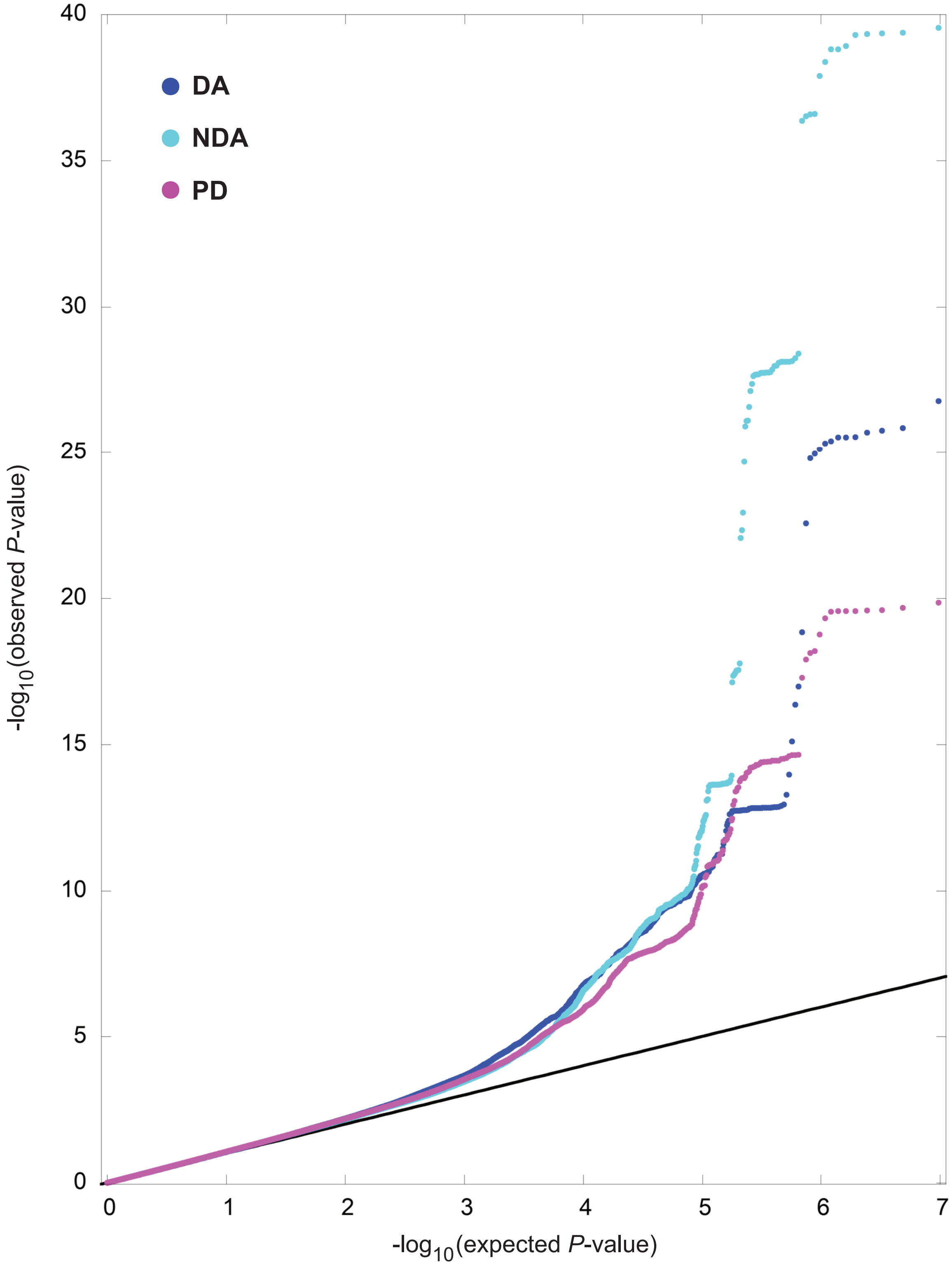

Supplementary Figure 2. Quantile-quantile plots of the GWAS meta-analysis results for dense area (DA), nondense area (NDA) and percent density (PD). The estimated genomic inflation factors were 1.06, 1.08 and 1.07 for DA, NDA and PD, respectively.

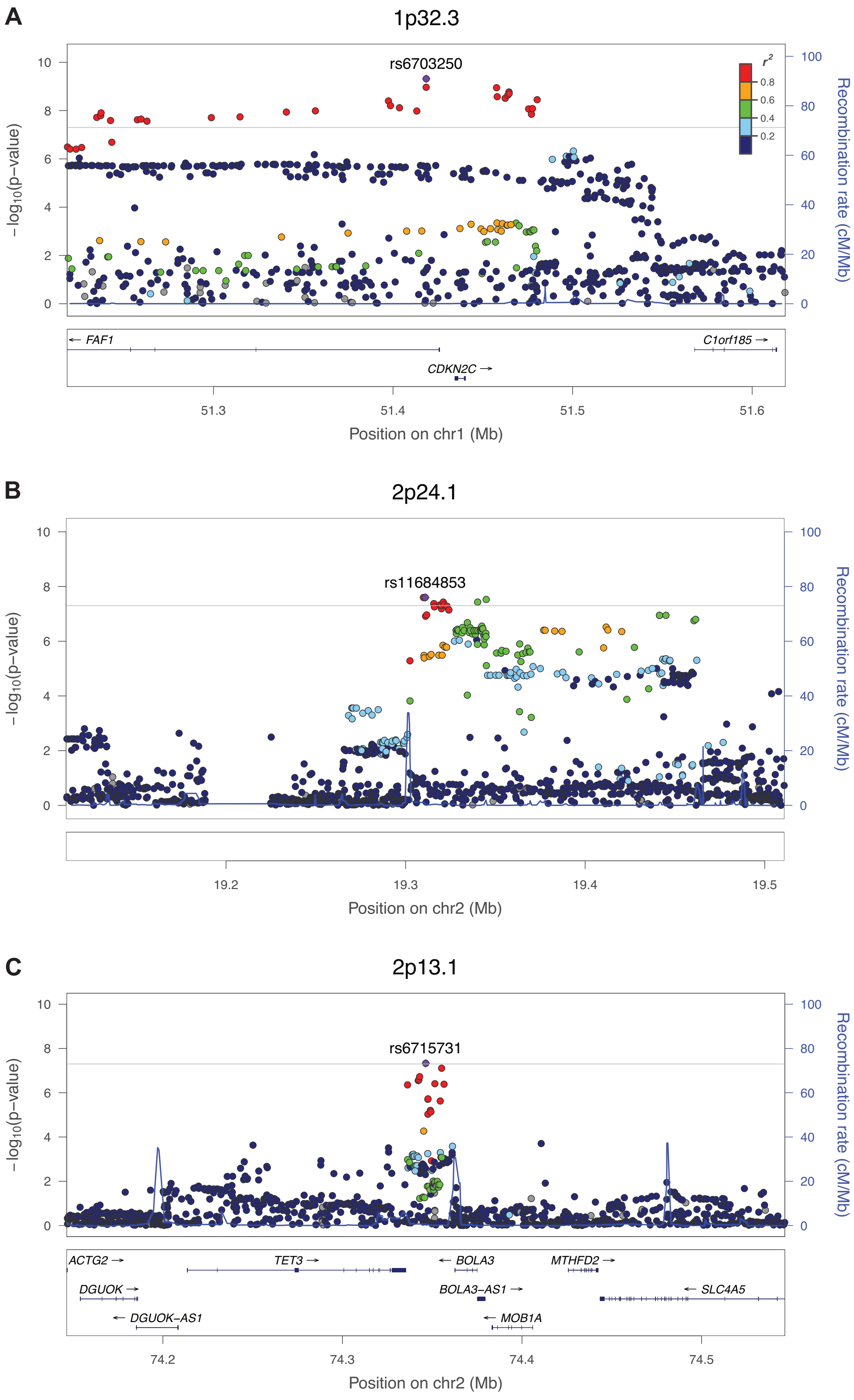

Supplementary Figure 3: Regional association plots for 16 loci (**A-P**) associated with dense area. Colors indicate linkage disequilibrium (LD; r^2) in Europeans with the referent SNP (purple). The referent was the top SNP except where a proxy (parentheses) was used because LD information was unavailable (grey) for the top SNP.

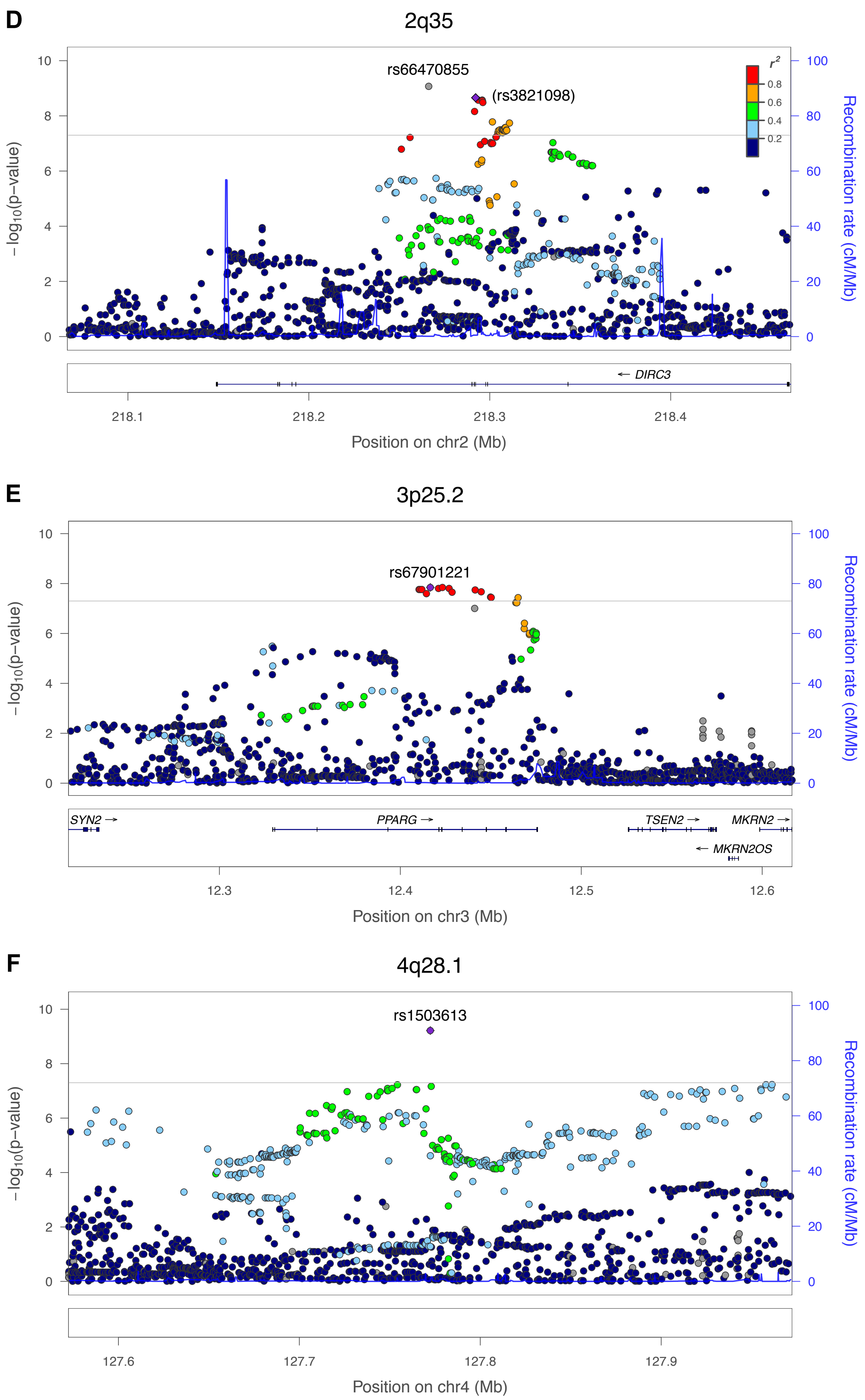

Supplementary Figure 3: Continued from previous page.

G

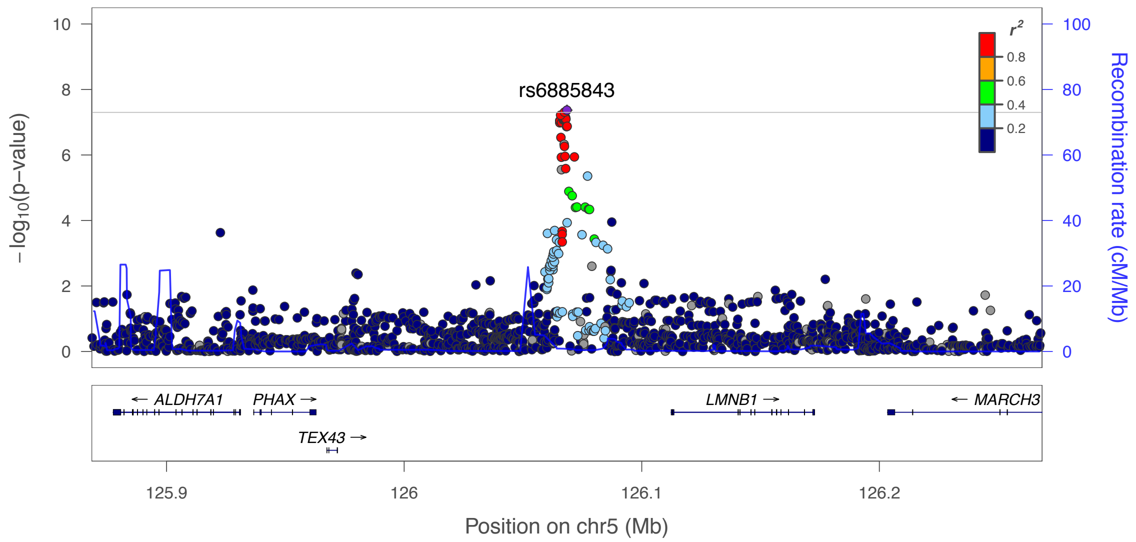

H

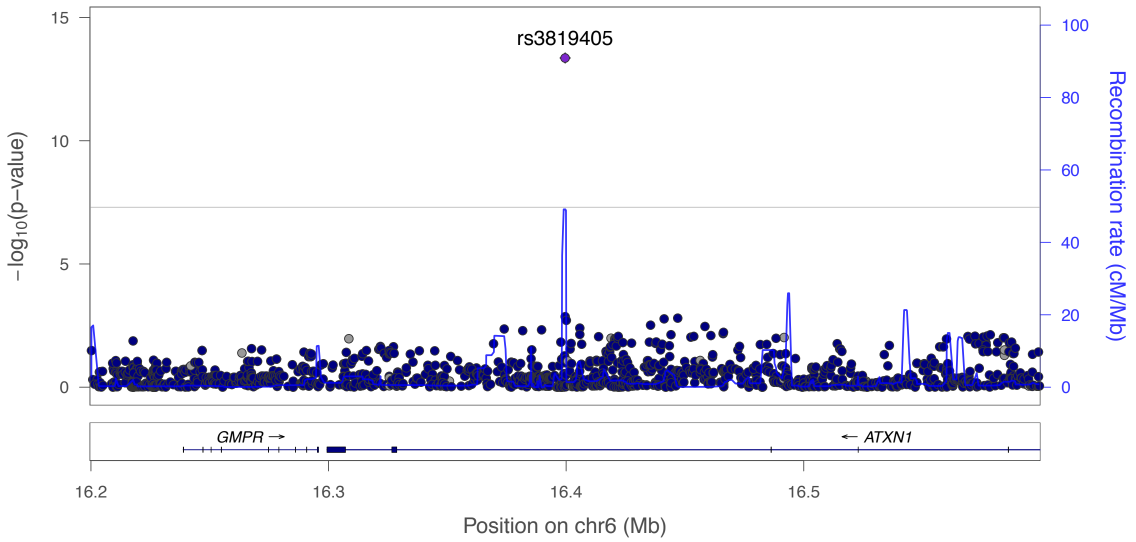

I

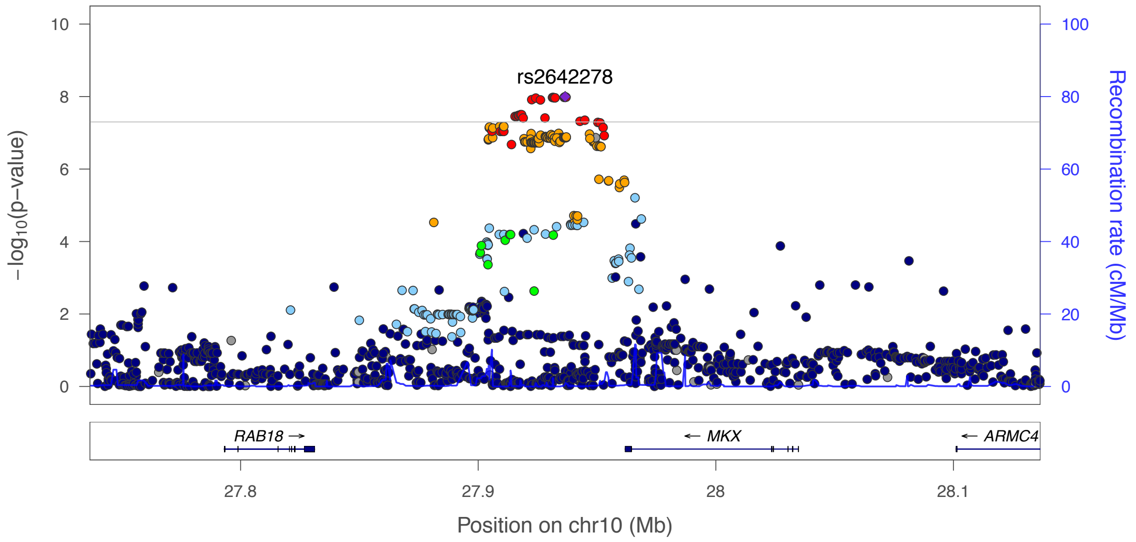

Supplementary Figure 3: Continued from previous page.

J

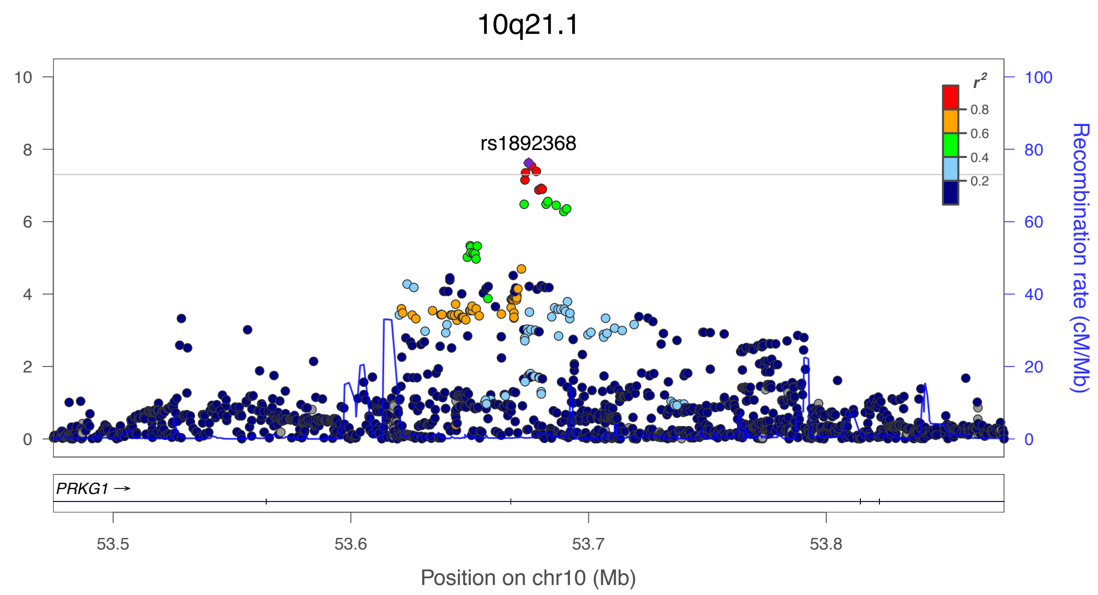

K

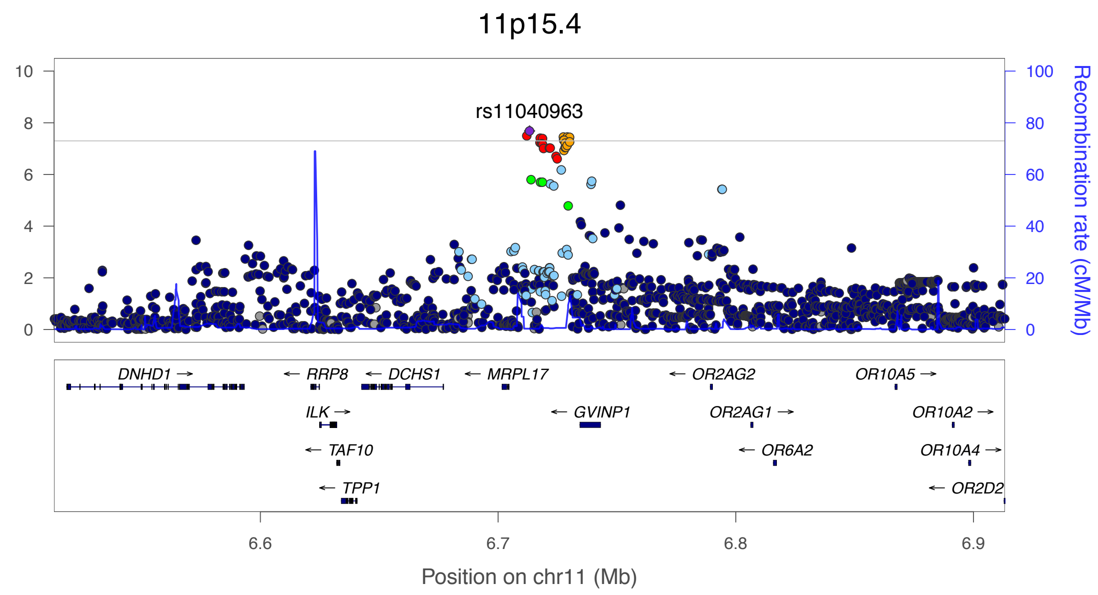

L

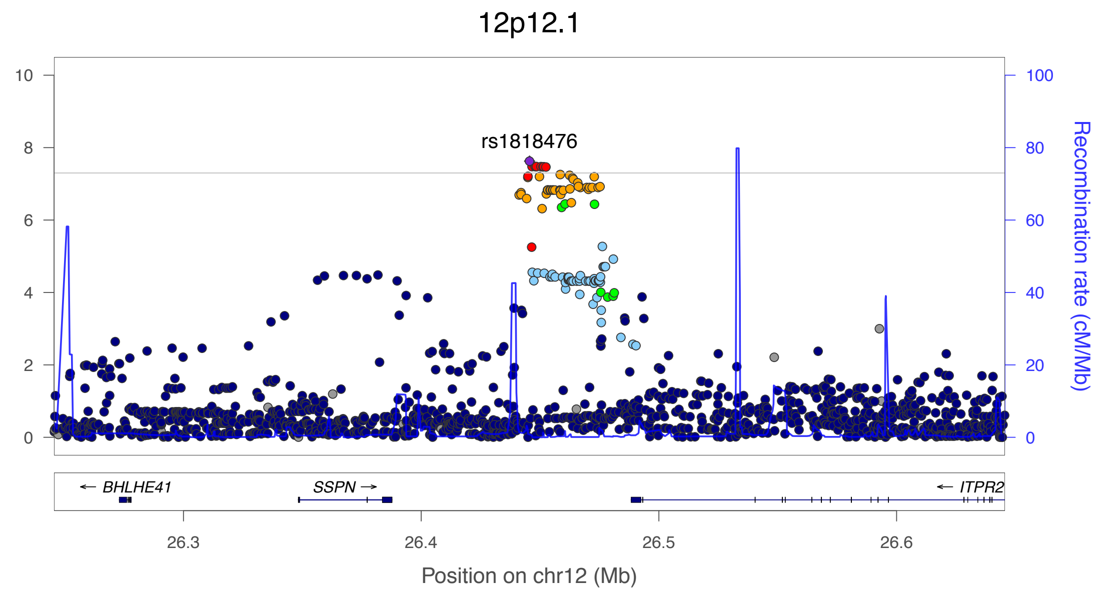

Supplementary Figure 3: Continued from previous page.

M

14q24.1

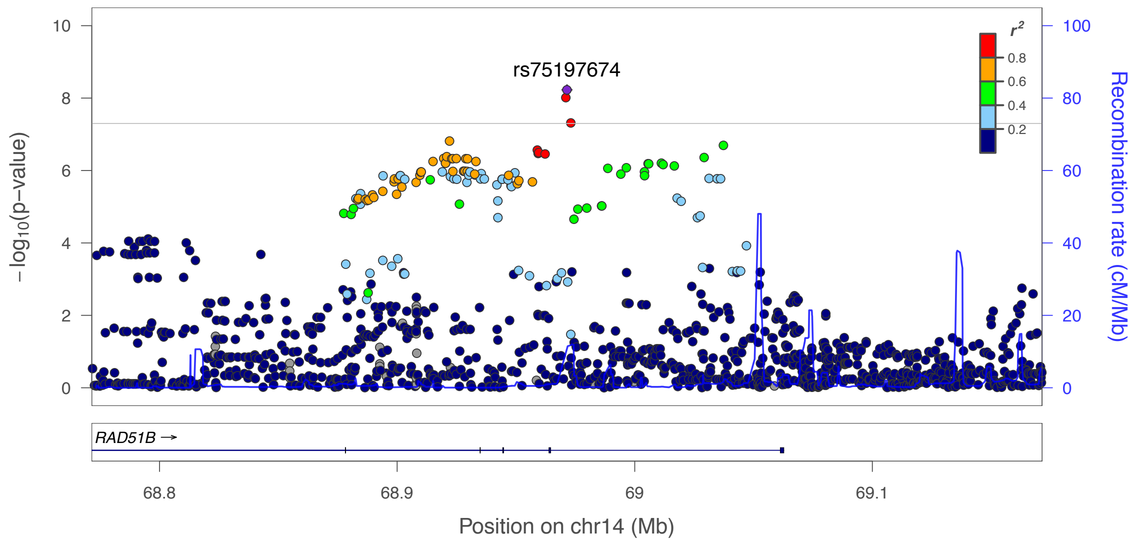

N

18q21.33

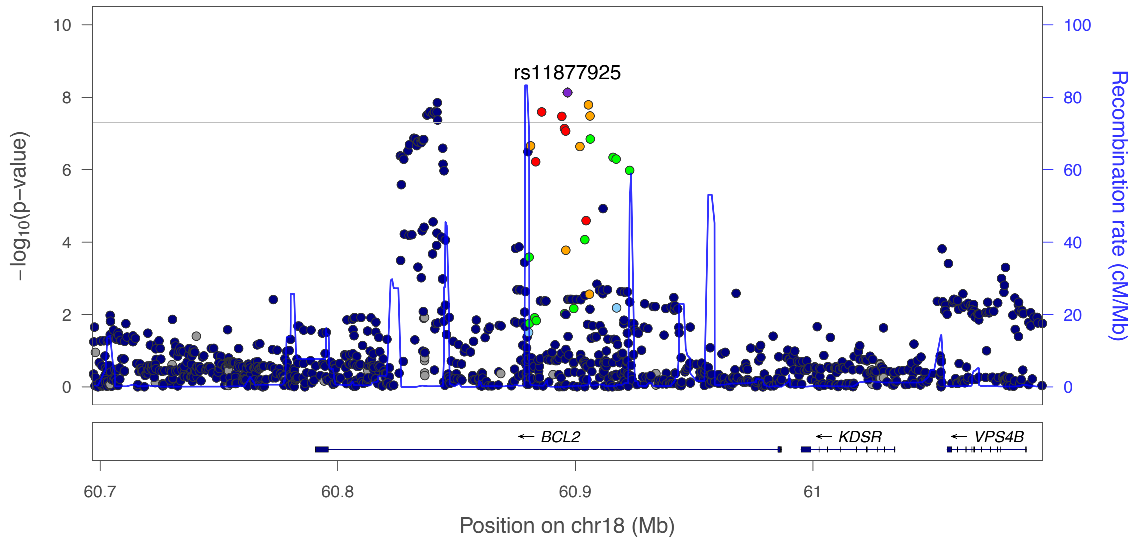

O

19q13.33

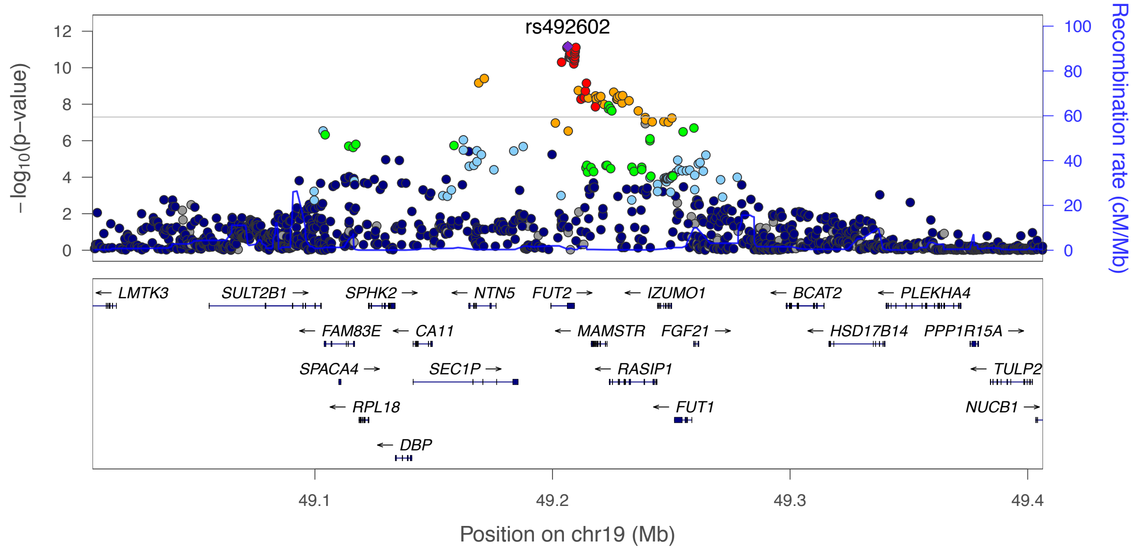

Supplementary Figure 3: Continued from previous page.

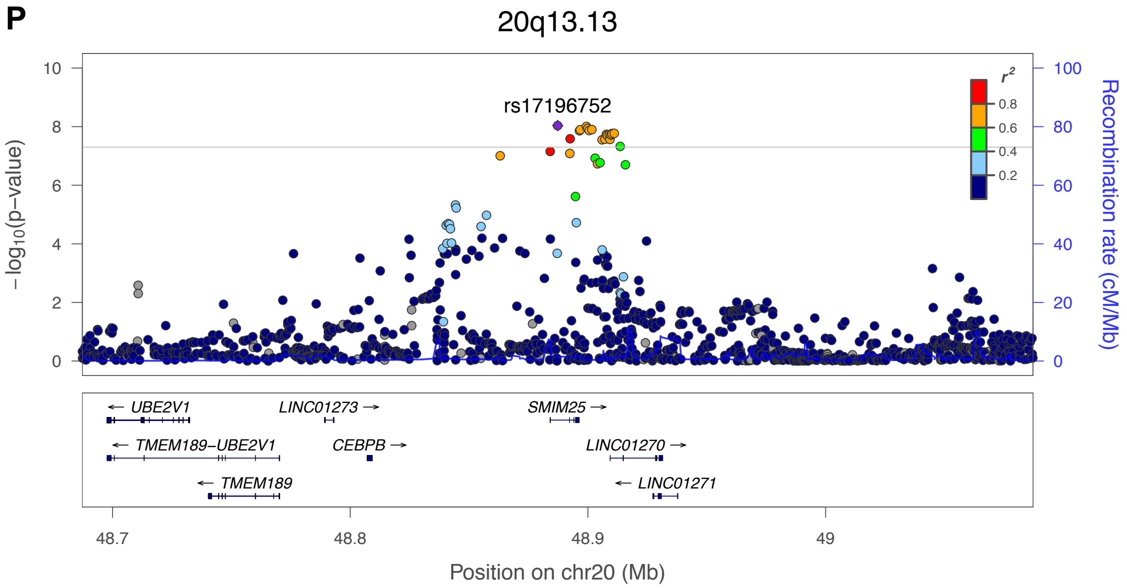

Supplementary Figure 3: Continued from previous page.

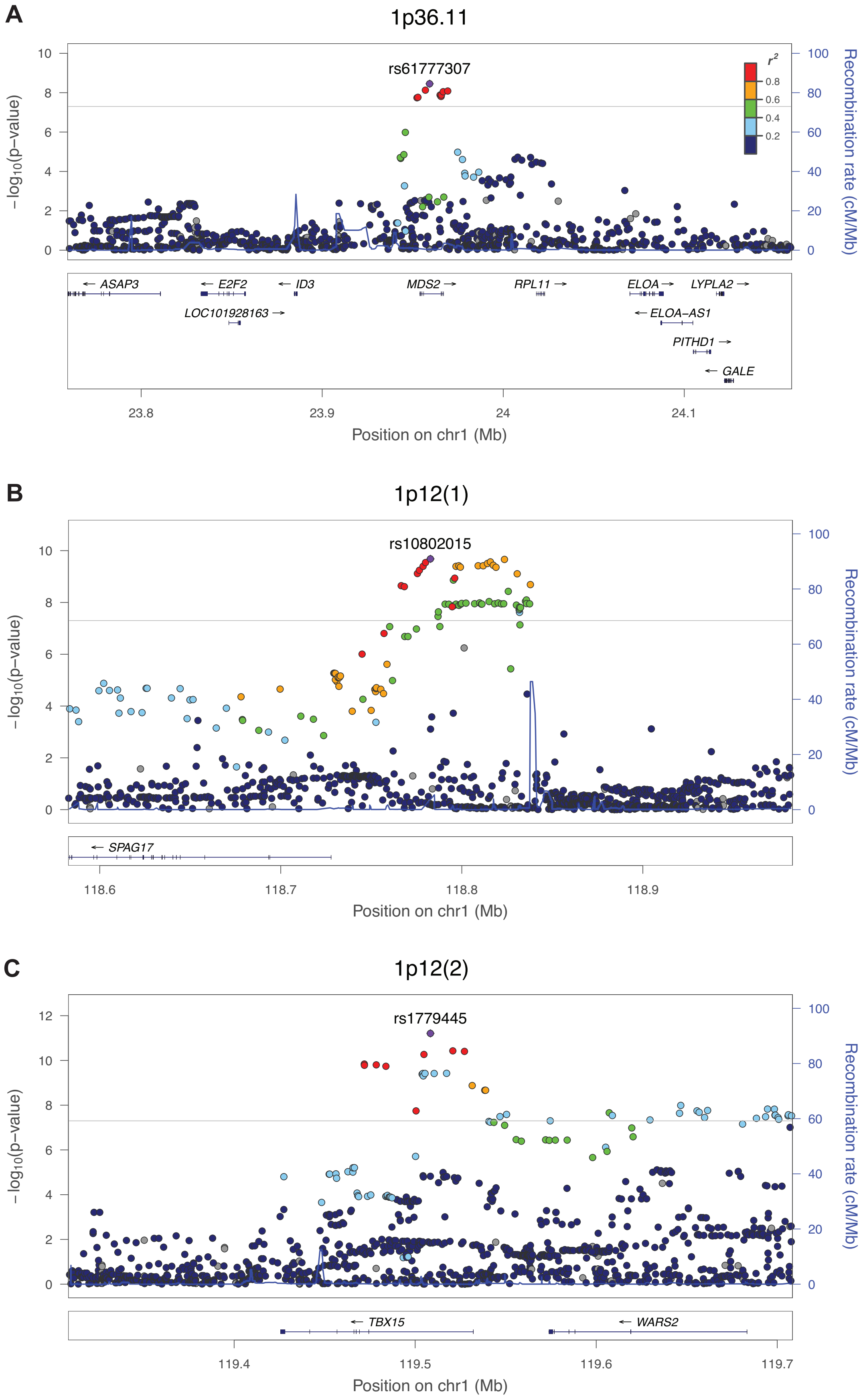

Supplementary Figure 4: Regional association plots for 13 loci (**A**-**M**) associated with nondense area. Colors indicate linkage disequilibrium (LD; r^2) in Europeans with the referent SNP (purple), which was the top SNP except where a proxy (parentheses) was used because LD information was unavailable (grey) for the top SNP. Previously reported SNPs (*italics*) within 200 kb and in low LD (r^2 <0.1) with the top SNP are shown in magenta.

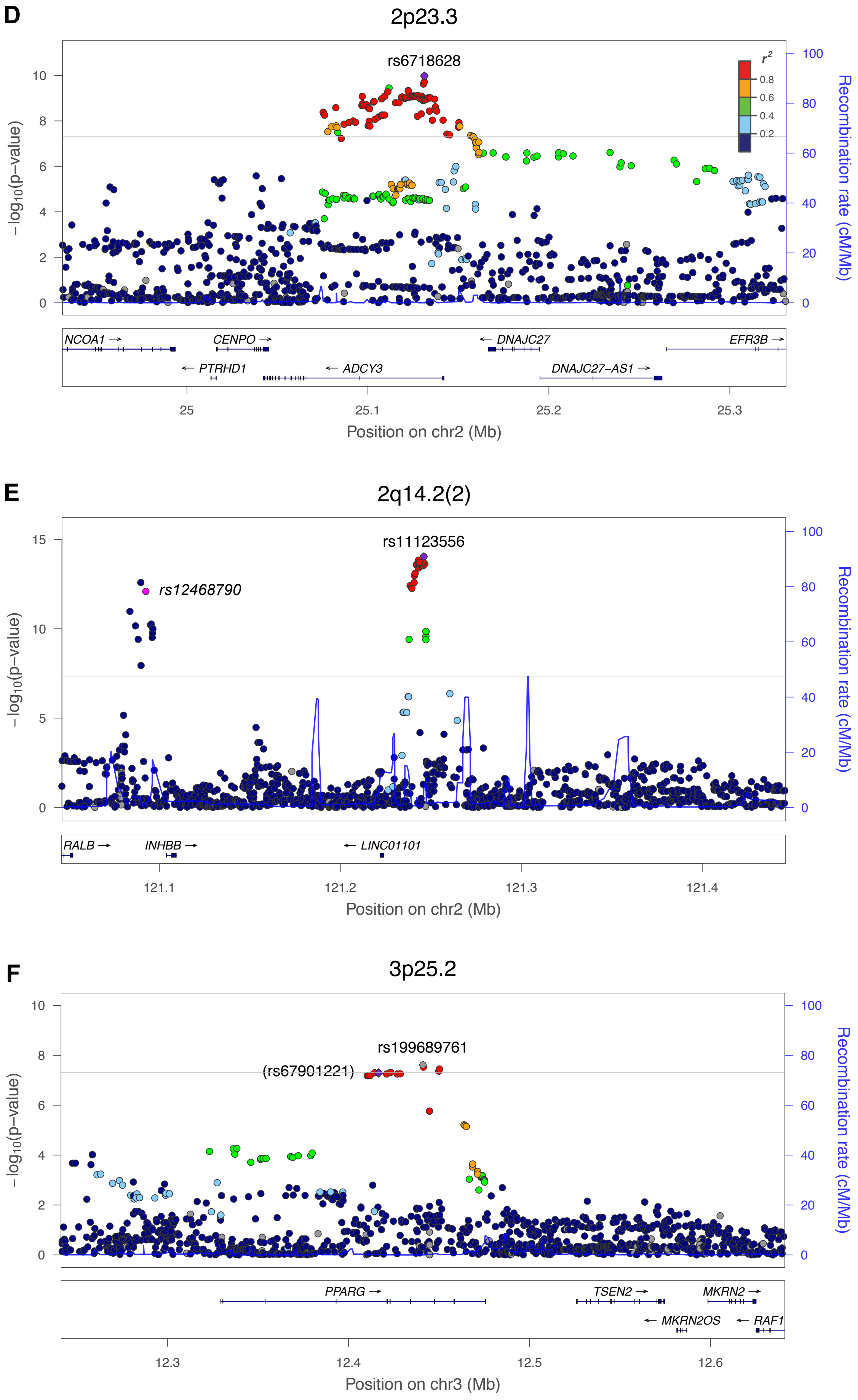

Supplementary Figure 4: Continued from previous page.

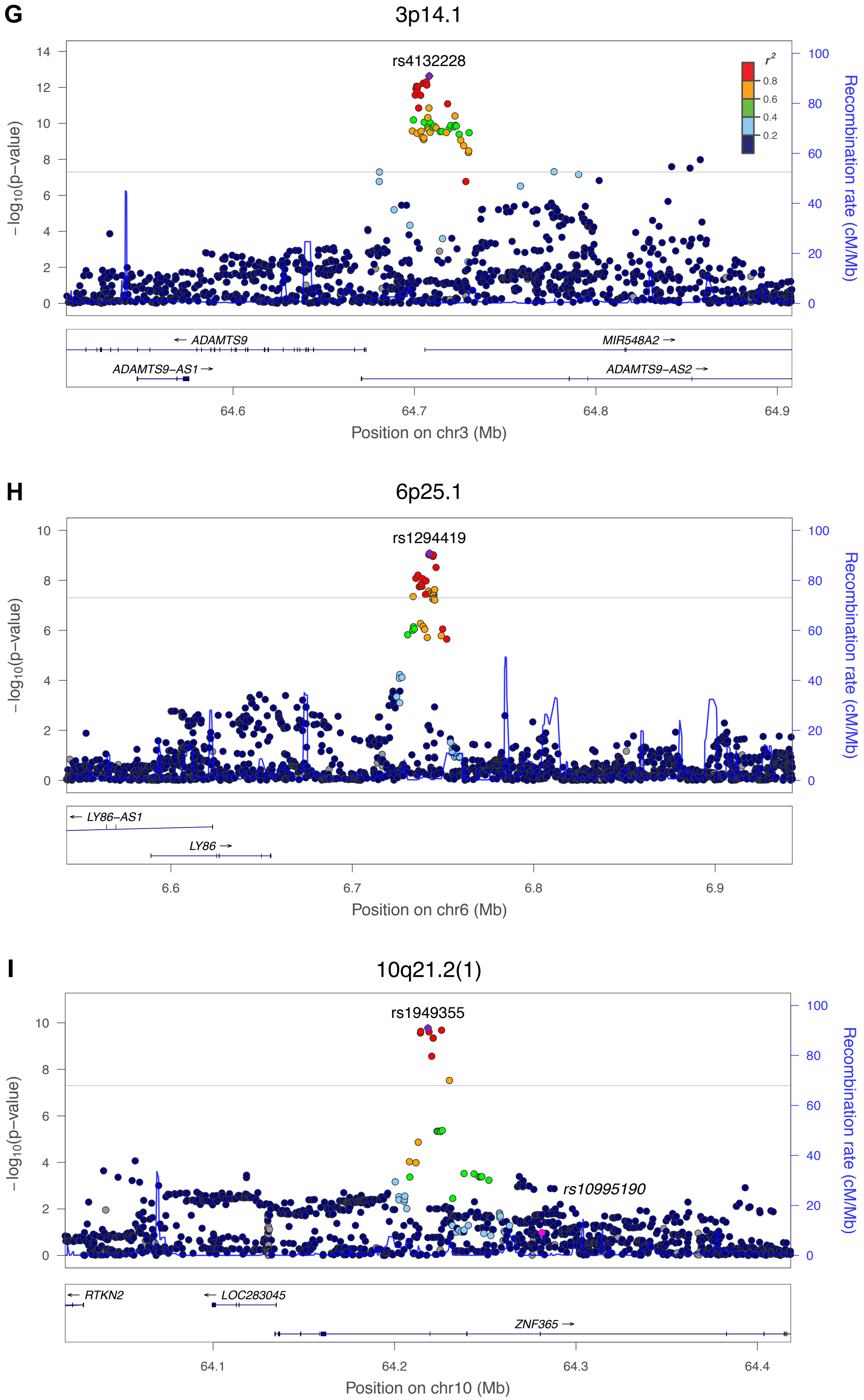

Supplementary Figure 4: Continued from previous page.

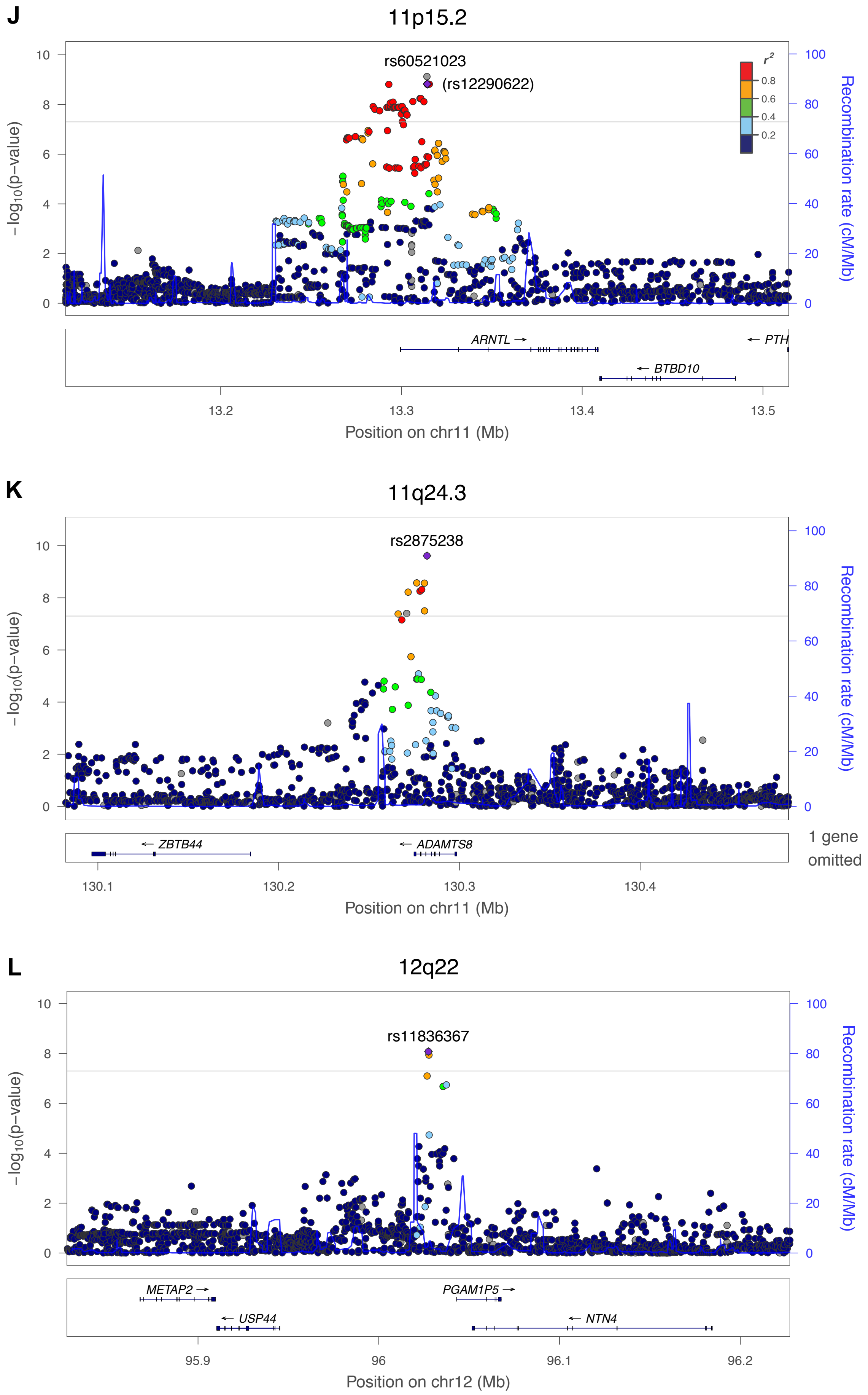

Supplementary Figure 4: Continued from previous page.

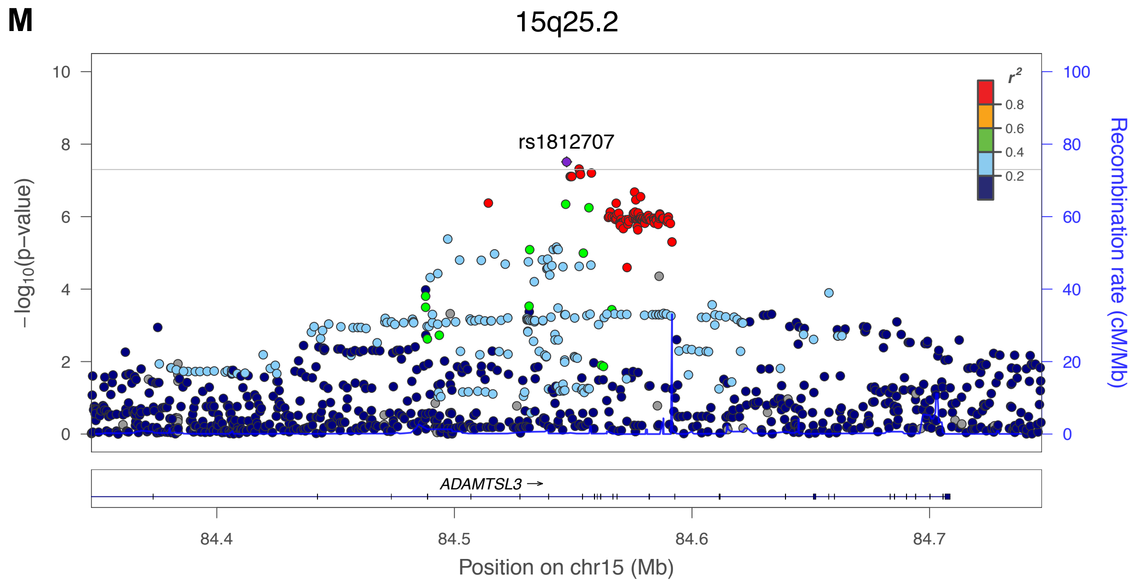

Supplementary Figure 4: Continued from previous page.

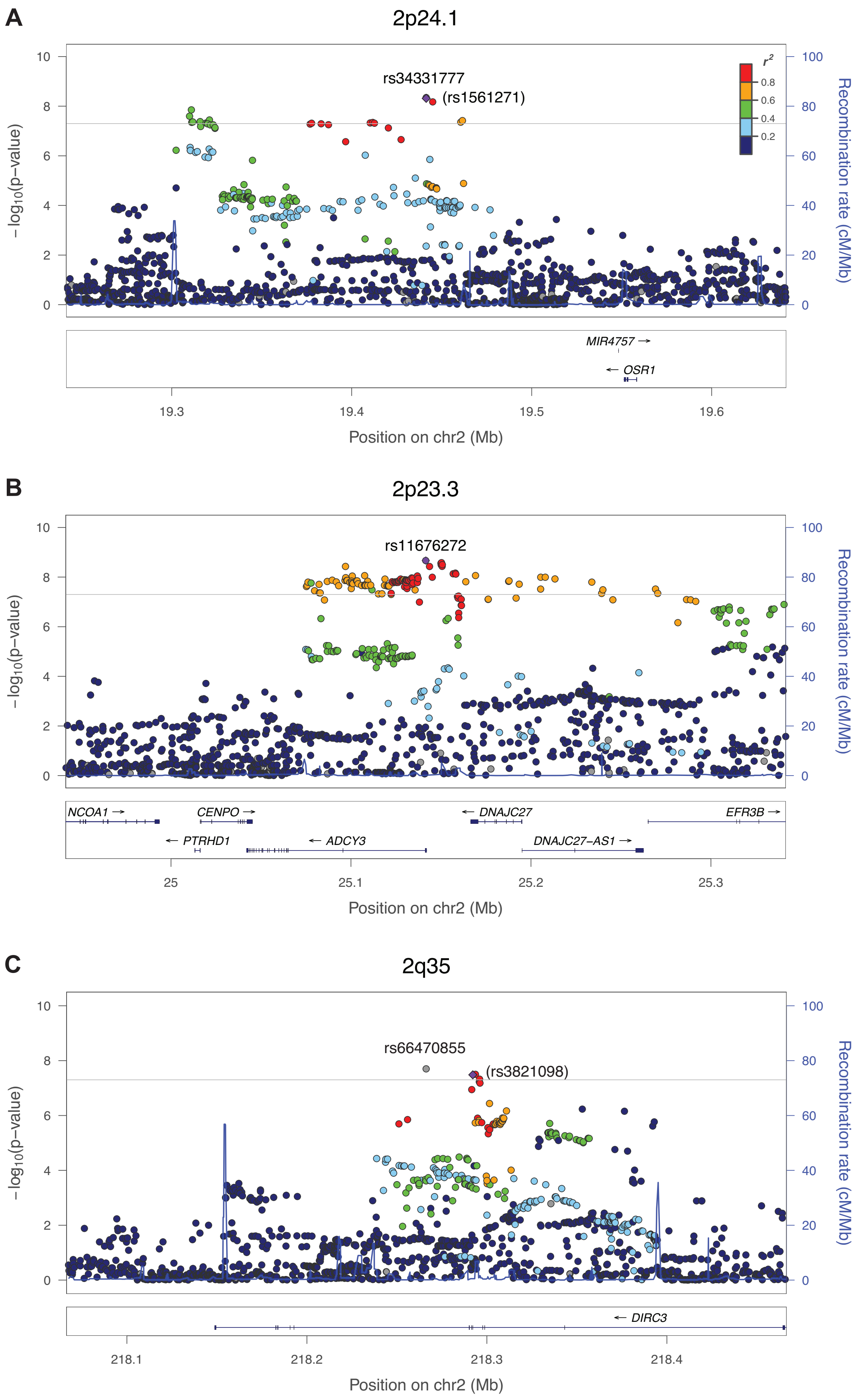

Supplementary Figure 5: Regional association plots for 12 loci (**A-L**) associated with percent density. Colors indicate linkage disequilibrium (LD; r^2) in Europeans with the referent SNP (purple), which was the top SNP except where a proxy (parentheses) was used because LD information was unavailable (grey) for the top SNP. Previously reported SNPs (*italics*) within 200 kb and in low LD (r^2 <0.1) with the top SNP are shown in magenta.

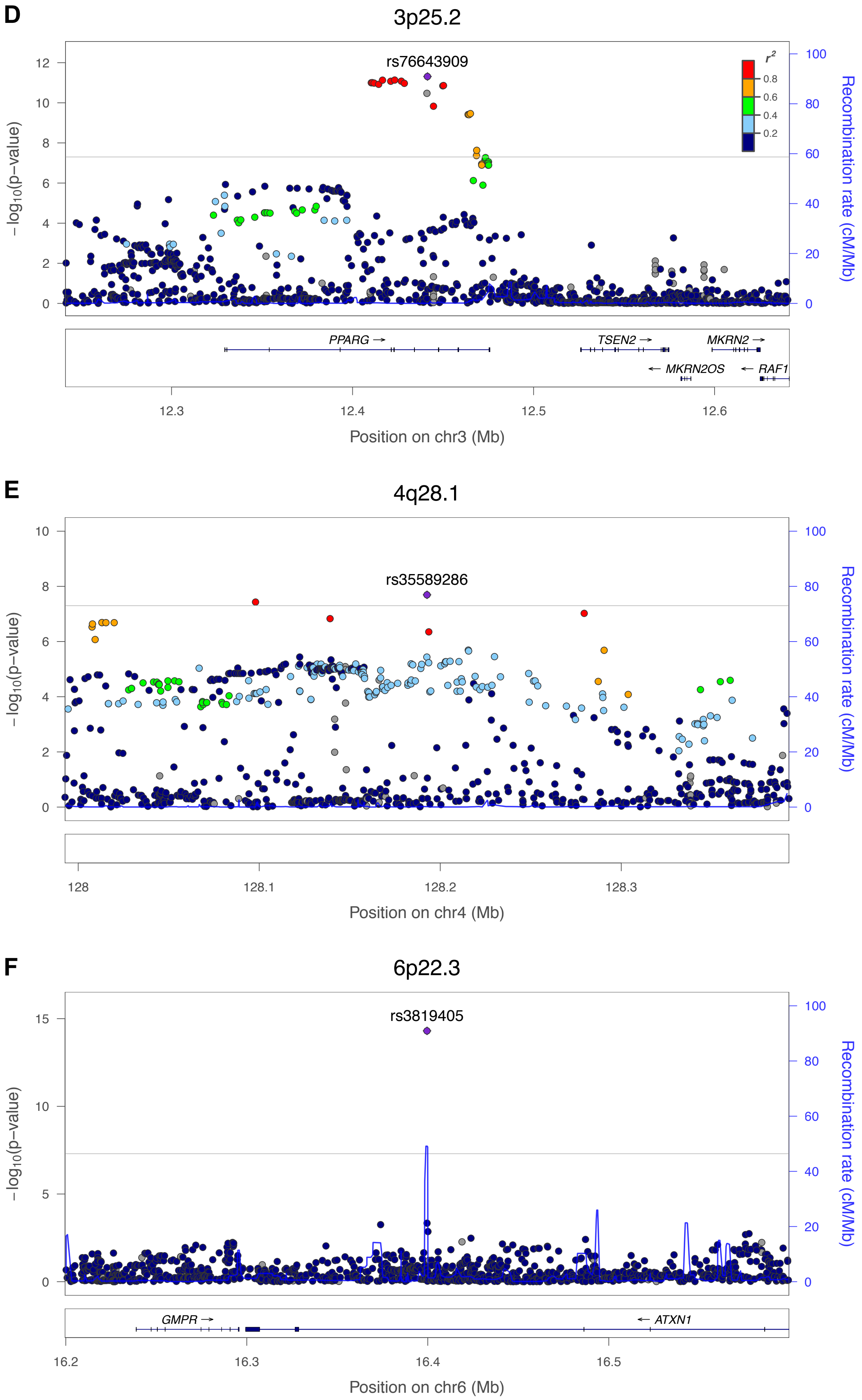

Supplementary Figure 5: Continued from previous page.

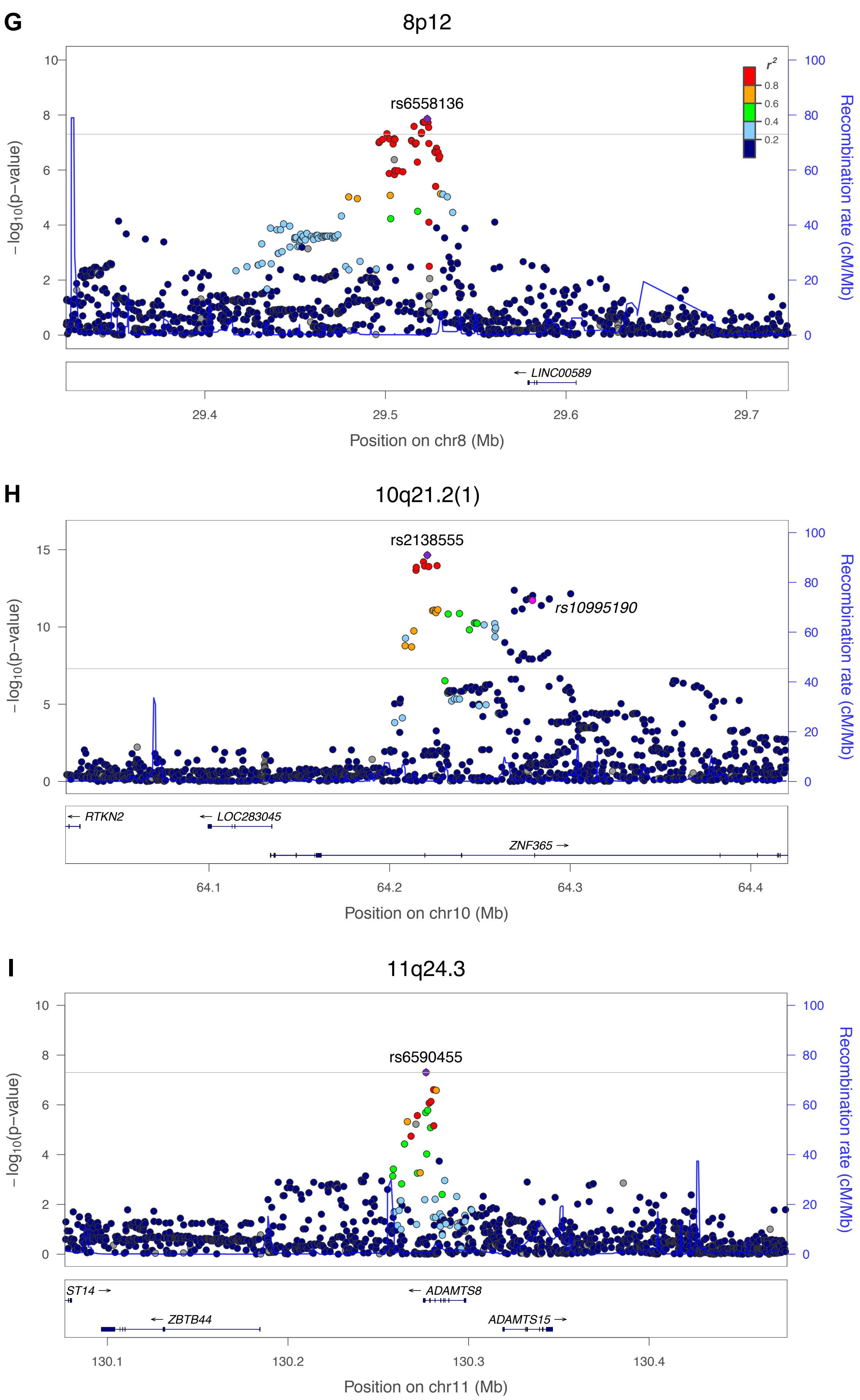

Supplementary Figure 5: Continued from previous page.

J

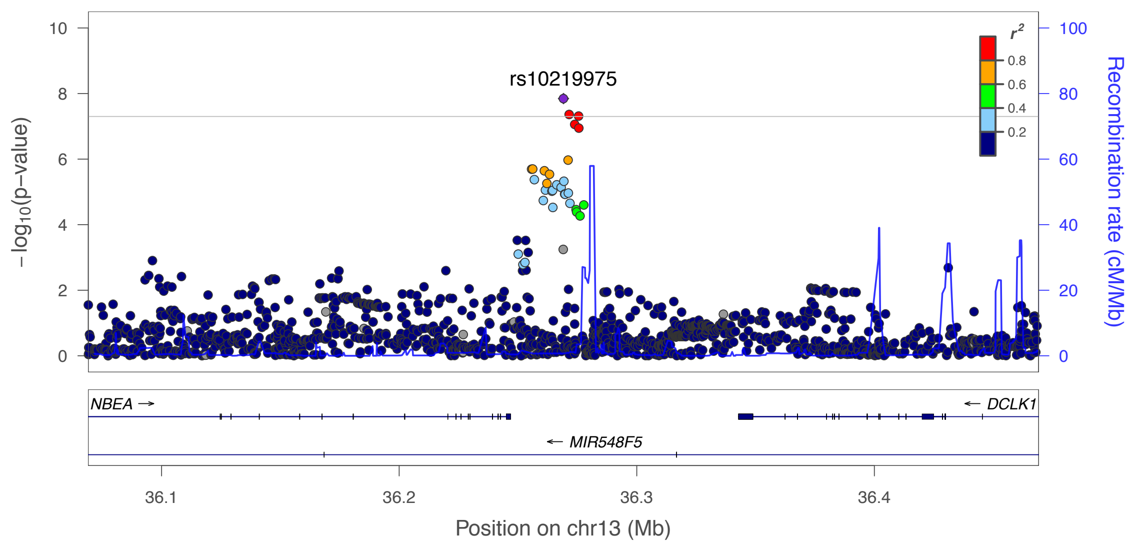

K

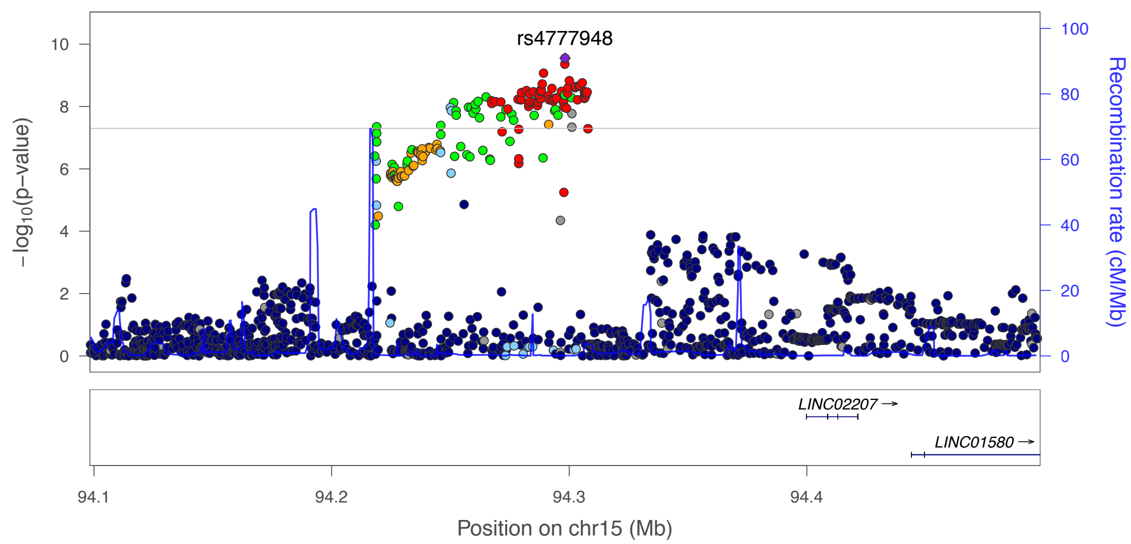

L

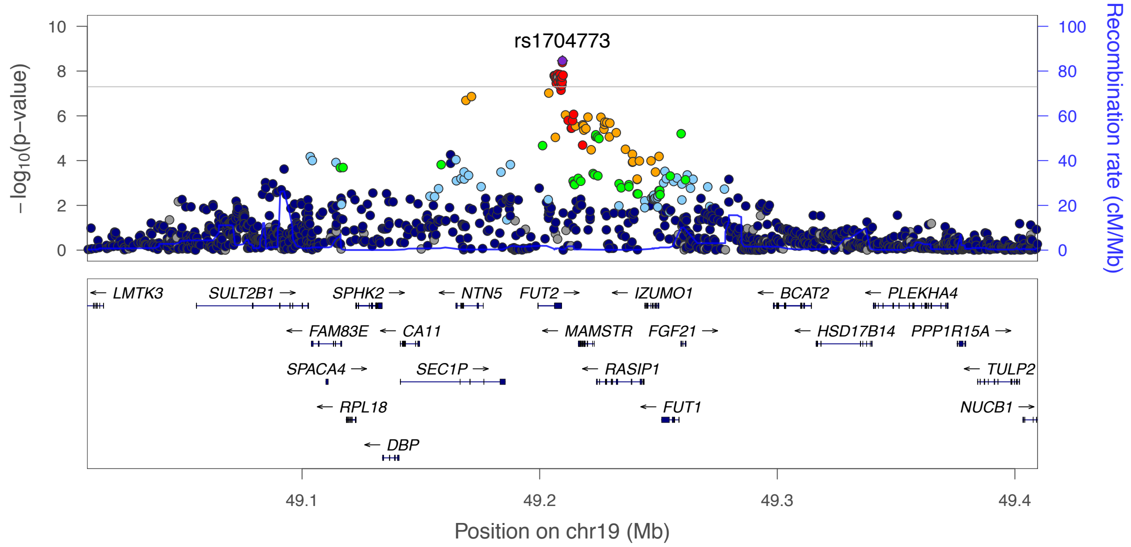

Supplementary Figure 5: Continued from previous page.

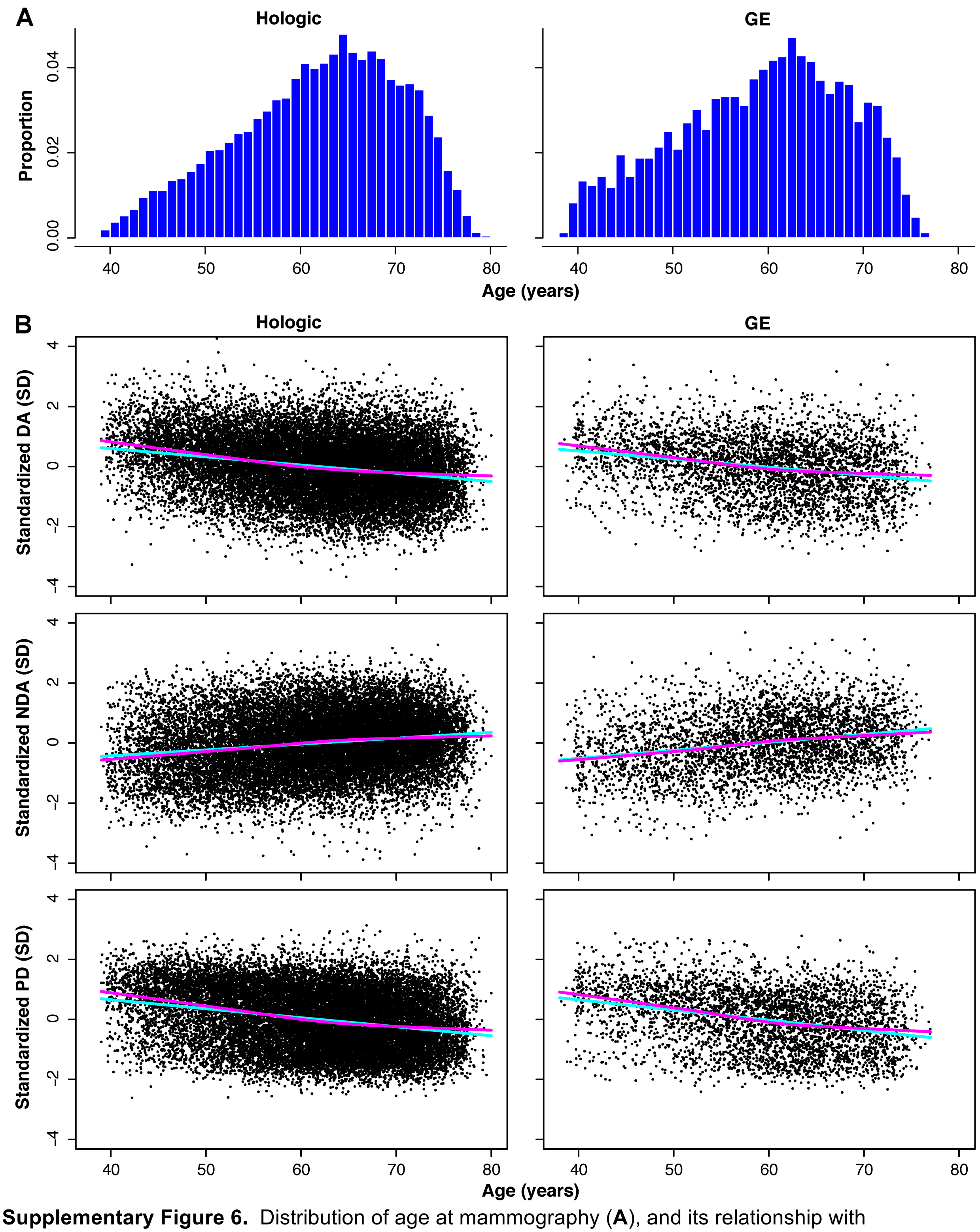

Supplementary Figure 6. Distribution of age at mammography (**A**), and its relationship with standardized measures of dense area (DA), nondense area (NDA) and percent density (PD) from Hologic and GE mammograms (**B**). DA, NDA and PD distributions were normalized using fifth-, cube- and cube-root transformations for Hologic images, and cube-, cube- and square-root transformations for GE images, respectively, and standardized to attain a mean of zero and variance of one. Linear (cyan) and loess (magenta) fits to the data show little departure from a linear relationship.

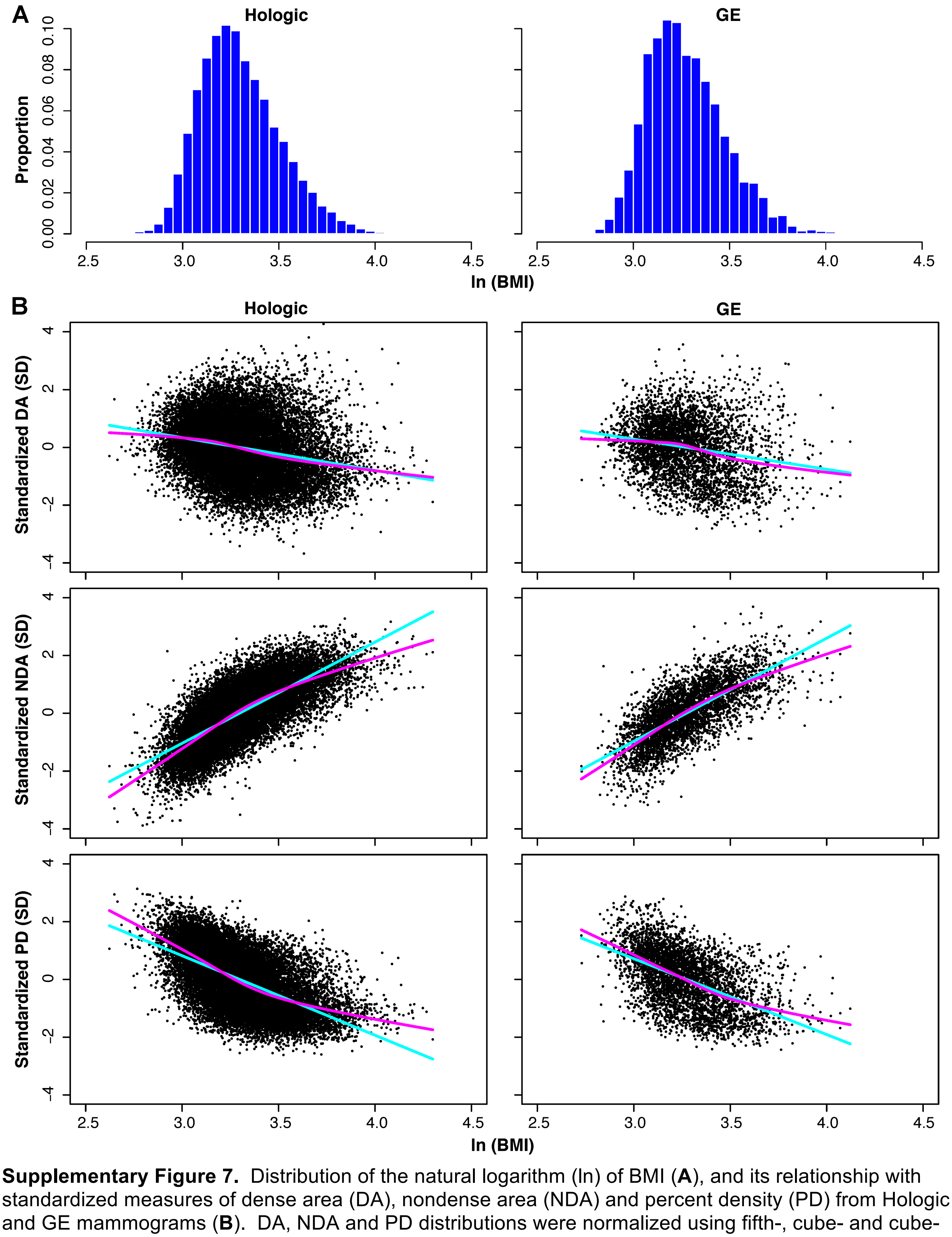

Supplementary Figure 7. Distribution of the natural logarithm (ln) of BMI (**A**), and its relationship with standardized measures of dense area (DA), nondense area (NDA) and percent density (PD) from Hologic and GE mammograms (**B**). DA, NDA and PD distributions were normalized using fifth-, cube- and cube-root transformations for Hologic images, and cube-, cube- and square-root transformations for GE images, respectively, and standardized to attain a mean of zero and variance of one. Linear (cyan) and loess (magenta) fits to the data show little departure from linearity except in the extreme tails of the distribution.
