# Supplementary material for: Identification of 31 loci for mammographic density phenotypes and their associations with breast cancer risk
Source: Nat Commun. 2020 Oct 9;11:5116. doi: 10.1038/s41467-020-18883-x (PMC7547012; doi:10.1038/s41467-020-18883-x)
Supplement: Supplementary file 2 — Description of Additional Supplementary Files [file 41467_2020_18883_MOESM2_ESM.pdf]

Description of Additional Supplementary Files

Supplementary Data 1

Description: Gene set enrichment analysis of candidate genes for all MD phenotypes (DA, NDA and PD).
