# Supplementary material for: Identification of 31 loci for mammographic density phenotypes and their associations with breast cancer risk
Source: Nat Commun. 2020 Oct 9;11:5116. doi: 10.1038/s41467-020-18883-x (PMC7547012; doi:10.1038/s41467-020-18883-x)
Supplement: Supplementary file 4 — Reporting Summary [file 41467_2020_18883_MOESM4_ESM.pdf]

nature research

Corresponding author(s): Weiva Sieh
Last updated by author(s): Sep 12, 2020

Reporting Summary

Nature Research wishes to improve the reproducibility of the work that we publish. This form provides structure for consistency and transparency in reporting. For further information on Nature Research policies, see our Editorial Policies and the Editorial Policy Checklist.

Statistics

For all statistical analyses, confirm that the following items are present in the figure legend, table legend, main text, or Methods section.

n/a	Confirmed	
☐	☒	The exact sample size (*n*) for each experimental group/condition, given as a discrete number and unit of measurement
☐	☒	A statement on whether measurements were taken from distinct samples or whether the same sample was measured repeatedly
☐	☒	The statistical test(s) used AND whether they are one- or two-sided<br>*Only common tests should be described solely by name; describe more complex techniques in the Methods section.*
☐	☒	A description of all covariates tested
☐	☒	A description of any assumptions or corrections, such as tests of normality and adjustment for multiple comparisons
☐	☒	A full description of the statistical parameters including central tendency (e.g. means) or other basic estimates (e.g. regression coefficient) AND variation (e.g. standard deviation) or associated estimates of uncertainty (e.g. confidence intervals)
☐	☒	For null hypothesis testing, the test statistic (e.g. *F*, *t*, *r*) with confidence intervals, effect sizes, degrees of freedom and *P* value noted<br>*Give P values as exact values whenever suitable.*
☒	☐	For Bayesian analysis, information on the choice of priors and Markov chain Monte Carlo settings
☒	☐	For hierarchical and complex designs, identification of the appropriate level for tests and full reporting of outcomes
☐	☒	Estimates of effect sizes (e.g. Cohen's *d*, Pearson's *r*), indicating how they were calculated

Our web collection on statistics for biologists contains articles on many of the points above.

Software and code

Policy information about availability of computer code

Data collection	Cumulus6
Data analysis	R 3.3.3, PLINK v1.07, EIGENSOFT4.2, IMPUTE2.2.2, SHAPEIT v2.r644, METAL, LocusZoom v1.3, LDSC v1.01, Uncovering Enrichment through Simulation (UES), DAVID v6.8, GCTA v1.02

For manuscripts utilizing custom algorithms or software that are central to the research but not yet described in published literature, software must be made available to editors and reviewers. We strongly encourage code deposition in a community repository (e.g. GitHub). See the Nature Research guidelines for submitting code & software for further information.

Data

Policy information about availability of data

All manuscripts must include a data availability statement. This statement should provide the following information, where applicable:
- Accession codes, unique identifiers, or web links for publicly available datasets
- A list of figures that have associated raw data
- A description of any restrictions on data availability

Genotype data of RPGEH GERA participants are available from the database of Genotypes and Phenotypes (dbGaP) under accession phs000674.v3.p3 [https://www.ncbi.nlm.nih.gov/projects/gap/cgi-bin/study.cgi?study_id=phs000674.v3.p3]. This includes individuals who consented to having their data shared with dbGaP. The complete GERA data are available upon application to the KP Research Bank (https://researchbank.kaiserpermanente.org/our-research/for-researchers). Breast cancer summary statistics are available at http://bcac.ccge.medschl.cam.ac.uk/bcacdata. All remaining relevant data are available in the article, supplementary information, or from the corresponding author upon reasonable request.

Field-specific reporting

Please select the one below that is the best fit for your research. If you are not sure, read the appropriate sections before making your selection.

[x] Life sciences   [ ] Behavioural & social sciences   [ ] Ecological, evolutionary & environmental sciences

For a reference copy of the document with all sections, see nature.com/documents/nr-reporting-summary-flat.pdf

Life sciences study design

All studies must disclose on these points even when the disclosure is negative.

Sample size	This study included 24,192 women.
Data exclusions	This study included all non-Hispanic white women in the KPNC GERA cohort with the available screening mammograms, genotype and EHR data required for this study.
Replication	In this GWAS meta-analysis, the conventional genome-wide significance level of 5×10-8 was used to determine statistical significance. SNPs that reached genome-wide significance in one study but not in the combined meta-analysis were considered nonsignificant.
Randomization	Not applicable. This study was not a randomized clinical trial.
Blinding	Not applicable. This study did not involve experimental group allocation.

Reporting for specific materials, systems and methods

We require information from authors about some types of materials, experimental systems and methods used in many studies. Here, indicate whether each material, system or method listed is relevant to your study. If you are not sure if a list item applies to your research, read the appropriate section before selecting a response.

Materials & experimental systems

n/a	Involved in the study	
[x]	[ ]	Antibodies
[x]	[ ]	Eukaryotic cell lines
[x]	[ ]	Palaeontology and archaeology
[x]	[ ]	Animals and other organisms
[ ]	[x]	Human research participants
[x]	[ ]	Clinical data
[x]	[ ]	Dual use research of concern

Methods

n/a	Involved in the study	
[x]	[ ]	ChIP-seq
[x]	[ ]	Flow cytometry
[x]	[ ]	MRI-based neuroimaging

Human research participants

Policy information about studies involving human research participants

Population characteristics	This population-based study included non-Hispanic white women 40-80 years of age in the KPNC Genetic Epidemiology Research on Adult health and Aging (GERA) study who underwent digital screening mammography.
Recruitment	This study did not recruit participants, but was ancillary to the parent KPNC GERA study.
Ethics oversight	Institutional Review Board approvals were obtained from from Kaiser Permanente Northern California, Stanford University, and the Icahn School of Medicine at Mount Sinai.

Note that full information on the approval of the study protocol must also be provided in the manuscript.
